# Small Extracellular Vesicles‐Derived Circ6718 Unlocks Stromal Remodeling and Serves as a Biomarker in Gastric Cancer

**DOI:** 10.1002/advs.202521334

**Published:** 2026-05-11

**Authors:** Fan Zhang, Xueyan Zang, Dongli Wang, Rong Li, Hui Qian, Wenrong Xu, Jiajia Jiang, Yongmin Yan

**Affiliations:** ^1^ Department of Laboratory Medicine Wujin Hospital Affiliated With Jiangsu University 2 North Yongning Road Changzhou Jiangsu P. R. China; ^2^ Jiangsu Key Laboratory of Medical Science and Laboratory Medicine Department of Laboratory Medicine School of Medicine Jiangsu University Zhenjiang Jiangsu P. R. China; ^3^ Aoyang Institute of Cancer, Department of Laboratory Medicine Affiliated Aoyang Hospital of Jiangsu University Suzhou Jiangsu P. R. China

**Keywords:** circ6718, engineering transformation, gastric cancer, GC‐MSCs, molecular biomarker, sEVs

## Abstract

Small extracellular vesicles (sEVs)‐derived circular RNA (circRNA) serves as a crucial biomarker for diagnosing gastric cancer and as a key regulator of tumor progression, orchestrating intercellular crosstalk within the tumor microenvironment (TME). In gastric cancer (GC), tissue‐derived mesenchymal stem cells (GC‐MSCs) critically drive tumor progression; however, the interplay between sEVs and circRNA in GC‐MSCs remains incompletely understood. We identified sEVs‐hsa_circ_0006718 (circ6718) as significantly upregulated in gastric cancer patients. Elevated levels of sEVs‐circ6718 correlated with clinical stage, distant metastasis and poor prognosis, confirming its utility as both an early diagnostic and prognostic biomarker in GC. Mechanistically, circ6718 functions as a competing endogenous RNA (ceRNA) by sequestering hsa‐miR‐561‐3p, thereby derepressing the expression of SAAL1 (Serum amyloid A‐like 1). SAAL1 enhances the transcriptional activity of PRRX1 (Paired related homeobox 1), which directly activates the TGFβ1 promoter. Consequently, the TGFβ1/Smad2/3 signaling pathway drives the transdifferentiation of GC‐MSCs into cancer‐associated fibroblasts (CAFs)—promoting stromal remodeling and tumor aggressiveness. Our findings unveil a novel sEVs‐circRNA‐mediated axis in GC progression, revealing dual utility in diagnostics and targeted therapy.

## Graphical abstract

sEVs‐circ6718 serves as a promising serum biomarker for gastric cancer early diagnosis and prognosis, promoting malignant transformation in gastric cancer cells and GC‑MSC differentiation into CAFs.

sEVs‑circ6718 holds potential as a biomarker for the early diagnosis and prognostic assessment of gastric cancer. Its oncogenic mechanism involves targeting the hsa‑miR‑561‑3p/SAAL1/PRRX1 axis, thereby activating the TGFβ1/Smad2/3 signaling pathway to promote gastric cancer cell proliferation, invasion, and metastasis, as well as the conversion of tumor stromal GC‑MSCs into cancer‑associated fibroblasts (CAFs). Furthermore, sEVs derived from HEK293T cells loaded with circ6718 siRNA (sEVs@circ6718 si) can remodel the tumor stromal microenvironment and suppress gastric cancer progression.

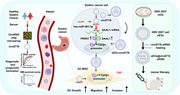

## 1 Introduction

Gastric cancer (GC) is the fifth most prevalent cancer and the fourth leading cause of cancer‐related mortality worldwide [1]. It is often diagnosed at advanced stages due to the asymptomatic nature of early disease and the absence of reliable biomarkers. This underscores the urgent necessity for the identification of more effective biomarkers and therapeutic targets for the early detection and treatment of GC. The tumor microenvironment (TME) is a highly dynamic and heterogeneous niche composed of tumor cells and various non‐malignant cell types, including immune cells, endothelial cells, and stromal components such as fibroblasts. Among these components, cancer‐associated fibroblasts (CAFs) constitute a major cellular element that actively contributes to tumor progression through extracellular matrix remodeling, promotion of angiogenesis, modulation of immune responses, and enhancement of tumor invasion and metastasis [2, 3]. Additionally, mesenchymal stromal cells (MSCs) within the stroma significantly contribute to tumorigenesis and progression [4, 5]. Recruited MSCs can differentiate into CAFs, primarily driven by the transforming growth factor‐beta (TGF‐β) signaling pathway [6, 7, 8], establishing the MSC–CAF axis as a promising therapeutic target [9]. Our previous research identified and isolated gastric cancer tissue‐derived mesenchymal stem cells (GC‐MSCs) as key components of the TME. Our study demonstrated that GC‐MSCs enhance GC cell proliferation, migration, lymphatic metastasis, angiogenesis, and macrophage M2 polarization through paracrine signaling [10, 11]. However, the mechanisms by which GC cells modulate the phenotype and function of GC‐MSCs remain largely unexplored. Elucidating these interactions may unveil novel strategies to disrupt tumor–stroma crosstalk.

sEVs play a pivotal role in intercellular communication and have been linked to the progression of cancer and various other disease [12, 13]. They facilitate the exchange of information between tumor and non‐tumor cells within the TME, significantly influencing tumor growth, metastasis, metabolism, immunosuppression, and drug resistance [14, 15, 16, 17, 18, 19]. The proteins, microRNAs (miRNAs), circular RNAs (circRNAs), and other bioactive components present in sEVs are crucial for cancer progression, diagnosis, prognosis, and treatment [20, 21, 22]. sEVs orchestrate intricate interactions among tumor cells, immune cells, and stromal cells [23, 24, 25]; however, the underlying mechanisms warrant further investigation.

CircRNAs, characterized by their covalently closed loop structure, exhibit remarkable structural stability, abundance, conservation, and spatiotemporal specificity, thereby playing a significant role in cancer progression [26]. CircRNAs regulate gene expression at transcriptional, post‐transcriptional and translational levels. They can influence tumor growth, metastasis, recurrence, drug resistance, metabolic dysregulation, and immune escape by functioning as miRNA sponges, protein scaffolds, regulators of protein phase separation, modulators of mRNA stability, and translators of peptides, among other mechanisms. Consequently, they represent novel biomarkers and therapeutic targets for liquid biopsy [27, 28].

In this study, we identified various circRNAs present in serum‐derived sEVs from gastric cancer patients using circRNA microarrays. Our results demonstrated that circ6718 is significantly upregulated in gastric cancer serum sEVs. Elevated levels of sEVs‐circ6718 are associated with distal metastasis and poorer prognosis in gastric cancer patients, indicating its oncogenic role in the progression of the disease. Mechanistically, circ6718 functions as a sponge for hsa‐miR‐561‐3p, leading to the upregulation of SAAL1 expression. SAAL1 enhances the transcriptional activity of PRRX1, which subsequently binds to and activates the transcription of the TGFβ1 promoter. This cascade activates the TGFβ1/Smad2/3 signaling pathway, driving the differentiation of GC‐MSCs into the CAFs, thereby promoting stromal remodeling and the progression of GC.

## 2 Results

### 2.1 sEVs‐circ6718 Serves as a Gastric Cancer Diagnostic and Prognostic Biomarker

**FIGURE 1:**
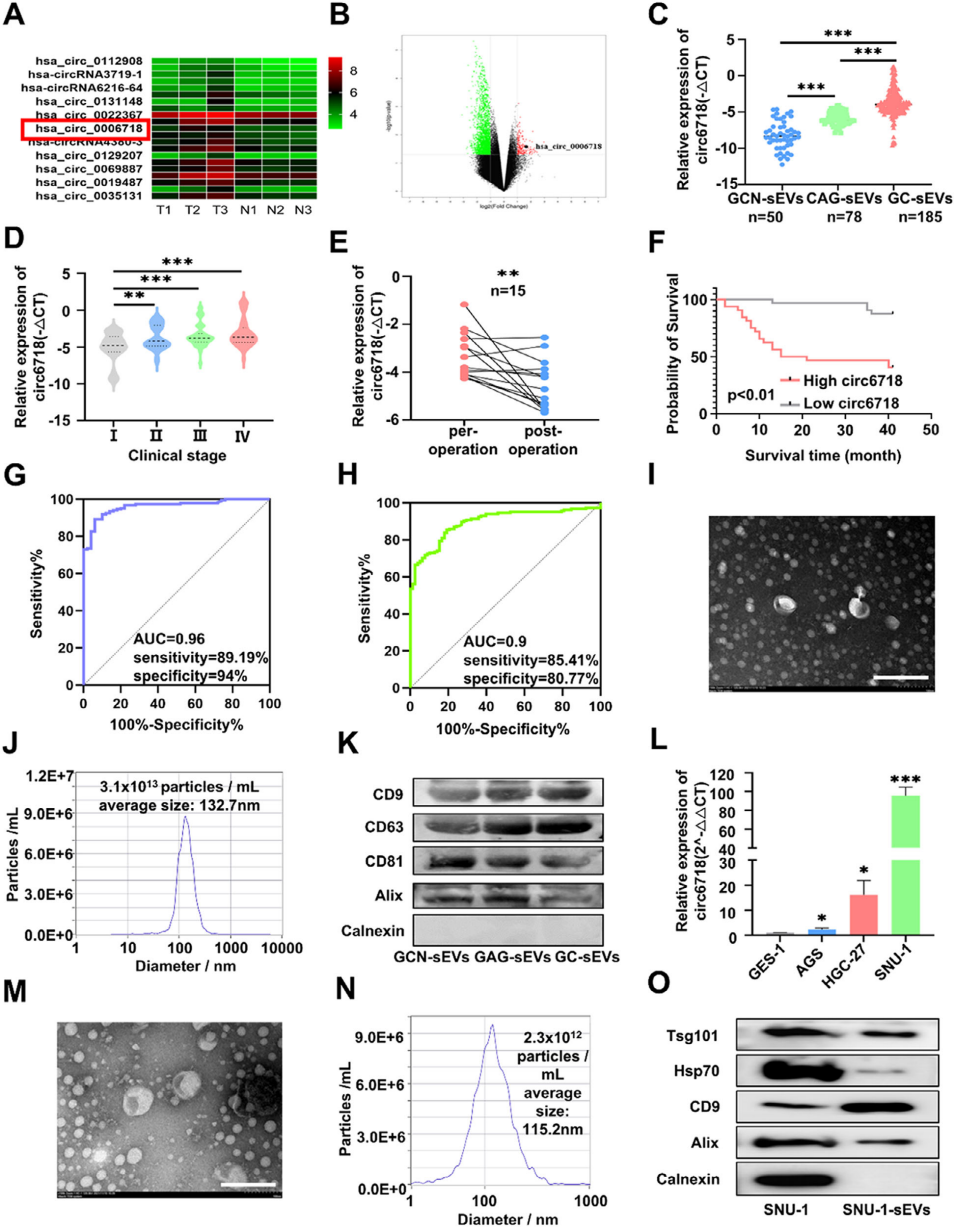
Circ6718 Is Upregulated in sEVs From GC Patient Serum and GC Cells, as a Promising Biomarker. (A) Heat Map Representation of circ6718 Expression in Microarray Assay Results (n = 6). (B) Volcano Plot Illustrating the Expression Levels of circ6718 in Microarray Assay Results (n = 6). (C) Expression Levels of circ6718 in Serum sEVs From Healthy Individuals, Patients With Chronic Atrophic Gastritis, and GC Patients (n = 50, 78 and 185). (D) Expression Levels of circ6718 in Plasma sEVs From GC Patients at Different Clinical Stages (n = 36, 37, 56, 56). (E) Expression of circ6718 in Serum sEVs From GC Patients Before and After Surgery (n = 15). (F) Kapla‐Meier Plot Analyzing the Association Between circ6718 Expression Levels and Overall Survival Time, With Log‐rank Tests Used to Determine Statistical Significance (n = 64). (G) Receiver Operating Characteristic Curves (ROC) Were Used to Assess circ6718 Levels in Serum sEVs From Healthy Individuals and GC Patients (n = 50 vs. 185, respectively). (H) Receiver Operating Characteristic Curves (ROC) Were Used to Evaluate Serum sEVs‐circ6718 Levels in Patients With Chronic Atrophic Gastritis versus GC Patients (n = 78 vs. 185, respectively). (I) TEM Analysis of Serum sEVs From GC Patients (scale bar = 100 nm). (J) Nanoparticle Tracking Analyses Measuring the Size of Serum sEVs in GC Patients. (K) Western Blot Analyses of Serum sEVs in GC Patients. (L) Expression Levels of circ6718 in sEVs Derived From GC Cell Lines (n = 3). (M) TEM Analysis of GC Cell sEVs (scale bar = 100 nm). (N) Nanoparticle Tracking Analysis Showing the Size of sEVs in GC Cells. (O) Western Blot Analysis of Proteins in GC Cell sEVs. The Data Were Plotted as Mean ± SEM. Statistical Significance Is Indicated as Follows: *p < 0.05, **p < 0.01, ***p < 0.001 by Student's *t*‐Test for E; by One‐Way ANOVA for C, D and L. Survival Time (F) Was Analyzed Using the Kaplan–Meier Method and a Log‐rank Test.

The circRNA microarray analysis of serum‐derived sEVs from GC patients, compared to healthy controls identified hsa_circ_0006718 (circ6718) as significantly upregulated (Figure 1A,B). Bioinformatics characterization confirmed that circ6718 originates from exons 2 to 4 of the CWF19L1 gene (266 nt) (Figure S1A, Supporting Information). We validated that circ6718 possesses a circular structure, exhibiting enhanced stability compared to its parental gene, which is resistant to RNase R digestion (Figure S1B,C, Supporting Information). The levels of sEVs‐circ6718 were significantly elevated in GC patients when compared to healthy individuals and those with chronic atrophic gastritis (Figure 1C). Expression levels increased with advancing clinical stage (Figure 1D) and decreased postoperatively (Figure 1E), showing correlation with distal metastasis (Table S1, Supporting Information) and poor patient survival (Figure 1F). Receiver operating characteristic (ROC) analysis demonstrated its diagnostic potential, with an area under the curve (AUC) ranging from 0.9 to 0.96 (Figure 1G,H). The presence of serum sEVs from GC patients, chronic atrophic gastritis cases, and healthy controls was validated using transmission electron microscopy (TEM), nanoparticle tracking analysis (NTA), and Western blot (Figure 1I–K). Subsequently, we collected and characterized sEVs from the supernatant of various GC cell lines, finding that the expression of circ6718 in the sEVs of these GC cells was higher than that in sEVs derived from GES‐1 cells (Figure 1L–O). These findings establish sEVs‐circ6718 as a promising non‐invasive biomarker for GC.

### 2.2 sEV‐Delivered Circ6718 Drives Gastric Cancer Progression

**FIGURE 2:**
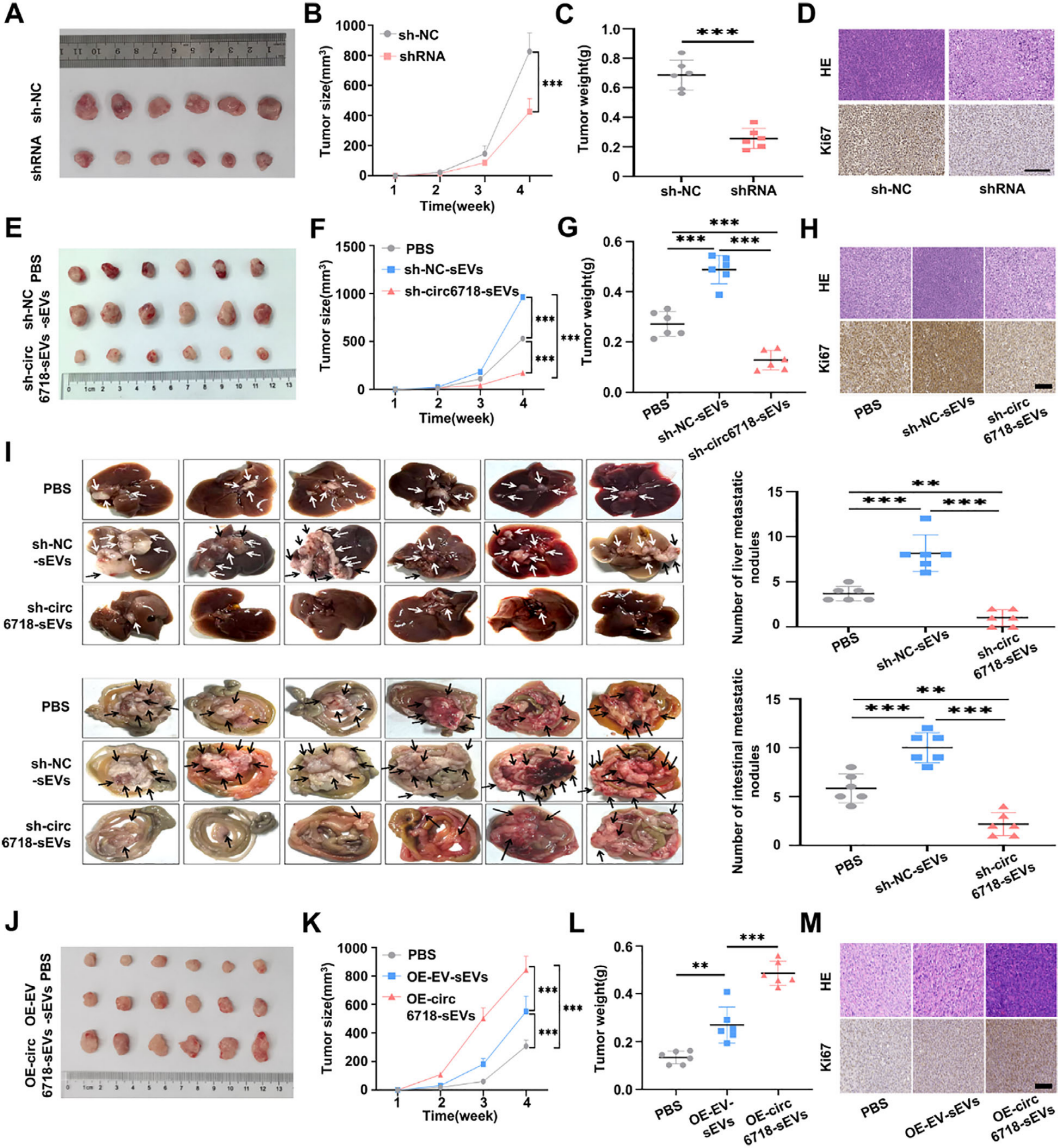
sEVs‐circ6718 promotes GC progression in vivo. (A) Volume measurements of subcutaneous xenograft tumors in mice injected with control versus circ6718 shRNA GC cells (n = 6 mice/group). (B) Size of subcutaneous xenograft tumors in mice injected with control or circ6718 shRNA GC cells (n = 6 mice/group). (C) Weight of subcutaneous xenograft tumors in mice injected with control versus circ6718 shRNA GC cells (n = 6 mice/group). (D) HE staining and Ki‐67 immunohistochemical staining of xenograft tumors from control and circ6718 shRNA mice (scale bar = 50 µm). (E) Volume of subcutaneous xenograft tumors in mice injected with PBS, control sEVs, and circ6718 knockdown sEVs via tail vein injection (n = 6 mice/group). (F) Size of subcutaneous xenograft tumors in mice injected with PBS, control sEVs, and circ6718 knockdown sEVs via tail vein injection (n = 6 mice/group). (G) Weight of subcutaneous xenograft tumors in mice injected with PBS, control sEVs, and circ6718 knockdown sEVs via tail vein injection (n = 6 mice/group). (H) HE staining and Ki‐67 immunohistochemical staining of xenograft tumors from mice injected with PBS, control sEVs, and circ6718 knockdown sEVs via tail vein injection (scale bar = 50 µm). (I) Number of liver and intestinal metastatic tumor nodules in the abdominal metastatic tumor model in mice injected with PBS, control sEVs, and circ6718 knockdown sEVs via tail vein injection (n = 6 mice/group). (J) Volumes of subcutaneous xenograft tumors in mice injected via the tail vein with PBS, control sEVs, or circ6718‐overexpressing sEVs (n = 6 mice/group). (K) Size of subcutaneous xenograft tumors in mice injected with PBS, control sEVs, and circ6718‐overexpressing sEVs via tail vein injection (n = 6 mice/group). (L) Weights of subcutaneous xenograft tumors in mice injected with PBS, control sEVs, or circ6718‐ overexpressing sEVs via tail vein injection (n = 6 mice/group). (M) HE staining and Ki‐67 immunohistochemical staining of xenograft tumors from mice injected via the tail vein with PBS, control sEVs, and circ6718 overexpressing sEVs in (scale bar = 50 µm). The data were plotted as Mean ± SEM. Statistical significance is indicated as follows: **p < 0.01, ***p < 0.001 by Student's *t*‐test for B and C; by one‐way ANOVA for F, G, I, K and L.

Circ6718 was significantly upregulated in GC cell lines (Figure S2A, Supporting Information). The expression levels of circ6718 in GC cells and their sEVs exhibit a positive correlation (Figure S2B). The knockdown and overexpression efficiency of circ6718 were verified (Figure S2C,D, Supporting Information). Functional studies demonstrated that the knockdown of circ6718 suppressed GC cells proliferation, migration, invasion, and epithelial‐mesenchymal transition (EMT) while promoting apoptosis, evidenced by Western blot results. Conversely, overexpression of circ6718 had the opposite effect (Figure S2E–M, Supporting Information). In xenograft models, circ6718 knockdown markedly reduced tumor growth, the number of Ki67^+^ cells, and tumor weight (Figure 2A–D), confirming its oncogenic role. In a mouse subcutaneous xenograft tumor model, the tumor size in the circ6718 knockdown sEVs (sh‐circ6718‐sEVs) group was significantly smaller and grew more slowly compared to the control sEVs (sh‐NC‐sEVs) group (Figure 2E–G). Immunohistochemistry analysis showed a decreased proportion of Ki67‐positive proliferating cells in the sh‐circ6718‐sEVs group (Figure 2H). Furthermore, metastatic nodules in the liver and intestinal tissues of tumor‐bearing mice were significantly reduced in the sh‐circ6718‐sEVs group compared to the sh‐NC‐sEVs group (Figure 2I). Conversely, sEVs carrying circ6718 overexpression constructs (OE‐circ6718‐sEVs) accelerated tumor progression. (Figure 2J–M). Collectively, these results suggest that circ6718 exerts promotive effects on GC progression both in vitro and in vivo.

### 2.3 Circ6718 Promotes Gastric Cancer Progression by Acting as a ceRNA Sponge to Sequester hsa‐miR‐561‐3p

**FIGURE 3:**
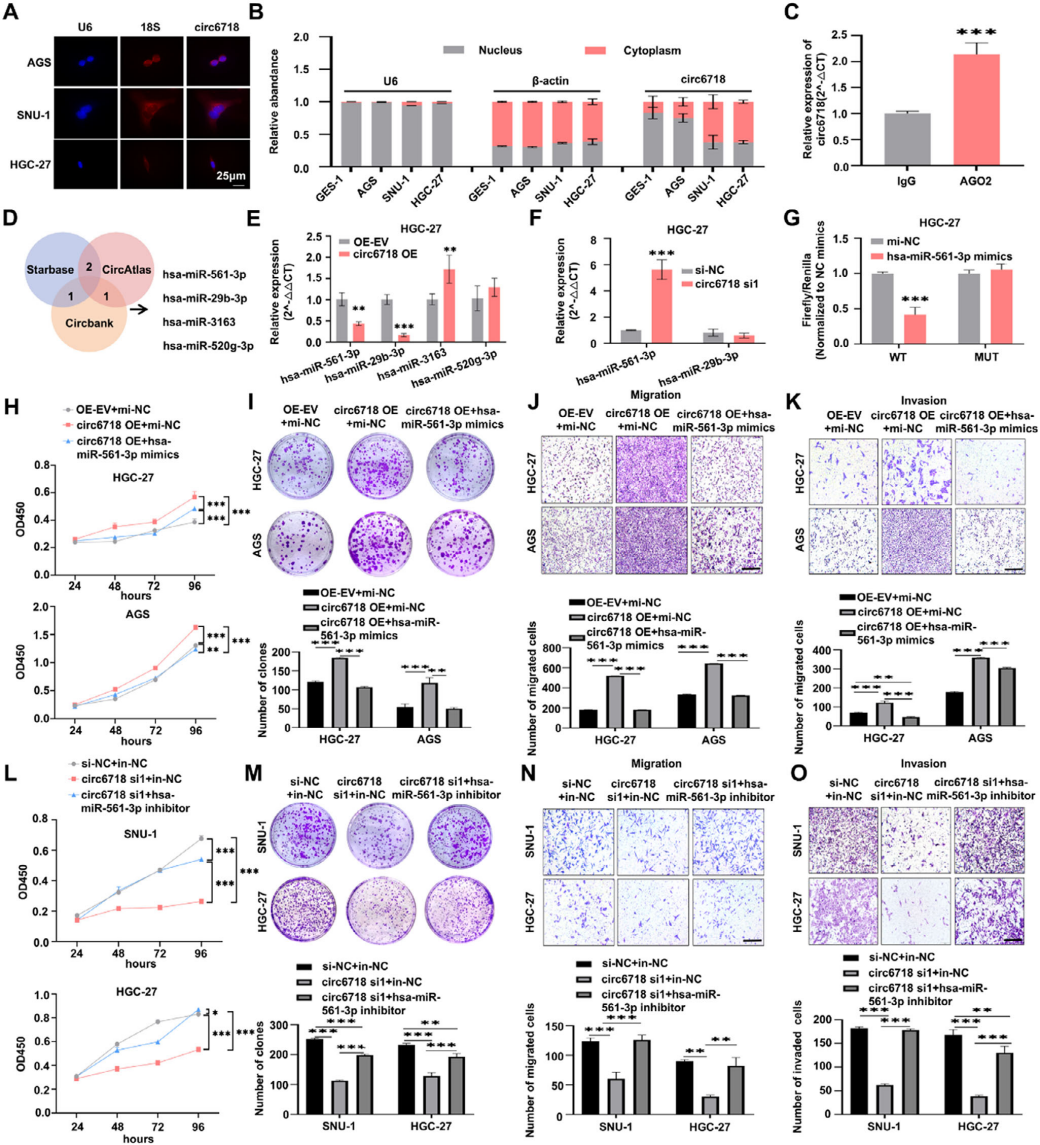
Circ6718 sponges hsa‐miR‐561‐3p to enhance oncogenic signaling. (A) RNA fluorescence in situ hybridization (FISH) for circ6718 (scale bar = 25 µm). (B) Cellular RNA fractionation analysis (n = 3). (C) RNA immunoprecipitation assay demonstrating the binding of circ6718 to Ago2 protein (n = 3). (D) Potential miRNA interactions with circ6718 were analyzed using various databases. (E) Expression levels of miRNAs after transfection with circ6718 OE were detected by qRT‐PCR (n = 3). (F) qRT‐PCR was employed to assess detect miRNA expression after transfection with circ6718 siRNA1 (n = 3). (G) Relative luciferase activity of wild‐type and mutant circ6718 constructs co‐transfected with hsa‐miR‐561‐3p mimics or a miRNA negative control (n = 3). The CCK‐8 assay (H), colony formation (I), Transwell migration (J), and matrigel invasion assay (K) were conducted in control, circ6718 overexpressing, and circ6718‐overexpressing GC cells co‐transfected with hsa‐miR‐561‐3p mimics (scale bar = 200 µm) (n = 3). The CCK‐8 assay (L), colony formation assay (M), Transwell migration assay (N), and matrigel invasion assay (O) were performed on GC cells co‐transfected with circ6718 siRNA and hsa‐miR‐561‐3p inhibitor (scale bar = 200 µm) (n = 3). Data were expressed as Mean ± SEM. Statistical significance is indicated as follows: *p < 0.05, **p < 0.01, ***p < 0.001 by Student's *t*‐test for C; by one‐way ANOVA for E‐O.

RNA FISH and subcellular fractionation assays confirmed that circ6718 predominantly localized in the cytoplasm (Figure 3A,B), suggesting a role in post‐transcriptional regulation. The RIP assay demonstrated its association with Ago2, supporting its function as a miRNA sponge (Figure 3C). Among the predicted miRNAs (Figure 3D), only hsa‐miR‐561‐3p exhibited an inverse correlation with circ6718 (Figure 3E,F), consistent with the anticipated regulatory mechanism. Furthermore, circ6718 expression was down‐regulated in various GC cell lines (Figure S3A,B, Supporting Information). Luciferase reporter assays validated that in GC cells transfected with the wild‐type (WT) circ6718 vector, co‐transfection with hsa‐miR‐561‐3p mimics resulted in a significant reduction in luciferase activity compared to the negative control (mi‐NC). Conversely, in GC cells transfected with the mutant (MUT) circ6718 vector, co‐transfection with hsa‐miR‐561‐3p mimics exhibited no significant alteration in luciferase activity. These findings indicate that the regulatory effect of hsa‐miR‐561‐3p on circ6718 depends on its predicted specific binding sites. Circ6718 may function as a competing endogenous RNA (ceRNA) by sequestering hsa‐miR‐561‐3p (Figure 3G; Figure S3C, Supporting Information). Quantitative RT‐PCR (qRT‐PCR) validated the transfection efficiency of hsa‐miR‐561‐3p mimics in GC cells (Figure S3D, Supporting Information). We found that hsa‐miR‐561‐3p mimics inhibit the proliferation and metastasis of GC cells, while the hsa‐miR‐561‐3p inhibitor had the opposite effect (Figure S3E–L, Supporting Information). To explore the potential interaction between circ6718 and hsa‐miR‐561‐3p, we co‐transfected a circ6718 overexpression plasmid with hsa‐miR‐561‐3p mimics. Notably, hsa‐miR‐561‐3p mimics partially reversed the pro‐proliferative and metastatic effects of circ6718 on GC cells (Figure 3H–K). Furthermore, co‐transfection of circ6718 siRNA with the hsa‐miR‐561‐3p inhibitor resulted in a partial reversal of the suppressive effects of circ6718 siRNA on GC cell growth, migration, and invasion (Figure 3L–O).

### 2.4 Circ6718/hsa‐miR‐561‐3p Promotes Gastric Cancer Progression by Activating SAAL1/PRRX1/TGFβ1 Signaling

**FIGURE 4:**
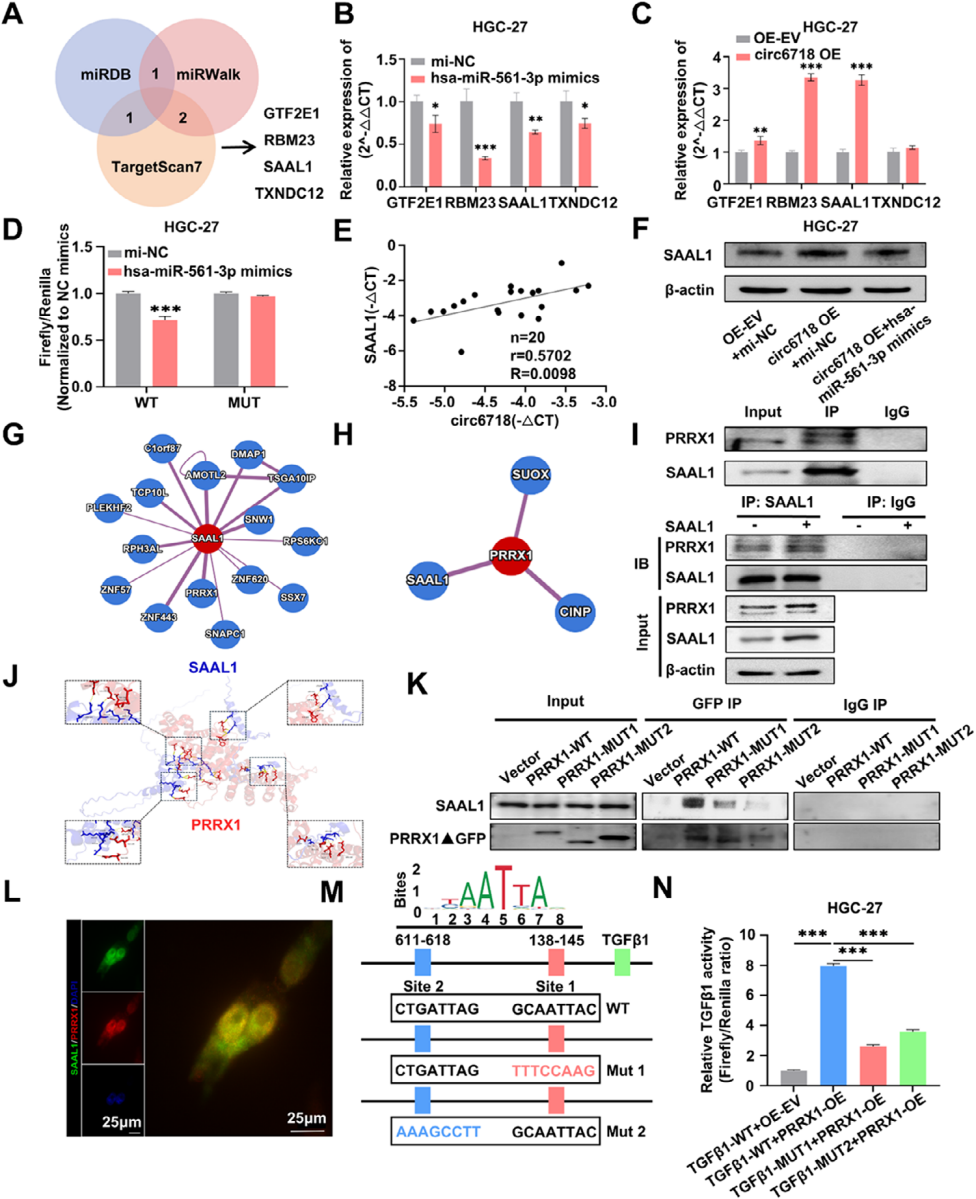
Circ6718/hsa‐miR‐561‐3p exerts pro‐oncogenic effects via targeting SAAL1/PRRX1. (A) Potential target genes that may interact with hsa‐miR‐561‐3p were analyzed using various databases. (B) qRT‐PCR was performed to detect the expression of each target gene following transfection with hsa‐miR‐561‐3p mimics (n = 3). (C) qRT‐PCR was performed to detect the expression of each target gene after transfection with circ6718 overexpression (n = 3). (D) Relative luciferase activity of wild‐type and mutant SAAL1 constructs co‐transfected with hsa‐miR‐561‐3p mimics or a miRNA negative control (n = 3). (E) Serum sEVs samples from GC patients were analyzed to investigate for the correlation between circ6718 and SAAL1 expression (n = 20). (F) Western blot analysis of SAAL1 expression after transfection with circ6718 and hsa‐miR‐561‐3p mimics. (G,H) The HURI database predicted a possible interaction between SAAL1 and PRRX1. (I) Co‐IP experiments confirmed the binding of SAAL1 and PRRX1. (J) Structural predictions using Alphafold and Pymol software indicated a binding interaction between SAAL1 and PRRX1, with the yellow stick structure representing the hydrogen bonding site. (K) Co‐IP assay to detect the binding of WT and MUT PRRX1 to SAAL1in GC cells. L, IF experiments validated the interaction between SAAL1 and PRRX1 (scale bar = 25 µm). (M) The JASPAR database predicted possible binding sites of PRRX1 within TGFβ1 promoter region, and TGFβ1 promoter WT and MUT vectors were constructed. (N) A dual luciferase reporter gene assay detected the binding of PRRX1 to the TGFβ1 promoter region (n = 3). The data were plotted as Mean ± SEM. Statistical significance is indicated as follows: *p < 0.05, **p < 0.01, ***p < 0.001 by Student's *t*‐test for B and C; by one‐way ANOVA for B, C, D and N; by Spearman Pearson correlation analysis for E.

To identify the targets of hsa‐miR‐561‐3p, we employed several bioinformatics tools including miRDB, miRWalk and Targetscan7 to select four candidate target genes for validation: GTF2E1, RBM23, SAAL1 and TXNDC12 (Figure 4A). Among these, only the expression of SAAL1 exhibited the expected changes following various transfection treatments. Notably, SAAL1 expression was found to be up‐regulated in the tissues of gastric cancer patients, suggesting that it may be a target gene regulated downstream of circ6718/hsa‐miR‐561‐3p (Figure 4B,C; Figure S4A–C, Supporting Information). Luciferase reporter assays confirmed direct binding (Figure 4D; Figure S4D, Supporting Information). Furthermore, correlation analyses conducted on serum sEVs from 20 GC patients revealed a positive correlation between circ6718 and SAAL1 expression (Figure 4E). Western blot further validated the regulatory role of circ6718/hsa‐miR‐561‐3p on SAAL1 (Figure 4F; Figure S4E,F, Supporting Information). To investigate SAAL1's binding to PRRX1 and its role in activating the TGFβ1/Smad2/3 signaling pathway, we performed experiments to predict the target proteins and regulatory mechanisms associated with the SAAL1 ensemble by analyzing protein‐protein interactions (Figure 4G,H). Co‐immunoprecipitation (Co‐IP) experiments demonstrated that SAAL1 can bind to PRRX1. Furthermore, transfection with an overexpression plasmid for SAAL1 enhanced the interaction between these two proteins (Figure 4I). To predict the interaction sites of SAAL1 and PRRX1, we utilized AlphaFold and PyMOL software, which revealed hydrogen‐bonding sites between the two proteins, indicated by yellow dotted lines (Figure 4J). Our analysis showed that most predicted hydrogen bond binding sites were located within two structural domains of PRRX1 (residues 63–103, 99–151). Based on these findings, we designed wild‐type (PRRX1‐WT) and mutant vectors (PRRX1‐MUT1 and PRRX1‐MUT2) targeting these domains to assess their role in the binding of SAAL1 and PRRX1. The results indicated that the amino acid sequence from residues 99 to 151 in the PRRX1 domain (PRRX1‐MUT2) is critical for the interaction between SAAL1 and PRRX1 (Figure 4K). Immunofluorescence (IF) experiments further confirmed the binding of SAAL1 and PRRX1 (Figure 4L). Subsequently, we retrieved the TGFβ1 promoter sequence from the UCSC database and employed the JASPAR database to predict the binding sites of PRRX1 within the TGFβ1 promoter region (Figure S4G,H, Supporting Information). The top two predicted binding sites were selected for mutation, and we constructed wild‐type plasmids (TGFβ1‐WT) and mutant plasmids (TGFβ1‐MUT1 and TGFβ1‐MUT2) for the TGFβ1 promoter region (Figure 4M). Dual luciferase reporter gene assays revealed that PRRX1 enhanced the transcriptional activation of TGFβ1, while the transcriptional activity of TGFβ1 was significantly downregulated following transfection with the two mutant vectors (Figure 4N).

**FIGURE 5:**
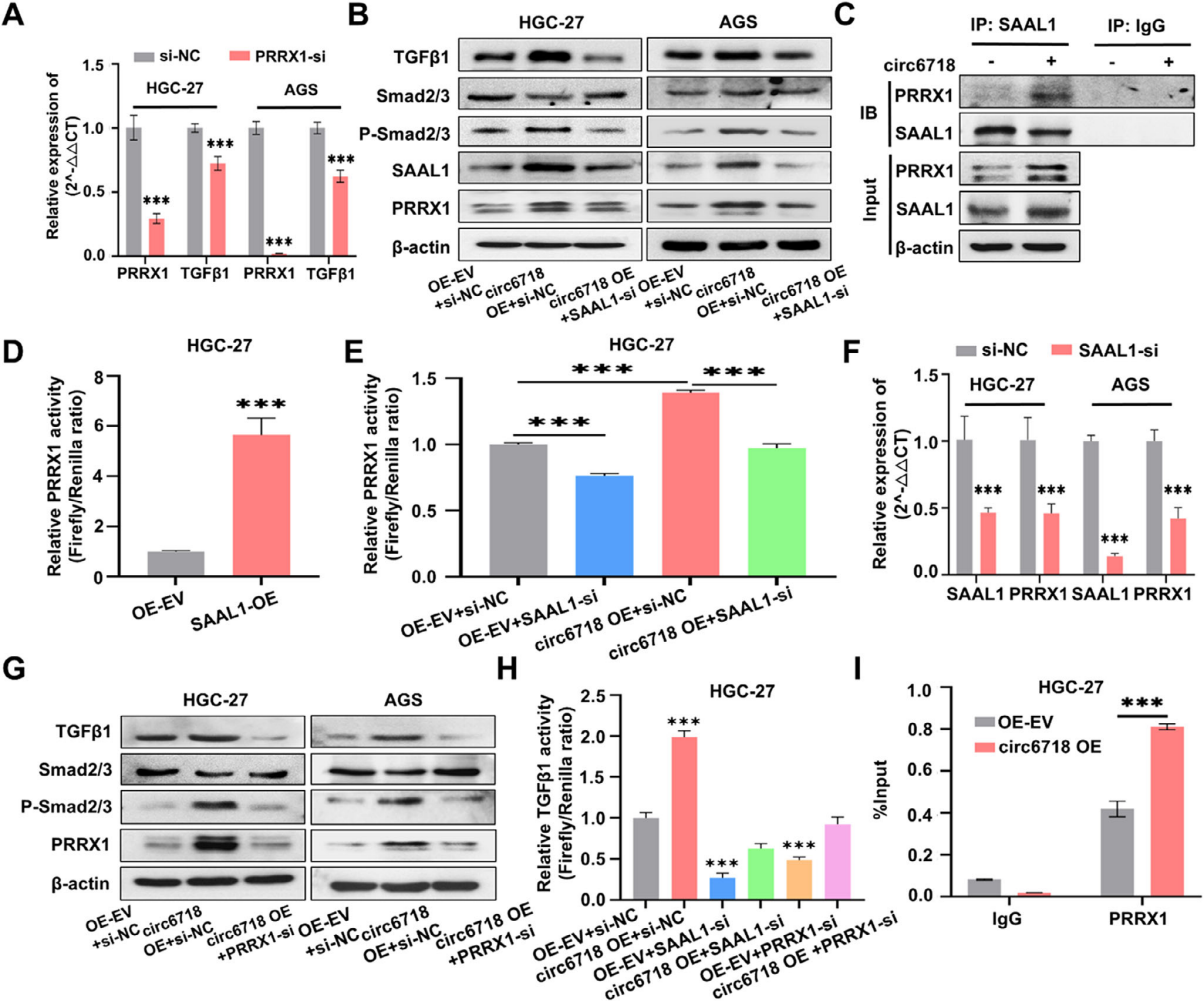
Circ6718 activates the TGFβ1/Smad2/3 signaling pathway via SAAL1/PRRX1. (A) qRT‐PCR was used to evaluate TGFβ1 expression following transfection with PRRX1 siRNA (n = 3). (B) Western blot analysis revealed alterations in the PRRX1/TGFβ1/Smad2/3 signaling pathway following co‐transfection with circ6718 overexpression and SAAL1 siRNA. (C) Co‐IP experiments indicated that circ6718 overexpression enhances the binding affinity of SAAL1 to PRRX1 in HGC‐27 cells. (D) The transcriptional activity of PRRX1, after SAAL1 overexpression, was evaluated using a dual luciferase reporter gene assay (n = 3). (E) A dual luciferase reporter gene assay also measured PRRX1 transcriptional activity after transfection with circ6718 overexpression and SAAL1 siRNA (n = 3). (F) qRT‐PCR analysis was conducted to quantify PRRX1 expression levels following SAAL1 siRNA transfection (n = 3). (G) Western blot analysis revealed changes in the TGFβ1/Smad2/3 signaling pathway alteration after co‐transfection with circ6718 overexpression and PRRX1 siRNA. (H) A dual luciferase reporter gene assay assessed TGFβ1 transcriptional activity following co‐transfection with circ6718 overexpression and SAAL1 siRNA or PRRX1 siRNA (n = 3). (I) ChIP assays detected the binding of PRRX1 to the TGFβ1 promoter after circ6718 overexpression (n = 3). The data were plotted as Mean ± SEM. Statistical significance is indicated as follows: ***p < 0.001 by Student's t‐test for D; by one‐way ANOVA for A, E, F, H, and I.

We transfected PRRX1 siRNA into GC cells and observed a significant decrease in TGFβ1 expression (Figure 5A). Subsequently, we introduced circ6718 overexpression and SAAL1 siRNA into GC cells. The results indicated that SAAL1 siRNA effectively reduced cell proliferation, colony formation, migration and invasion (Figure S5A–D, Supporting Information). Western blot assays demonstrated that circ6718 overexpression actives the TGFβ1/Smad2/3 signaling pathway, and this activation was inhibited by co‐transfection with SAAL1 siRNA (Figure 5B). Furthermore, SAAL1 siRNA suppressed the activation of the TGFβ1/Smad2/3 signaling pathway (Figure S5E, Supporting Information). Co‐IP assays revealed that the overexpression of circ6718 enhances the binding of SAAL1 to PRRX1 (Figure 5C). Additionally, we constructed a promoter plasmid for PRRX1 and performed a dual luciferase reporter gene assay, which demonstrated that overexpression of both circ6718 and SAAL1 increased the transcriptional activity of PRRX1, while SAAL1 siRNA exhibited the opposite effect (Figure 5D,E). Following the transfection of SAAL1 siRNA into GC cells, we observed a downregulation of PRRX1 expression (Figure 5F). Notably, transfection with PRRX1 siRNA partially reversed the enhanced cell proliferation, migration, and invasion, as well as the activation of the TGFβ1/Smad2/3 signaling pathway induced by circ6718 overexpression (Figure 5G; Figure S6A–E, Supporting Information). Moreover, knockdown of PRRX1 inhibited activation of the TGFβ1/Smad2/3 signaling pathway (Figure S6F, Supporting Information). After transfecting the SAAL1 overexpression plasmid into GC cells, we observed an upregulation of PRRX1 expression (Figure S7A, Supporting Information). Moreover, the overexpression of SAAL1 partially reversed the reduction in cell proliferation, migration, and invasion, as well as the inhibition of the TGFβ1/Smad2/3 signaling pathway induced by circ6718 siRNA (Figure S7B–E, Supporting Information). Additionally, the overexpression of SAAL1 enhanced the activation of the TGFβ1/Smad2/3 signaling pathway (Figure S7F, Supporting Information). Overexpression of PRRX1 in GC cells increased TGFβ1 expression (Figure S8A, Supporting Information). Furthermore, transfection with the PRRX1 overexpression plasmid produced effects similar to those observed with SAAL1 overexpression on the biological functions of GC cells (Figure S8B–E, Supporting Information). Finally, the overexpression of PRRX1 promoted the activation of the TGFβ1/Smad2/3 signaling pathway (Figure S8F, Supporting Information). Additionally, dual luciferase reporter gene assays demonstrated that transfection with the circ6718 overexpression plasmid promoted the transcriptional activation of TGFβ1, while co‐transfection with SAAL1 or PRRX1 siRNA effectively inhibited this activation (Figure 5H). Chromatin immunoprecipitation (ChIP) experiments showed that PRRX1 binds to the promoter region of TGFβ1, and that circ6718 overexpression promotes the binding between the two (Figure 5I). Furthermore, we examined the expression of target proteins in tumor tissues from a subcutaneous tumor model in nude mice. The results revealed a decrease in both SAAL1 and PRRX1 expression, along with reduced activation of the TGFβ1/Smad2/3 signaling pathway in the tumor tissues of the circ6718 knockdown group (Figure S9A,B, Supporting Information).

### 2.5 sEVs‐circ6718 Drives GC‐MSC‐ to‐ CAF Differentiation

To investigate how sEVs‐circ6718 promotes tumor progression by driving the differentiation of GC‐MSCs into CAFs, we isolated tumor tissue‐derived GC‐MSCs from GC patients with T2N2M0 staging. After 7 to 14 days of primary culture, the cells exhibited a fibroblast‐like morphology with the potential for osteogenic and lipogenic differentiation. They were positive for CD44 and CD73, while negative for CD45, CD14 and CD34 (Figure S10A–D, Supporting Information).

**FIGURE 6:**
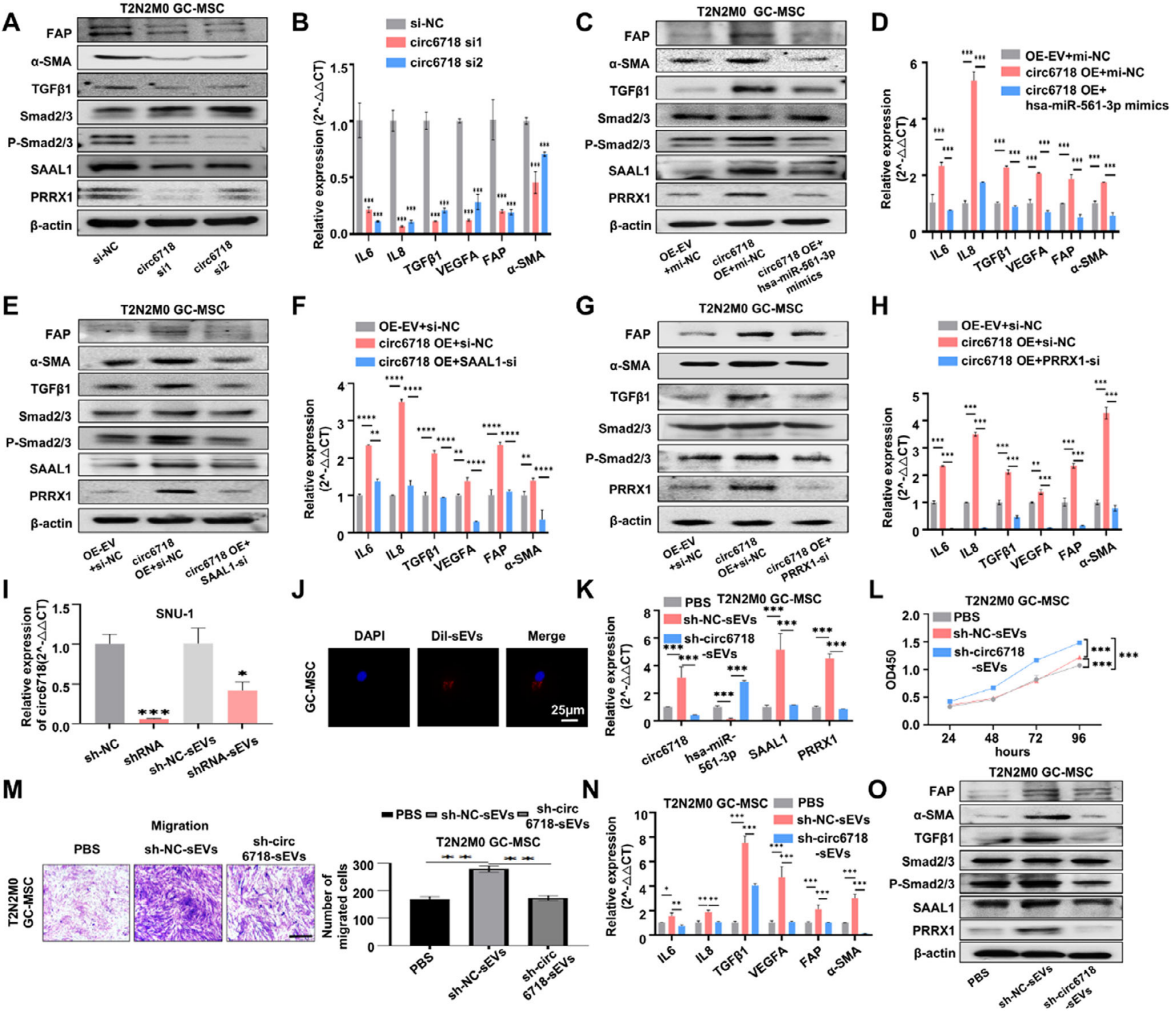
sh‐circ6718‐sEVs inhibit the transdifferentiation of GC‐MSCs. (A) Western blot analysis of CAF marker expression following GC‐MSC transfection with circ6718 siRNA. (B) Expression levels of CAF markers and cytokines were quantified using qRT‐PCR after GC‐MSC transfection with circ6718 siRNA (n = 3). (C–H) Circ6718 regulates the activation of the TGFβ1/Smad2/3 signaling pathway by hsa‐miR‐561‐3p/SAAL1/PRRX1, thereby promoting GC‐MSC activation and enhancing the expression of CAF markers and cytokine release (n = 3). (I) qRT‐PCR analysis of circ6718 expression in GC cells with stable circ6718 knockdown and sEVs derived from GC cells (n = 3). (J) IF staining was used to detect the uptake of sEVs from GC cells with circ6718 knockdown by GC‐MSC (scale bar = 25 µm). (K) qRT‐PCR detection of gene expression in GC‐MSC after treatment with sEVs from GC cells with circ6718 knockdown (n = 3). (L) CCK8 assay was performed to evaluate the proliferation ability of GC‐MSC following sEVs treatment from GC cells with circ6718 knockdown (n = 3). (M) Transwell migration assay assessed the migratory ability of GC‐MSC after sEVs treatment from GC cells with circ6718 knockdown (scale bar = 200 µm) (n = 3). N, Expression levels of CAF markers and cytokines in GC‐MSCs treated sEVs from GC cells with circ6718 knockdown were quantified using qRT‐PCR (n = 3). (O) Western blot analysis was employed to assess CAF marker expression in GC‐MSCs after sEVs treatment from GC cells with circ6718 knockdown. The data were plotted as Mean ± SEM. Statistical significance is indicated as follows: *p < 0.05, **p < 0.01, ***p < 0.001 by one‐way ANOVA for B, D, F, H, I, K, L, M and N.

We performed knock down and overexpression of circ6718 in GC‐MSCs (Figure S11A, Supporting Information). Transfection with circ6718 siRNA suppressed the expression of CAF markers and pro‐inflammatory factors in GC‐MSCs (Figure 6A,B). Co‐transfection of circ6718 overexpression along with hsa‐miR‐561‐3p mimics, SAAL1 siRNA or PRRX1 siRNA partially attenuated the increased expression of CAF markers and cytokines in GC‐MSCs induced by circ6718 (Figure 6C–H). Dual luciferase reporter gene assays demonstrated that hsa‐miR‐561‐3p could bind to both circ6718 and SAAL1 in GC‐MSCs (Figure S11B,C, Supporting Information).

Given the high expression of circ6718 in sEVs derived from SNU‐1 cells, we transfected lentiviral knockdown vectors into SNU‐1, selected cells with stable circ6718 knockdown after puromycin screening, and subsequently collected the sEVs (Figure S12A, Supporting Information). The qRT‐PCR results confirmed the successful isolation of sEVs with low circ6718 expression from GC cells (Figure 6I). IF results demonstrated that GC‐MSCs were able to uptake sEVs from GC cells (Figure 6J). sEVs with circ6718 knockdown decreased the expression of circ6718, SAAL1 and PRRX1, while increasing the expression of hsa‐miR‐561‐3p in GC‐MSCs compared to control sEVs (Figure 6K). Moreover, these sEVs inhibited the proliferation, metastatic ability, and expression of cytokines and CAF markers in GC‐MSCs (Figure 6L–O). Additionally, IF experiments demonstrated that sh‐circ6718‐sEVs could also be taken up by GC cells, thereby inhibiting their proliferation, migration and invasion (Figure S12B–H, Supporting Information). We assessed the expression levels of circ6718 and related proteins in a nude mouse subcutaneous tumor model. The qRT‐PCR results confirmed that circ6718 expression was lowest in the sh‐circ6718‐sEVs group (Figure S13A, Supporting Information). Immunohistochemistry and Western blot analysis revealed a decreased proportion of SAAL1 and PRRX1 positive proliferating cells in the sh‐circ6718‐sEVs group (Figure S13B,C, Supporting Information). Furthermore, we analyzed the expression levels of relevant proteins in abdominal metastatic tumor tissue samples from nude mice. The results from immunohistochemistry and Western blot also indicated a reduced proportion of N‐cadherin and Vimentin‐positive proliferating cells, alongside an increased proportion of E‐cadherin‐positive proliferating cells in the sh‐circ6718‐sEVs group (Figure S13D,E, Supporting Information).

**FIGURE 7:**
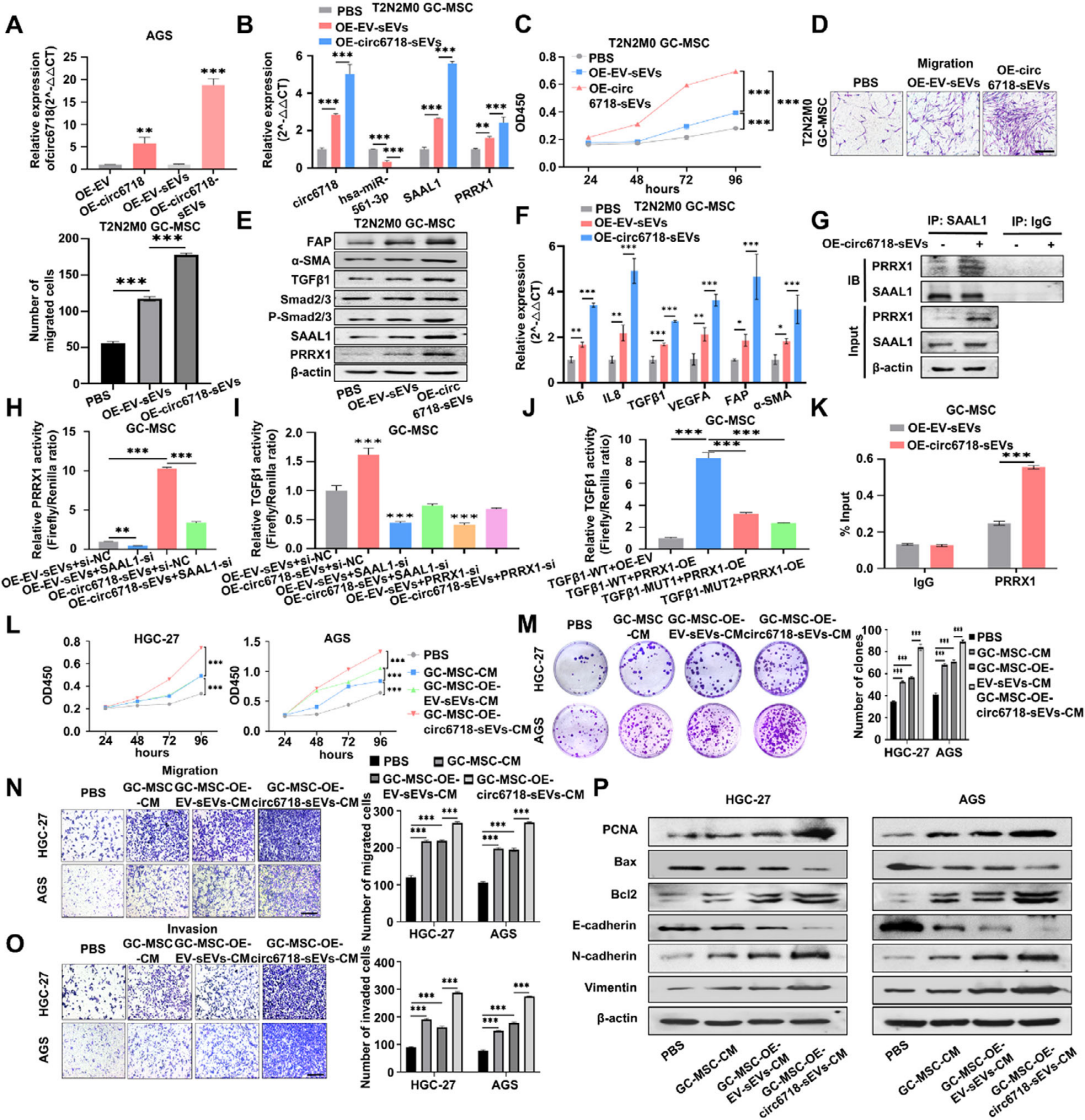
OE‐circ6718‐sEVs promote GC‐MSCs transdifferentiation. (A) qRT‐PCR was employed to assess the expression levels of circ6718 in gastric cancer cells with engineered for stable overexpressing of circ6718, as well as in sEVs derived from these cells (n = 3). (B) qRT‐PCR analysis of gene expression in GC‐MSC following treatment with sEVs from GC cells overexpressing circ6718 (n = 3). (C) CCK8 assay assessed the proliferative ability of GC‐MSC after treatment with sEVs from GC cells with circ6718 overexpression (n = 3). (D) Transwell migration assay evaluated the migratory ability of GC‐MSC after treatment with sEVs from GC cells overexpressing circ6718 (scale bar = 200 µm) (n = 3). (E) Western blot analysis determined the expression levels of CAF markers in GC‐MSCs treated with sEVs from circ6718‐overexpressing GC cells. (F) qRT‐PCR analysis was conducted to evaluate CAF markers and cytokines in GC‐MSCs treated with sEVs from GC cells overexpressing circ6718 (n = 3). (G) Co‐IP experiments demonstrated that circ6718 overexpression in sEVs enhanced the binding of SAAL1 to PRRX1 in GC‐MSCs. (H) A dual luciferase reporter gene assay was performed to detect the transcriptional activity of PRRX1 after treatment with circ6718 overexpressing sEVs and transfection with SAAL1 siRNA (n = 3). (I) Another dual luciferase reporter gene assay was performed to examined TGFβ1 transcriptional activity after treatment with circ6718‐overexpression sEVs and co‐transfection with either SAAL1 siRNA or PRRX1 siRNA (n = 3). (J) A dual luciferase reporter gene assay demonstrated the binding of PRRX1 to the TGFβ1 promoter region in GC‐MSC (n = 3). (K) ChIP analysis revealed that PRRX1 binds to the TGFβ1 promoter following treatment with circ6718‐ overexpressing sEVs (n = 3). The CCK‐8 assay (L), colony formation assay (M), Transwell migration assay (N), and matrigel invasion assay (O) were performed to evaluate the effects of activated GC‐MSCs, following OE‐circ6718‐sEVs treatment, on GC cells proliferation and metastasis (scale bar = 200 µm) (n = 3). (P) Western blot analysis was performed to evaluate the impacts of activated GC‐MSCs treated with OE‐circ6718‐sEVs on EMT markers and proliferation indices in GC. The data were plotted as Mean ± SEM. Statistical significance is indicated as follows: *p < 0.05, **p < 0.01, ***p < 0.001 by one‐way ANOVA for A, B, C, D, F, H, I, J, K, L, M, N, and O.

Given the low expression of circ6718 in sEVs derived from AGS cells, we transfected lentiviral overexpression vectors into AGS cells, resulting in stable AGS cells that overexpress circ6718 after puromycin screening. Subsequently, we isolated the sEVs (Figure 7A; Figure S14A, Supporting Information). IF results indicated that GC‐MSCs could internalize sEVs from GC cells (Figure S14B, Supporting Information). Compared to control sEVs, the sEVs overexpressing circ6718 exhibited increased expression levels of circ6718, SAAL1, and PRRX1, while decreasing the expression of hsa‐miR‐561‐3p in GC‐MSCs (Figure 7B). Overexpression of circ6718 in sEVs (OE‐circ6718‐sEVs) promoted proliferation, metastasis, and the expression of pro‐inflammatory factors and CAF markers in GC‐MSCs (Figure 7C–F). Co‐IP experiments revealed that the interaction between SAAL1 and PRRX1 was enhanced following treatment with OE‐circ6718‐sEVs in GC‐MSCs (Figure 7G). Immunofluorescence experiments further confirmed the co‐localization of SAAL1 and PRRX1 in GC‐MSCs (Figure S14C, Supporting Information). A dual luciferase reporter gene assay demonstrated that overexpression of SAAL1 in GC‐MSCs increased the transcriptional activity of PRRX1 (Figure S14D, Supporting Information). To validate the effect of the SAAL1‐PRRX1 interaction on TGFβ1, we assessed TGFβ1 transcriptional activity using a dual luciferase reporter assay. The results indicated that TGFβ1 transcriptional activity was downregulated following SAAL1 knockdown, with recovery observed upon co‐transfection with an overexpressed PRRX1 plasmid (Figure S14E). Western blot analysis showed that SAAL1 knockdown in GC‐MSCs led to reduced expression of CAF markers and diminished activation of the TGFβ1/Smad2/3 signaling pathway, which was restored upon co‐transfection with an overexpressed PRRX1 plasmid (Figure S14F). The treatment with OE‐circ6718‐sEVs significantly enhanced transcriptional activation of PRRX1, an effect that was diminished upon transfection with SAAL1 siRNA (Figure 7H). Furthermore, the application of OE‐circ6718‐sEVs also increased TGFβ1 transcriptional activity, which was inhibited by transfection with either SAAL1 or PRRX1 siRNA (Figure 7I). Additionally, the transfection with two TGFβ1 promoter mutant plasmids resulted in a marked a decrease in TGFβ1 transcriptional activity (Figure 7J). ChIP assays revealed that the treatment of GC‐MSCs with OE‐circ6718‐sEVs facilitated the binding of PRRX1 to the TGFβ1 promoter (Figure 7K).

To eliminate inaccuracies arising from individual variability, we concurrently isolated GC‐MSCs derived from tumor tissues of GC patients at T4aN3M0 clinical stage for experimentation. We treated GC‐MSCs with sh‐circ6718‐sEVs and OE‐circ6718‐sEVs, respectively. CCK8 assays, Transwell migration assays, and Western blot analyses demonstrated that sh‐circ6718‐sEVs inhibited GC‐MSC proliferation, migration capacity, and expression of CAF markers, while OE‐circ6718‐sEVs exerted the opposite effect (Figure S15A–C). Additionally, we isolated and extracted sEVs derived from GES‐1 cells (GES‐1 sEVs) to treat GC‐MSCs, finding that GC‐MSCs could also internalize the sEVs (Figure S15D). Compared to PBS or GES‐1 sEVs, sEVs derived from GC cells exhibited more pronounced effects on promoting proliferation, metastasis, and CAF marker expression in GC‐MSCs (Figure S15E–G). To validate the effects of sEVs on GC‐MSCs, we pretreated GC cells with the sEVs inhibitor GW4869 before co‐culturing them with GC‐MSCs. CCK8, Transwell migration assays, and Western blot results demonstrated that compared to PBS, the treated group exhibited significantly downregulated proliferation, migration, and CAF marker expression (Figure S15H–J).

Furthermore, experiments demonstrated that OE‐circ6718‐sEVs could be internalized by GC cells, leading to enhanced proliferation, migration and invasion (Figure S16A–G, Supporting Information). We assessed the expression levels of circ6718 and related proteins in a nude mouse subcutaneous tumor model. The qRT‐PCR results indicated the highest expression of circ6718 in the OE‐circ6718‐sEVs group (Figure S17A, Supporting Information). Additionally, immunohistochemistry and Western blot analyses revealed an increased proportion of SAAL1 and PRRX1‐positive proliferating cells within the OE‐circ6718‐sEVs group (Figure S17B,C, Supporting Information).

### 2.6 sEVs‐circ6718 Promotes Gastric Cancer Progression by Activating GC‐MSCs

To further confirm the significance of circ6718 in sEVs from GC cell and its role in transforming GC‐MSCs and CAFs in GC progression, we collected supernatants from GC‐MSCs and those treated with OE‐EV‐sEVs or OE‐circ6718‐sEVs after 48 h for in vitro experiments. The results showed that the GC progression was significantly promoted in the group treated with supernatants from both GC‐MSCs and GC‐MSCs treated with OE‐EV‐sEVs, compared to the PBS control group. Notably, the supernatant‐treated GC‐MSCs that received OE‐circ6718‐sEVs exhibited a more pronounced increase in the proliferation, migration, and invasion capabilities of GC cells (Figure 7L–P). We co‐cultured GC‐MSCs pretreated with sh‐circ6718‐sEVs with GC cells. The results demonstrated that, compared to the PBS group, sh‐NC‐sEVs‐pretreated GC‐MSCs enhanced the proliferation and metastatic abilities of GC cells, while sh‐circ6718‐sEVs exhibited the opposite effect (Figure S18A–D, Supporting Information). We also collected sEVs from sh‐circ6718‐sEVs‐pretreated GC‐MSCs to treat GC cells, resulting in similar findings (Figure S18E–H, Supporting Information). Furthermore, we collected sEVs from OE‐circ6718‐sEVs‐pretreated GC‐MSCs to treat GC cells, which significantly enhanced the proliferation and metastatic abilities of GC cells (Figure S18I–L, Supporting Information). Taken together, these findings demonstrate that circ6718‐mediated transformation of GC‐MSCs and CAFs by GC cell‐derived sEVs promotes the growth and metastasis of GC in vitro.

In addition, we established a subcutaneous nude mouse xenograft model by co‐injecting GC‐MSCs and GC cells, followed by tail vein injection of PBS or sEVs. The results demonstrated that tumor volume and weight were significantly reduced in the sh‐circ6718‐sEVs group, whereas the OE‐circ6718‐sEVs group exhibited the opposite effect (Figure S19A–C, Supporting Information). Furthermore, Masson and Sirius red staining along with immunohistochemical analyses, revealed that fibrosis levels and extracellular matrix components were markedly reduced in tumor tissues from the sh‐circ6718‐sEVs group, while the opposite was observed in the OE‐circ6718‐sEVs group (Figure S19D,E, Supporting Information).

Additionally, we collected serum‐derived sEVs from GC patients with relatively high expression of circ6718 and subsequently treated GC cells and GC‐MSCs with these vesicles. IF experiments indicated that both GC cells and GC‐MSCs were capable of internalizing serum sEVs from GC patients (Figure S20A, Supporting Information). Compared to the PBS group, treatment with serum sEVs from GC patients significantly enhanced the proliferation and metastasis of GC cells, as well as the transformation of GC‐MSCs and CAFs (Figure S20B–I, Supporting Information).

### 2.7 Circ6718 siRNA Engineered HEK293T sEVs Suppressed the Growth and Metastasis of Gastric Cancer

To explore the therapeutic potential of targeting circ6718, we developed HEK293T‐derived sEVs loaded with circ6718 siRNA (sEVs@circ6718 si) and investigated their effects on GC progression. The sEVs@circ6718 si were extracted and purified from HEK293T cell culture supernatant using differential centrifugation and characterized using NTA, TEM and Western blot (Figure S21A–C, Supporting Information). Subsequently, we treated GC cells with these sEVs and confirmed that circ6718 expression levels were significantly downregulated in GC cells following treatment with sEVs@circ6718 si treatment (Figure S21D, Supporting Information). IF assays confirmed that GC cells efficiently took up the engineered sEVs (Figure S21E, Supporting Information). Additionally, the proliferation, migration and invasion abilities of GC cells in the sEVs@circ6718 si‐treated group were significantly decreased compared to those in the PBS and sEVs@si NC groups (Figure S21F–J, Supporting Information).

**FIGURE 8:**
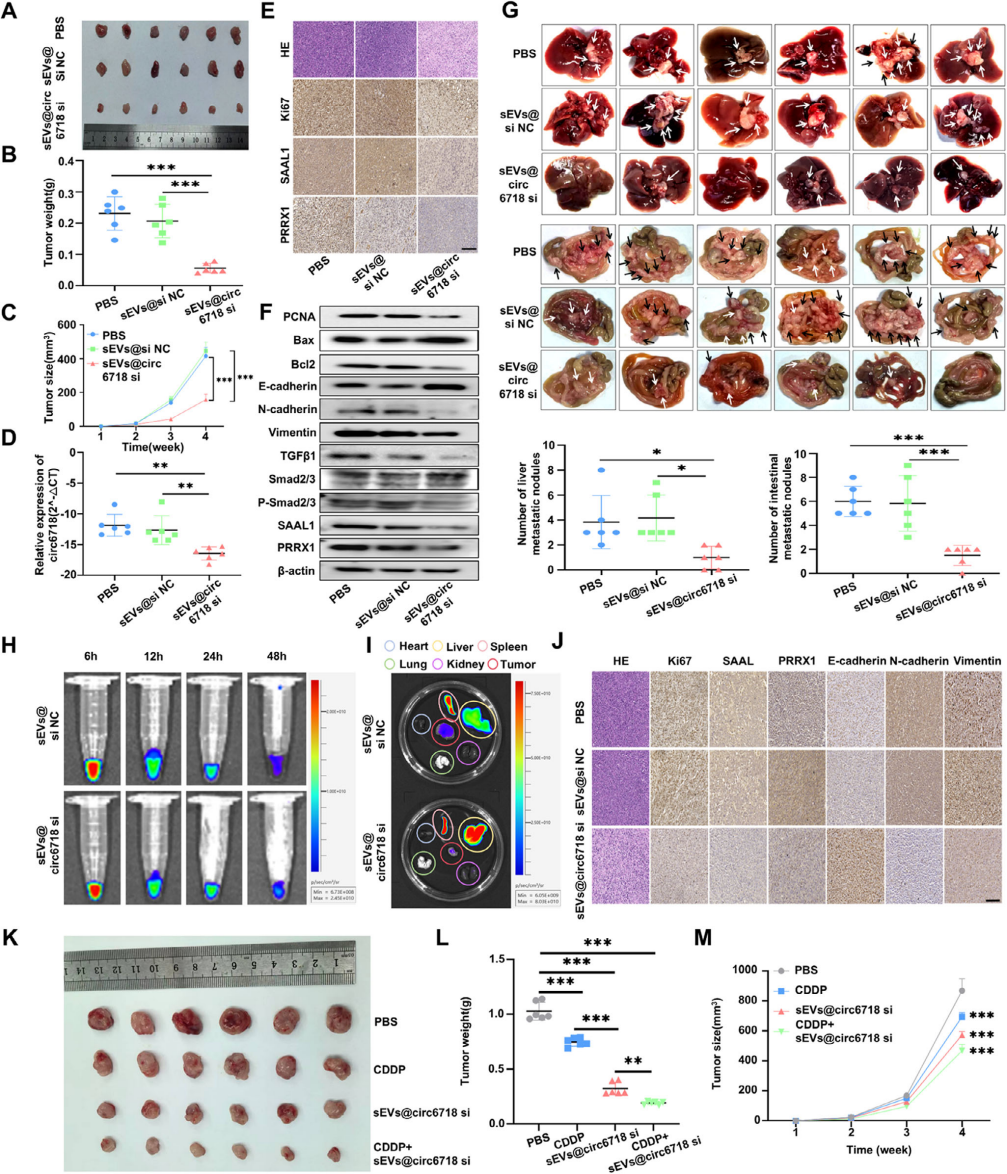
Circ6718 siRNA engineered sEVs inhibit GC progression in vivo. (A) Measurement of subcutaneous xenograft tumor volume in mice injected via the tail vein with PBS, control sEVs, or sEVs@circ6718 si (n = 6 mice/group). (B) Weights of subcutaneous xenograft tumors in mice injected with PBS, control sEVs, or sEVs@circ6718 si via the tail vein (n = 6 mice/group). (C) Tumor size comparison of subcutaneous xenograft s in mice injected with PBS, control sEVs, or sEVs@circ6718 si via the tail vein injection (n = 6 mice/group). (D) qRT‐PCR analysis of circ6718 expression in subcutaneous xenograft tumors from mice injected via the tail vein with PBS, control sEVs, or engineered sEVs (n = 6 mice/group). (E) HE staining and immunohistochemical staining of xenograft tumors from mice injected with PBS, control sEVs, and engineered sEVs (scale bar = 50 µm). (F) Western blot analysis of EMT and proliferation markers in xenograft tumors from mice treated with PBS, control sEVs, or engineered sEVs. (G) The number of liver and intestinal metastatic tumor nodules in the abdominal metastatic tumor model of mice injected via tail vein with PBS, control sEVs, or sEVs@circ6718 si (n = 6 mice/group). (H) Serum samples from mice with subcutaneous tumors following tail vein injection of sEVs@circ6718 si. (I) Following tail vein injection of sEVs@circ6718 si, the primary organs (heart, liver, lungs, spleen, kidneys, and tumor) of subcutaneous tumor‐bearing mice. J, HE staining and immunohistochemical staining of abdominal metastatic tumors in mice that were injected via the tail vein with PBS, control sEVs, or engineered sEVs (scale bar = 50 µm). (K) Measurement of subcutaneous xenograft tumor volume in mice following tail vein injection of PBS, cisplatin (CDDP), sEVs@circ6718 si, or CDDP+sEVs@circ6718 si (n = 6 mice/group). (L) The subcutaneous xenograft tumor volumes in mice injected via the caudal vein with PBS, CDDP, sEVs@circ6718 si, or CDDP+sEVs@circ6718 si (n = 6 mice/group). (M) Subcutaneous xenograft tumor weights in mice following tail vein injection of PBS, CDDP, sEVs@circ6718 si, or CDDP+sEVs@circ6718 si (n = 6 mice/group). The data were plotted as Mean ± SEM. Statistical significance is indicated as follows: *p < 0.05, **p < 0.01, ***p < 0.001 by one‐way ANOVA for B, C, D, G, L and M.

In a xenograft mouse model, the group treated with sEVs@circ6718 si demonstrated a significant reduction in both tumor weight and volume, along with down‐regulation of circ6718 levels in the tumor tissues, when compared to the PBS‐ and sEVs@si NC‐treated groups (Figure 8A–D). Results from immunohistochemistry and Western blot analyses indicated that the sEVs@circ6718 si‐treated group exhibited fewer Ki67, SAAL1, and PRRX1‐positive proliferating cells relative to the other two groups (Figure 8E,F). Furthermore, in an abdominal metastatic tumor model in nude mice, the sEVs@circ6718 si‐treated group showed a marked decrease in the number of liver and intestinal metastatic tumor nodules compared to the other two groups (Figure 8G). To evaluate the therapeutic efficacy of sEVs@circ6718 si, we extracted serum from nude mice at various time points for analysis. The results demonstrated that the fluorescence intensity of sEVs@circ6718 si remained detectable for up to 48 h (Figure 8H, I). Immunohistochemistry and Western blot assays indicated that the sEVs@circ6718 si‐treated group exhibited a decrease in the number of N‐cadherin‐ and Vimentin‐positive proliferating cells, alongside an increase in E‐cadherin‐positive proliferating cells (Figure 8J; Figure S22A, Supporting Information). Safety experiments revealed that sEVs@circ6718 si had no significant adverse effects on the major organs of nude mice, including the heart, liver, spleen, lungs and kidneys (Figure S22B, Supporting Information). Liver and kidney function analyses confirmed that sEVs@circ6718 si did not significant effects on liver and kidney function in nude mice (Figure S22C, Supporting Information). To further enhance the therapeutic efficacy of sEVs@circ6718 si, we administered combined therapy involving cisplatin and sEVs@circ6718 si. The results indicated a significant reduction in tumor volume and weight within the combined therapy group (Figure 8K–M). Masson and Sirius red staining, along with immunohistochemical results revealed markedly diminished fibrosis and extracellular matrix within the tumor tissue of the combined therapy group (Figure S22D,E, Supporting Information). In conclusion, these results suggest that sEVs@circ6718 si effectively suppressed the growth and metastasis of GC in vitro and in vivo, with no observed systemic toxicity.

## 3 Discussion

GC, a globally prevalent gastrointestinal malignancy, faces significant prognostic challenges primarily due to limitations in early detection. sEVs‐circRNAs have emerged as promising liquid biopsy target for cancer diagnosis. This study establishes serum sEVs‐circ6718 as a valuable biomarker for the early diagnosis and prognosis of GC. We confirmed the circular structure of circ6718 through RNase R resistance and oligo dT primer amplification, demonstrating its superior stability compared to linear RNAs. Notably, sEVs‐circ6718 achieved a sensitivity of 89.19% in distinguishing GC patients from healthy individuals, outperforming traditional biomarkers such as carcinoembryonic antigen (CEA) and carbohydrate antigen 242 (CA242), which exhibited a sensitivity of 86.25% as well as carbohydrate antigen 724 (CA724) and carbohydrate antigen19‐9 (CA19‐9), which had a sensitivity of 76.25% [29]. Its diagnostic AUC ranged from 0.9 to 0.96, exceeding that of sEVs‐circITCH (AUC = 0.7092), [30] sEVs‐hsa_circ_000200 (AUC = 0.7092), [31] and other biomarker combinations (AUC = 0.843) [32]. Elevated levels of sEVs‐circ6718 correlate with clinical stage, distant metastasis and poor survival rates, supporting its potential as a non‐invasive biomarker for early diagnosis and prognosis, as well as a therapeutic target.

A major innovation reveals circ6718 functions as a ceRNA for hsa‐miR‐561‐3p, thereby derepressing SAAL1 to activate PRRX1‐dependent transcription of TGFβ1. This cascade triggers the activation of the TGFβ1/Smad2/3 signaling pathway, which drives GC proliferation, metastasis, and the differentiation of GC‐MSCs into CAFs, a critical process central to stromal remodeling. To our knowledge, this is the first study to identify SAAL1 as an oncogenic target of hsa‐miR‐561‐3p in GC and to demonstrate the physical interaction between SAAL1 and PRRX1 via mutagenesis, localizing a critical binding domain to PRRX1 residues 99–151. As a transcription factor, PRRX1 has previously been implicated in EMT [33, 34]. This study shows that PRRX1 can bind to the promoter region of TGFβ1 in GC cells, thereby activating its transcription. This finding expands our understanding of non‐coding RNA regulation within the TME. Furthermore, we demonstrate that GC cell‐derived sEVs containing circ6718 are delivered to GC‐MSCs, promoting their activation and secretion of CAF markers and cytokines. Given that GC‐MSCs critically influence tumor growth, metastasis, drug resistance, immune escape, and angiogenesis, their activation is crucial for TME remodeling and the malignant progression of GC [10, 11, 35]. This represents the first report of sEVs‐circRNA‐mediated activation of GC‐MSCs, revealing a novel mechanism of tumor‐stroma crosstalk. Considering the role of CAFs in tumor progression, targeting circ6718 could disrupt stromal support and impede GC.

sEVs, characterized by their high biocompatibility and low immunogenicity, serve as ideal therapeutic vectors. Previous studies have demonstrated that sEVs‐delivering si‐ciRS‐122 can reverse oxaliplatin resistance in colorectal cancer [36]. and RGD‐exo‐circDIDO1 effectively inhibits GC [37]. In our research, we developed an innovative strategy utilizing HEK293T‐derived sEVs to deliver circ6718 siRNA (sEVs@circ6718 si) in combination with cisplatin therapy. To enhance the targeting efficiency of sEVs@circ6718 si, we designed the circ6718 siRNA to specifically target the unique antisplicing site of circ6718, thereby minimizing off‐target effects. This strategy effectively inhibited GC growth and metastasis both in vitro and in vivo, demonstrating favorable biosafety and suggesting dual targeting of tumor cells and stromal remodeling. Notably, several sEVs‐based therapies including dendritic cell sEVs vaccines (NCT01159288), chimeric sEVs vaccines (NCT05559177), and MSC‐exo‐KrasG12D siRNA (NCT03608631), Are Currently in Clinical Trials, underscoring the translational potential of our approach. Despite these promising findings, the Clinical Utility of circ6718 As a Diagnostic and Prognostic Biomarker Requires Validation in Larger and More Diverse Patient Cohorts Across Different Pathological stages. The functional Diversity of GC‐MSCs Within the TME Warrants Single‐Cell RNA Sequencing Analysis to Identify Specific Subpopulations That May Respond to Sevs circ6718. Furthermore, surface modifications such as CD47 for half‐life extension and iRGD/GE11peptides for tumor targeting, could enhance the delivery of sEVs. Combination approaches involving chemotherapy, TGFβ inhibitors, and immunotherapy may further improve therapeutic efficacy.

However, this study is subject to certain limitations. sEVs are complex carriers containing diverse RNAs, proteins, and lipids. While our experiments revealed the crucial role of sEVs‐circ6718 in regulating GC‐MSCs activation, the synergistic regulatory effects of other active molecules should not be overlooked. Future research will integrate proteomics and multi‐omics analyses to elucidate the actions of other active components within sEVs on GC‐MSCs, further identifying therapeutic targets for GC.

In conclusion, our study elucidates the role of circ6718 in GC progression via the hsa‐miR‐561‐3p/SAAL1/PRRX1/TGFβ1 axis and the activation of GC‐MSCs mediated by sEVs activation, positioning it as a potential biomarker and therapeutic target. Targeting circ6718 may disrupt both tumor cells and the remodeling of TME. Future studies should validate its clinical utility, explore immune interactions, and investigate sEVs‐based therapies to accelerate translation. This work advances the diagnosis and treatment of GC and provides insights into the communication mediated by sEVs and circRNA between tumors and stroma.

## 4 Materials and Methods

### 4.1 Human Study

In this study, a total of 16 paired tumor and adjacent non‐tumor tissue samples were collected, along with 185 serum samples from patients diagnosed with GC. Furthermore, 78 serum samples from individuals with chronic atrophic gastritis (CAG) and 50 serum samples from healthy donors (GCN) were obtained. All samples were collected between September 2021 and December 2025 at Wujin Hospital Affiliated with Jiangsu University. Comprehensive clinical and pathological data, including age, gender, and tumor size, were extracted from patient records. Written informed consent was obtained from all participants. This study was approved by the Institutional Ethical Committee of Jiangsu University (approval numbers: 2020161) and Wujin Hospital Affiliated with Jiangsu University (approval numbers: 2026‐SR‐030). The inclusion criteria specified that participants must have a confirmed pathological diagnosis of GC with complete pathological data. Patients with had other forms of cancer or those who received chemotherapy or radiotherapy prior to surgery were excluded from the study.

### 4.2 Mouse Tumor Models

Male BALB/c nu/nu mice (aged 4 to 6 weeks, C000103, Cavens, China) were randomly divided into groups (n = 4 or 6 per group). Each mouse received subcutaneous injections of HGC‐27 cells under various treatment conditions or co‐injections with GC‐MSCs to establish a subcutaneous xenograft tumor model (4 × 10^6^ cells in 200 µL of PBS per mouse) or intraperitoneal injections to create a peritoneal metastasis model (4 × 10^6^ cells in 200 µL of PBS per mouse). For tail vein injection of sEVs, the collected sEVs were adjusted to a concentration of 1 × 10^7^ particles/µL. sEVs were injected via the tail vein every 3 days, starting 7 days after the establishment of the tumor or metastatic tumor model. All animal experiments were approved by the Institutional Animal Care and Use Committee of Jiangsu University (UJS‐IACUC‐2024051901) and were conducted in strict accordance with the Guide for the Care and Use of Laboratory Animals.

### 4.3 Cell Lines

The cell lines utilized in this study were obtained from Shanghai EK‐Bioscience Biotechnology (Shanghai, China), ensuring their authenticity and consistency. AGS cells were cultured in F12 medium (MA0214‐2, Meilunbio, China), while SNU‐1 cells were maintained in RPMI‐1640 medium (MA0215‐2, Meilunbio, China). HGC‐27, GES‐1 and HEK293T cells were cultured in high glucose DMEM medium (MA0212‐2, Meilunbio, China). Each of three basal culture media was supplemented with 10% fetal bovine serum (FSP050, Excell, China). All cells were incubated at 37°C in a humidified atmosphere containing 5% CO_2_. Detailed information regarding the cell lines used is presented in Table S3 (Supplementary Information). Throughout the experimental process, the cell lines employed were confirmed to be free from mycoplasma contamination.

### 4.4 Microarray Analysis of Serum sEVs‐circRNAs

We collected samples from three healthy individuals and three patients diagnosed with gastric cancer. All samples were processed using the miRNeasy serum/plasma kit (217184, Qiagen, Germany). CircRNA microarray analyses were conducted by Beijing Capital Biotechnology (Beijing, China). Differentially expressed circRNAs were identified based on the criteria of a p‐value less than 0.05 and an absolute fold change of at least 2. We employed statistical methods, including t‐tests or ANOVA, to screen for differentially expressed circRNAs.

### 4.5 Serum sEVs Isolation

*s*EVs were isolated from human serum using the ExoQuick exosome precipitation solution (EXOQ20A‐1, SBI, USA). The successful isolation was rigorously validated through a trifecta of analyses: transmission electron microscopy (TEM) for morphological characterization, nanoparticle tracking analysis (NTA) to assess particle size distribution, and Western blot to confirm the presence of sEVs markers. It is important to clarify that our detection of sEVs‐circ6718 was based on measuring its expression levels following the extraction of sEVs from whole serum samples obtained from gastric cancer patients.

### 4.6 Genomic DNA (gDNA) Extraction, RNA Isolation, and Reverse Transcription‐Quantitative Polymerase Chain Reaction (RT‐qPCR)

Genomic DNA was extracted using the Universal Genomic DNA Extraction Kit (DP705, Tiangen, China) in accordance with the manufacturer's instructions. Total RNA was isolated with Trizol reagent (15596026, ThermoFisher Scientific, USA) and followed by reverse transcription into cDNA using the PrimeScript RT Reagent Kit (R312‐01/02, Vazyme, China) according to the manufacture's protocol. For miRNA analysis, reverse transcription was performed using the miRNA First Strand cDNA Synthesis (Trailing Reaction) Kit (Sangon Biotech, China). All generated cDNAs were subsequently subjected to qPCR on an ABI real time PCR system (Thermo Fisher Scientific, MA, USA). The relative expression levels of circRNAs were quantified using 2^−ΔΔCt^ method, with β‐actin serving as an endogenous control gene. To validate the qPCR products, they were separated by 1.5% agarose gel electrophoresis, visualized under ultraviolet (UV) light, and further authenticated through Sanger sequencing. A comprehensive list of primer sequences utilized in this study is provided in Table S2 (Supporting Information).

### 4.7 Isolation of Nuclear and Cytoplasmic RNA and RNase R Treatment

Cytoplasmic RNA was isolated using the PARIS Kit (AM1921, Life Technologies, USA), while the remaining nuclear RNA was extracted with Trizol reagent. For RNase R treatment, 2 µg of total RNA was incubated with 6U of RNase R enzyme in 1 × reaction buffer (RNR07250, Epicentre, USA) for 30 min at 37°C. The reaction was terminated by adding 2 µL of 3 M sodium acetate and 50 µL of pre‐cooled anhydrous ethanol, followed by storage at −20°C.

### 4.8 RNA Fluorescence In Situ Hybridization

RNA FISH for circ6718 was performed using the RNA FISH kit (F32202/50, GenePharma, China). The procedure began with the preparation of cell slides, which underwent a series of steps including thorough washing, meticulous fixation, and precise permeabilization. Following this, these slides were incubated overnight at 37°C in a humid chamber to ensure optimal hybridization conditions. The nuclei were stained with 4,6‐diamidino‐2‐phenylindole (DAPI) (D9542, Sigma, USA) for 10 min at room temperature. Subsequently, the cells were washed with cold PBS and assembled using an anti‐quenching buffer. Laser scanning confocal microscopy (Nikon, Japan) was employed to visualize and capture images. The probe sequence for circ6718 was as follows: 5’‐ CAACATCTCCACAAGCCAAGCCAGATAAGTAATGTTTTCA ‐3’.

### 4.9 Transfection, Oligonucleotides, and Plasmids

Specific targeting of siRNA, miRNA mimics, inhibitor, and overexpression plasmids was designed and synthesized by GenePharma (Shanghai, China). Cells were seeded at a density of 2 × 10^5^ cells per well in 6‐well plates and cultured overnight until they reached 50%–70% confluency. The transfection of plasmids, siRNA, miRNA mimics, or inhibitors was performed in serum‐free medium using Lipofectamine 2000 (11668019, Invitrogen, USA) according to the manufacturer's instructions. 6 h post‐ transfection, the medium was replaced with complete growth medium. Cells were harvested 48 h after transfection for RNA analysis or functional assays, and 72 h post‐transfection for protein analysis.

### 4.10 Dual‐Luciferase Reporter Gene Assay

Cells were cultured in 24‐well plates and subsequently transfected with various plasmids, including control vectors, wild‐type (WT) vectors, and mutant (MUT) vectors, that containing miRNA binding sites specific to circ6718 and SAAL1, along with their respective predicted miRNA mimics and control oligonucleotides (GenePharma, Suzhou, China). To further explore the investigate the underlying regulatory mechanisms, luciferase plasmids were custom‐designed and synthesized to include the promoter regions of PRRX1 and TGFβ1. These constructions enabled the assessment of how different treatments affected the transcriptional activity of these genes. Notably, targeted mutations were introduced into the TGFβ1 promoter region to disrupt its putative binding site. Mutation‐1 affected nucleotides 138–145, while Mutation‐2 targeted nucleotides 611–618. The transfection of these plasmids into the cells was conducted using Lipofectamine 2000 reagent. Following a 48‐h incubation period post‐transfection, the dual‐luciferase reporter assay kit (DL101‐01, Vazyme, China) was utilized to quantify luciferase activities, in accordance with the manufacturer's instructions.

### 4.11 Chromatin Immunoprecipitation

ChIP analysis was conducted using the Pierce Agarose ChIP Kit (26156, Thermo Scientific, USA). Immunoprecipitation of chromatin was performed with normal rabbit IgG (as a negative control, I5381, Millipore, USA, RRID: AB_1163670) and anti‐PRRX1 antibodies (A10237, Abclonal, China, RRID: AB_2757762). The 1% supernatant fraction of chromatin, which was not treated with primary antibody, was retained as the “input sample.” Real‐time RT‐PCR was performed using Maxima SYBR Green qPCR Premix (Q511‐02, Vazyme, China) with primers specifically designed to amplify the promoter regions of selected genes. The primers used were as follows: TGFβ1 promoter, sense: 5’‐ TGTTTTCCCTCACAGCAA‐3’; antisense: 3’‐AAGCAAATTATAGGGTAGATCA‐5’.

### 4.12 RNA–Protein Immunoprecipitation (RIP)

The RIP assay was conducted using the EZ‐Magna RIP RNA binding protein immunoprecipitation kit (RIP‐12RXN, Millipore, USA), following the manufacturer's protocol meticulously. Cells at approximately 90% confluence were lysed in a complete RIP lysis buffer, which was supplemented with RNase inhibitors and protease inhibitors. Prior to the immunoprecipitation step, magnetic beads were incubated with the anti‐Ago2 antibody (SAB4200085, Millipore, USA, RRID: AB_10600719) for 1 h at room temperature to facilitate the formation of the antibody‐bead complex. Subsequently, cell lysates were added to the antibody‐coated beads and allowed to interact overnight at 4°C, ensuring specific binding of RNA‐protein complexes. The immunoprecipitated RNA‐protein complexes were then purified and quantified using qRT‐PCR, with normal rabbit IgG serving as a negative control.

### 4.13 Co‐Immunoprecipitation (Co‐IP) Assay

Cells were harvested and lysed in Co‐IP buffer (P0013, Beyotime, China). The resulting lysates were incubated with SAAL1 antibody (A13183, Abclonal, China, AB_2760034) at 4°C overnight. Following this, the lysates were treated with protein A agarose beads (P2012, Beyotime, China) at 4°C for 4 h. After incubation, the protein complexes were washed with fresh Co‐IP buffer to eliminate any non‐specifically bound proteins. Finally, the precipitated protein complexes were resolved and analyzed via Western blot to confirm the presence of the target protein.

### 4.14 Lentivirus Infection

Gastric cancer cells (8 × 10^4^ cells) were seeded in 6‐well plates. Once the cells were adhered, the lentiviral vectors were diluted and mixed with serum‐free medium in the 6‐well plates. Fluorescence of mCherry or GFP was observed 48 h post‐infection. Puromycin was diluted to 2 µg/mL using complete medium to screen for mCherry or GFP‐positive cells. The screening process continued for 3–5 days, after which fluorescence was monitored. Screening was discontinued when the percentage of positive cells exceeded 90%. Based on the expression levels of circ6718 in sEVs, we transfected shRNA into SNU‐1 cells, which express circ6718 at relatively high levels, and collected sEVs with circ6718 knocked down (sh‐circ6718‐sEVs). In AGS cells, which express circ6718 at relatively low levels, we transfected lentivirus overexpressing circ6718 (OE‐circ6718) and collected circ6718 overexpressing sEVs (OE‐circ6718‐sEVs).

### 4.15 Cell Counting Kit‐8 (CCK‐8) and Colony Formation Assays

For CCK8 assay, GC cells were seeded into 96‐well plates at a density of 1 × 10^3^ per well, with triplicate wells maintained for each time point ranging from 1 to 4 days. Following this, 10 µL of CCK‐8 solution (A311‐02, Vazyme, China) was added to each well, and the plates were incubated at 37°C for 2 h. Cell viability was assessed by measuring the absorbance at 450 nm using an enzyme marker (BioTEK, USA). For colony formation assay, GC cells were plates at a density of 1 × 10^3^ cells per well. The medium was refreshed every 3 days throughout the culture period. After 10 to 14 days, the colonies that formed were fixed with 4% paraformaldehyde and then stained with 0.5% crystal violet. The colonies were photographed, and their numbers were accurately counted.

### 4.16 Transwell Migration and Matrigel Invasion Assays

Cells were resuspended in serum‐free medium and introduced into the upper compartment of Transwell chambers (3422, Corning, USA), with or without the addition of matrix gel (356234, BD Biosciences, USA) to simulate the extracellular matrix for invasion assays. The lower chamber was filled with 600 µL of complete medium, serving as a chemoattractant. Plates were then incubated at 37°C in a 5% CO_2_ atmosphere to facilitate cell migration and invasion. After incubation, cells that migrated or invaded through the pores of the upper chamber were fixed with a 4% paraformaldehyde solution and stained with crystal violet dye for microscopic observation.

### 4.17 Immunofluorescence

Cells were fixed on slides using 4% paraformaldehyde for 30 min, permeabilized with 0.1% Triton X‐100 for 15 min, and subsequently blocked with 5% BSA for 1 h. Following this, the cells were incubated with the primary antibody overnight at 4°C. The next day, a fluorescently labeled secondary antibody was applied to the sections and incubated for 1 h at 37°C. Subsequently, the cell nuclei were stained with DAPI. The sections were then observed and imaged using a confocal microscope (DeltaVision Elite, GE, USA).

### 4.18 Western Blot

Total protein from GC cells was harvested on ice using RIPA lysis buffer (89901, ThermoFisher Scientific, USA) supplemented with protease inhibitors. Equal quantities of protein were loaded and separated by SDS‐polyacrylamide gel electrophoresis (SDS‐PAGE). Subsequently, the proteins were electro transferred onto polyvinylidene fluoride (PVDF) membranes (IPVH00010, Millipore, USA). The membranes were then blocked in 5% (w/v) skimmed milk and incubated overnight at 4°C with specific primary antibodies. The dilutions of the primary antibodies ranged from 1:300 to 1:500 and were incubated overnight at 4°C. The antibodies used are listed in Table S3 (Supporting Information). After washing three times with tris‐buffered saline containing Tween 20 (TBS‐T), the membranes were incubated with the appropriate horseradish peroxidase‐conjugated secondary antibodies (anti‐rabbit #31460, Invitrogen, RRID: AB_228341) or anti‐mouse (#31430, Invitrogen, RRID: AB_228307) from Invitrogen) for 1 h at room temperature. Protein bands were visualized using an enhanced chemiluminescence system (ImageQuant LAS4000mini, GE, Japan).

### 4.19 sEVs Extraction and Identification

The conditioned medium underwent a series of centrifugation steps, initially at 300×g for 15 min, followed by 2000×g for an additional 15 min. Subsequently, it was centrifuged at 10 000×g for 30 min. Thereafter, the supernatant was filtered through a 100 kDa ultrafiltration centrifuge tube. The filtrate was then subjected to ultracentrifugation at 100 000×g for 70 min (Beckman Coulter, China) twice to collect the sEVs. The properties of the sEVs were characterized using transmission electron microscopy (TEM), nanoparticle tracking analysis (NTA), and Western blot.

### 4.20 Isolation and Culture of GC‐MSCs

GC Tissues Were Procured from Patients Undergoing Surgical Procedures At the Jiangsu University Affiliated Hospital. GC‐MSCs Were Isolated and Cultured Following a Previously Established protocol [11]. Fresh tissue Samples, carefully minced into fragments of approximately 1mm^3^, were placed in 35 mm cell culture dishes (153066, Nunc, Denmark) and cultured in α‐MEM medium (C12571500BT, Gibco, USA) supplemented with 10% fetal bovine serum. The cultures were incubated at 37°C in a 5% CO_2_ environment, with the medium changes occurring every 3 days until fibroblast‐like cells reached approximately 80% confluence. The identification of fibroblast‐like cells was conducted through adipogenic and osteogenic assays. The surface markers (CD44, CD73, CD45, CD14 and CD34) (397517, 127219, 982316, 982502, 343503, BioLegend, USA, RRID: AB_2888763, AB_2716075, AB_2876779, AB_2616906, AB_1731923) on GC‐MSCs were analyzed using flow cytometry.

### 4.21 Hematoxylin‐Eosin (HE) Staining and Immunohistochemistry

For HE staining, paraffin‐embedded tissue sections were subjected to deparaffinization in xylene, followed by rehydration through a series of ethanol solutions with decreasing concentrations. Subsequently, the sections were washed in PBS and stained with HE. For IHC staining, a streptavidin‐biotin complex (SABC) kit (SA1020, Boster, China) was utilized. The sections were dewaxed in xylene, rehydrated using graded ethanol and boiled in citrate buffer (10 mM, pH 6.0) for 30 min to recover antigen. After treatment with 3% hydrogen peroxide for 10 min to inhibit endogenous peroxidase activity, the sections were blocked with 5% bovine serum albumin (BSA), and incubated with primary antibody overnight at 4°C, followed by the secondary antibody for 30 min at 37°C, and SABC for an additional 30 min at 37°C. Finally, the tissue sections were visualized using diaminobenzidine (DAB) and counterstained with hematoxylin for microscopic examination. After mounting in a neutral resin medium, the sections were scanned using a digital slide scanner (Panoramic MIDI 3DHistech, Hungary).

### 4.22 Masson Staining

Tumor tissues were excised and fixed in a 4% paraformaldehyde solution for 24 h. Subsequently, the tissues were dehydrated using a gradient ethanol series, embedded, and sectioned. The slides of tumor tissues from mice in each group were stained using Masson staining (Solarbio, Beijing, China, G1346) in accordance with the manufacturer's instructions.

### 4.23 Sirius Red Staining

The tumor tissues were excised and fixed in 4% paraformaldehyde solution for 24 h. Subsequently, the tissues were dehydrated using a gradient of ethanol, embedded and sectioned. The tumor tissue slides from each group of mice were stained with Sirius Red (Solarbio, Beijing, China, G1472) according to the manufacturer's instructions.

### 4.24 Statistical Analysis

All biological replicates were conducted with a minimum of three repetitions. The data are presented as the mean ± standard deviation (SD). Normal distribution was assessed using the Pearson normality test and the D'Agostino test. Statistical analyses were performed using Prism 9.0 software (GraphPad, USA). To compare the significance of differences between two or more groups, we utilized the Student's t‐test and one‐way ANOVA. For comparisons involving more than two groups, one‐way analysis of variance (ANOVA) was employed. Spearman's correlation analysis was conducted to assess the associations. Survival time was analyzed using the Kaplan‐Meier method and a log‐rank test. A *p*‐value of <0.05 was considered statistically significant.

## Author Contributions

F.Z., X.Z., and D.W. contributed equally to this work. Conceptualization, X.Z., D.W., R.L., H.Q., W.X., Y.Y., and F.Z.; writing – original draft, F.Z., X.Z., and D.W.; data curation, F.Z., X.Z., D.W., and R.L.; software, F.Z., and X.Z.; investigation, D.W., and R.L.; resources, H.Q., and W.X.; validation, D.W., and R.L.; revised manuscript, Y.Y., J.J., W.X., and F.Z.; funding acquisition, Y.Y., J.J., W.X., and X.Z.; supervision, Y.Y., and J.J.; project administration, Y.Y., and J.J.

## Conflicts of Interest

The authors declare no conflict of interest.

## Supporting information




**Supporting File**: advs75645‐sup‐0001‐SuppMat.docx.

## Data Availability

The data that support the findings of this study are available on request from the corresponding author. The data are not publicly available due to privacy or ethical restrictions.
