# Supplementary material for: Small Extracellular Vesicles‐Derived Circ6718 Unlocks Stromal Remodeling and Serves as a Biomarker in Gastric Cancer
Source: Adv Sci (Weinh). 2026 May 11;13(43):e21334. doi: 10.1002/advs.202521334 (PMC13335969; doi:10.1002/advs.202521334)
Supplement: Supplementary file 1 — Supporting File: advs75645‐sup‐0001‐SuppMat.docx. [file ADVS-13-e21334-s001.docx]

**Supporting Information for Small Extracellular Vesicles-Derived Circ6718 Unlocks Stromal Remodeling And Serves As A Biomarker In Gastric Cancer**

Fan Zhang^1,3#^, Xueyan Zang^2,3#^, Dongli Wang^2,3#^, Rong Li^3^, Hui Qian^3^, Wenrong Xu^2,3^, Jiajia Jiang^2,3*^, Yongmin Yan^1, 3*^

^1^Department of Laboratory Medicine, Wujin Hospital Affiliated with Jiangsu University, Changzhou, 213017, Jiangsu, P.R. China;
^2^Aoyang Institute of Cancer, Affiliated Aoyang Hospital of Jiangsu University, 279 Jingang Road, Suzhou, 215600, Jiangsu, P.R. China;

^3^Jiangsu Key Laboratory of Medical Science and Laboratory Medicine, Department of Laboratory Medicine, School of Medicine, Jiangsu University, 301 Xuefu Road, Zhenjiang, 212013, Jiangsu, P.R. China;

^#^These authors contributed equally to this work.

*Corresponding author. Email: yym@ujs.edu.cn (Y.-M.Y.), jiangjiajia_2001@163.com (J.-J.J.)

**Supplementary Materials**


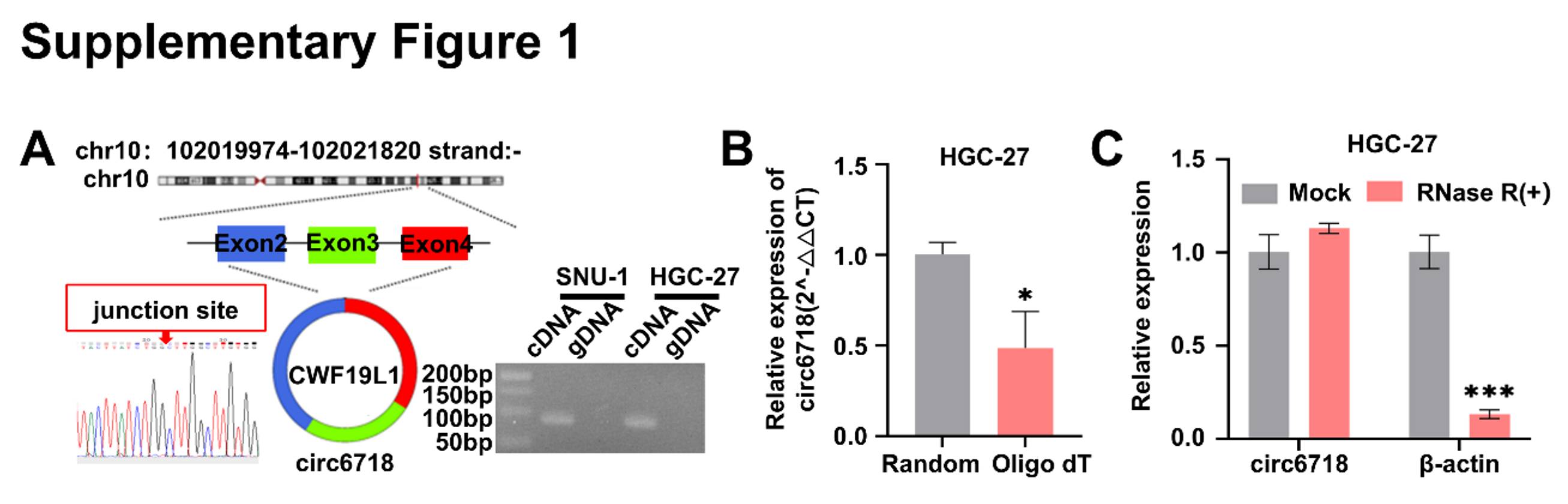


### Supplementary Figure 1. Circular structure of circ6718.

1. Schematic diagram illustrating the ring structure of circ6718 is presented, alongside the results from Sanger sequencing that detect of splice sites and the outcomes of agarose gel electrophoresis experiments. **B.** Circ6718 was amplified using oligo dT and random primers (n = 3). **C.** qRT-PCR analysis showing the abundance of circ6718 in GC cells following treatment with RNase R (n = 3). The data were plotted as Mean ± SEM. Statistical significance is indicated as follows: *p < 0.05, ***p < 0.001 by Student's t‐test for B; by one‐way ANOVA for C.


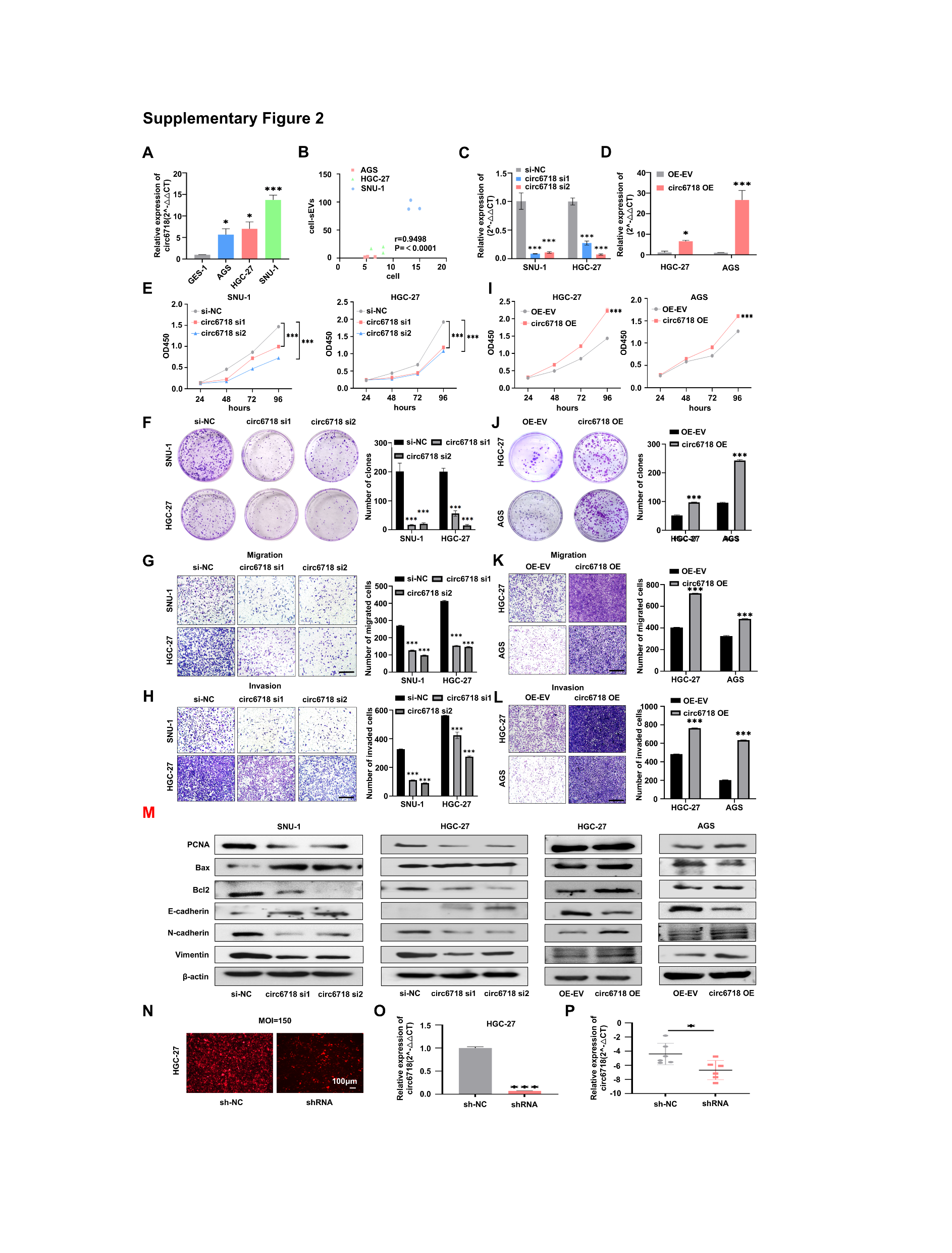


**Supplementary** **Figure 2. Circ6718 promotes GC progression *in vitro*.**

**A.** The expression levels of circ6718 in GC cells (n = 3). **B**. The correlation of circ6718 expression levels between GC cells and sEVs was evaluated using qRT-PCR (n = 3). **C.** The levels of circ6718 in GC cells treated with two specific siRNAs targeting circ6718 were assessed through qRT-PCR (n = 3). **D.** qRT-PCR was conducted to evaluate the efficiency of circ6718 overexpression in GC cells (n = 3). The CCK-8 assay (**E**), colony formation (**F**), Transwell migration (**G**), and matrigel invasion assays (**H**) were conducted for control, circ6718 si1, and circ6718 si2 transfected GC cells (scale bar = 200μm) (n = 3). Further, CCK-8 assay (**I**), colony formation (**J**), Transwell migration (**K**), and matrigel invasion assays (**L**) were performed on control and circ6718-overexpressing GC cells (scale bar = 200μm) (n = 3). **M.** Western blot analysis was performed to evaluate EMT and proliferation indices following transfection with si-circ6718 and circ6718 overexpression constructs. **N.** Fluorescence imaging was conducted on GC cells that were stably transfected with circ6718 shRNA (scale bar = 100μm). **O.** qRT-PCR analysis was carried out on GC cells stably transfected with circ6718 shRNA (n = 3). **P.** The expression levels of circ6718 were assessed in subcutaneous xenograft tumors derived from mice injected with either control or circ6718 shRNA-transfected GC cells (n = 6 mice/group). The data were plotted as Mean ± SEM. Statistical significance is indicated as follows: *p < 0.05, ***p < 0.001 by Student's t‐test for I, O and P; by one‐way ANOVA for A, C, D, E, F, G, H, J, K and L; by Spearman Pearson correlation analysis for B.


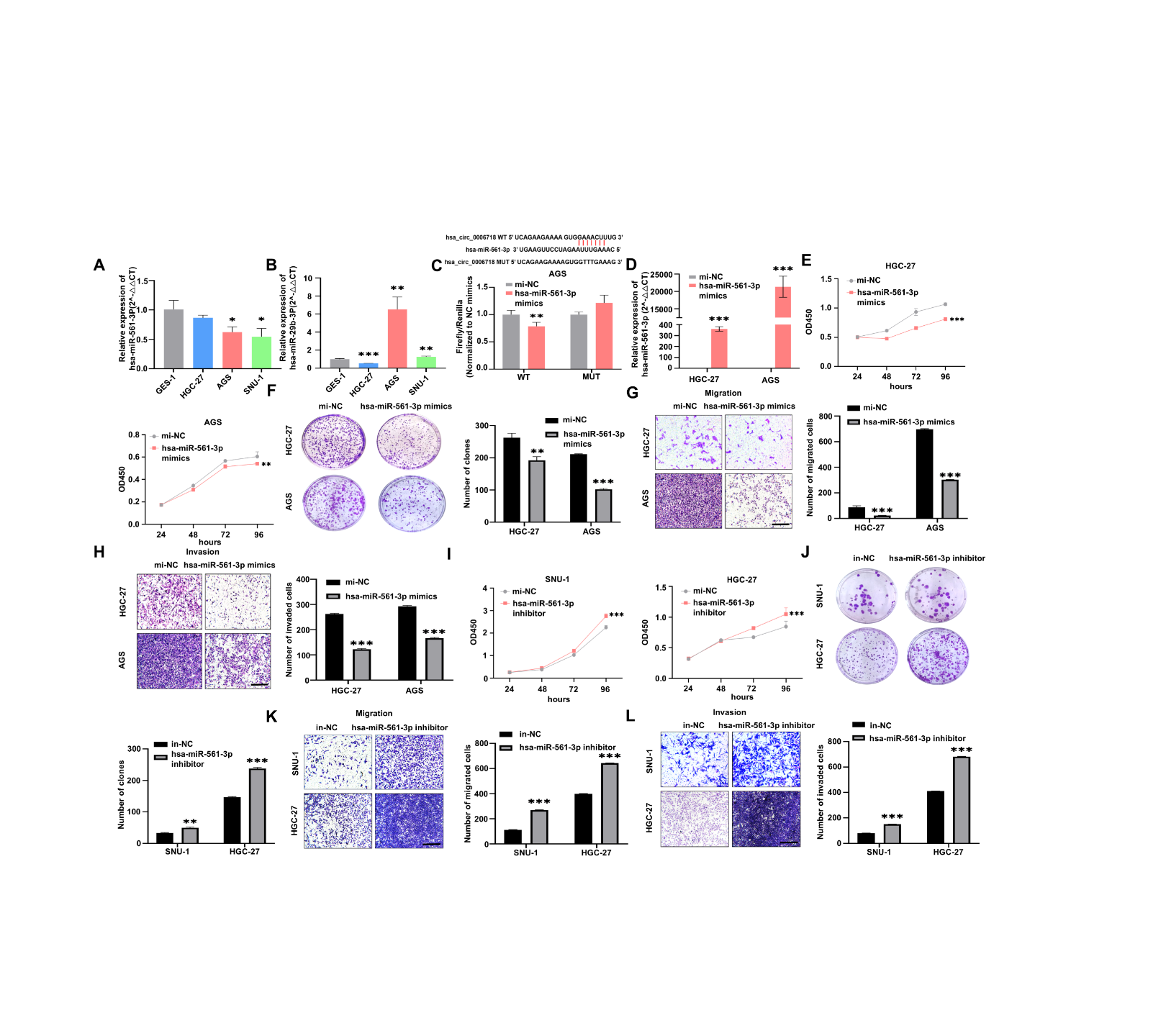


**Supplementary Figure 3. Hsa-miR-561-3p inhibits GC cells progression.**

**A-B.** qRT-PCR was conducted to quantify the expression levels of hsa-miR-561-3p and hsa-miR-29b-3p in GC cell lines (n = 3). **C.** The relative luciferase activity of wild-type and mutant circ6718 constructs co-transfected with hsa-miR-561-3p mimics or a negative control was evaluated (n = 3). **D.** qRT-PCR analysis was performed to assess the transfection efficiency of hsa-miR-561-3p mimics in GC cells (n = 3). Further analyses included the CCK-8 assay (**E**), colony formation assay (**F**), Transwell migration assay (**G**), and matrigel invasion assay (**H**) comparing control and GC cells transfected with hsa-miR-561-3p mimics (scale bar = 200μm) (n = 3). Additionally, CCK-8 assay (**I**), colony formation assay (**J**), Transwell migration assay (**K**), and matrigel invasion assay (**L**) were conducted on control and GC cells transfected with hsa-miR-561-3p inhibitor (scale bar = 200μm) (n = 3). The data were plotted as Mean ± SEM. Statistical significance is indicated as follows: *p < 0.05, **p < 0.01, ***p < 0.001 by Student's t‐test for E and I; by one‐way ANOVA for A, B, C, D, F, G, H, J, K and L.


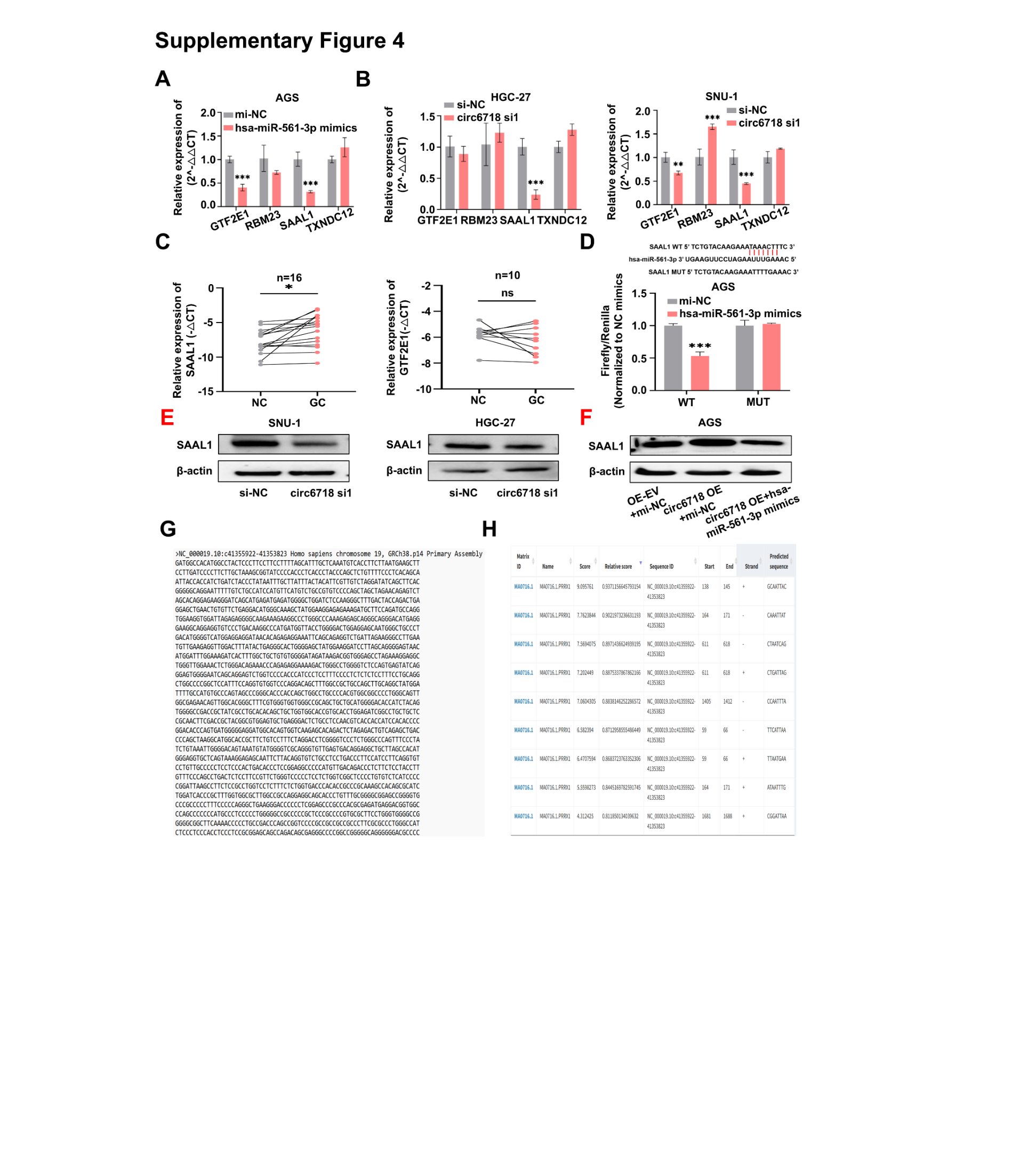


**Supplementary Figure 4. Hsa-miR-561-3p targets SAAL1 in GC cells.**

1. qRT-PCR was performed to evaluate the expression of target genes in GC cells following transfection with hsa-miR-561-3p mimics (n = 3). **B.** qRT-PCR was utilized to assess the expression of target genes in GC cells after transfection with circ6718 siRNA (n = 3). **C.** The expression levels of SAAL1 and GTF2E1 in tumor tissues from GC patients were analyzed using qRT-PCR (n = 16 and 10). **D.** The relative luciferase activity of wild-type and mutant SAAL1 constructs was measured after co-transfected with hsa-miR-561-3p mimics or a miRNA negative control (n = 3). **E.** Western blot analysis was conducted to determine SAAL1 expression following transfection with si-circ6718. **F.** Western blot analysis was also performed to evaluate SAAL1 expression after co-transfection with circ6718 and hsa-miR-561-3p. **G.** The UCSC database was employed to search for upstream promoter regions of TGFβ1. **H.** The JASPAR database was queried to identify binding sites of PRRX1 within the upstream promoter region of TGFβ1. The data were plotted as Mean ± SEM. Statistical significance is indicated as follows: *p < 0.05, **p < 0.01, ***p < 0.001 by Student's t‐test for C; by one‐way ANOVA for A, B and D.

**
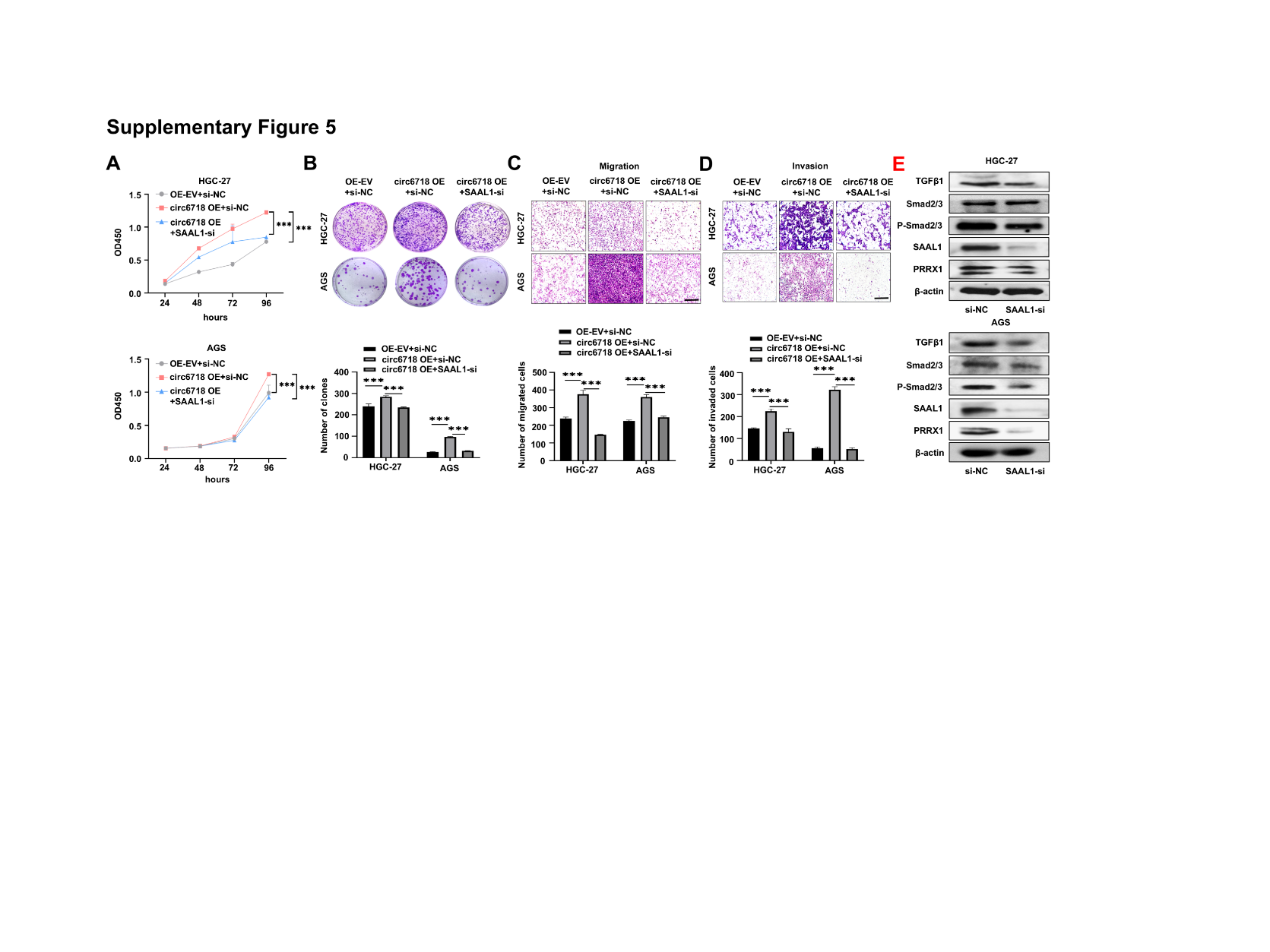
**

**Supplementary Figure 5. Knockdown of SAAL1 inhibits the carcinogenic function of circ6718.**

The biological activity of GC cells, co-transfected with circ6718 overexpression and SAAL1 siRNA, was evaluated through various assays, including CCK-8 assay (**A**), colony formation assay (**B**), Transwell migration assay (**C**), and matrigel invasion assay (**D**) (scale bar = 200μm) (n = 3). Additionally, (**E**) Western blot analysis was conducted to assess the activation of the TGFβ1/Smad2/3 signaling pathway in GC cells transfected with SAAL1 siRNA. The data were plotted as Mean ± SEM. Statistical significance is indicated as follows: ***p < 0.001 by Student's t‐test for E and I; by one‐way ANOVA for A-D.


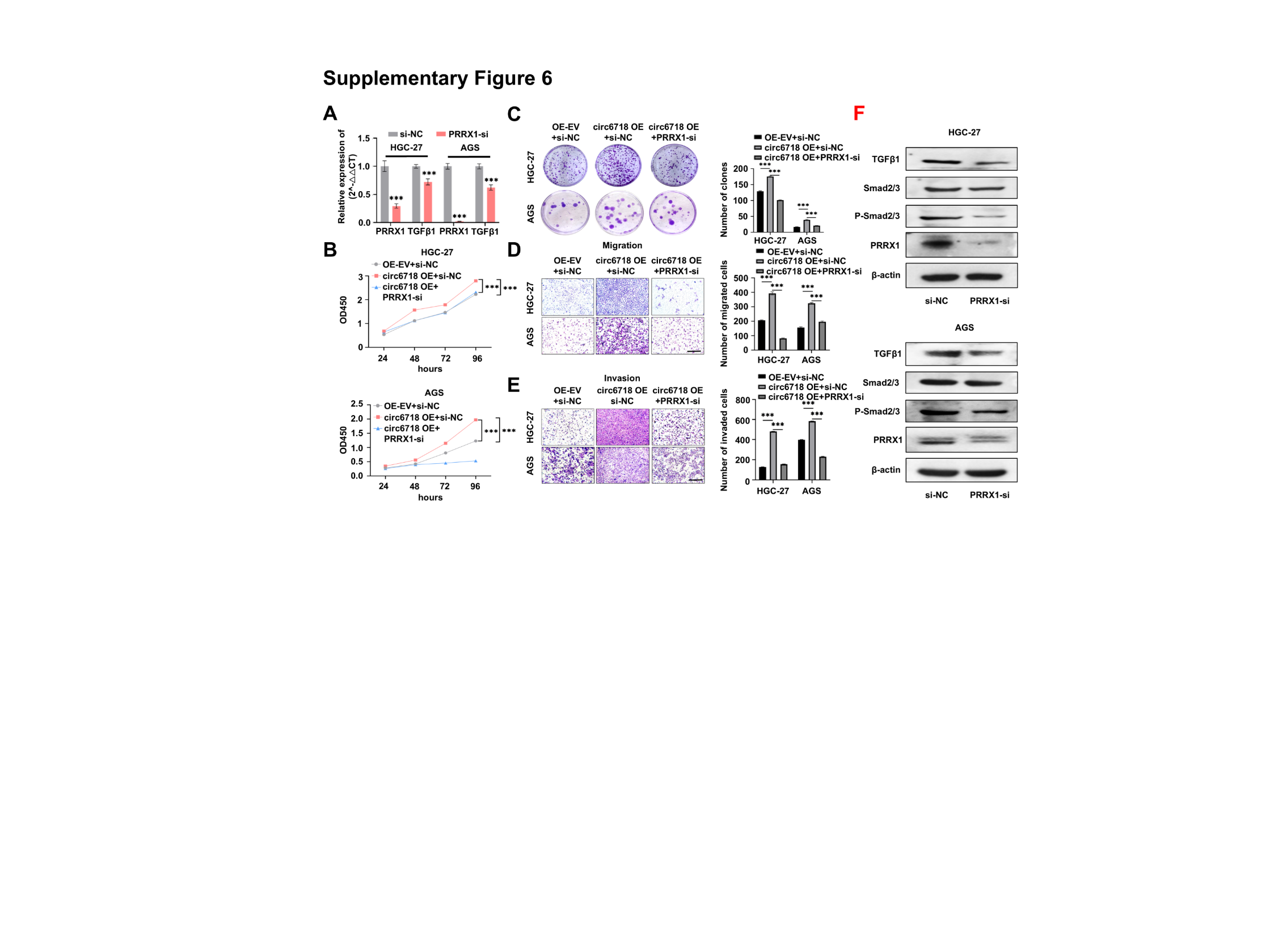


**Supplementary Figure 6. Knockdown of PRRX1 suppresses the carcinogenic function of circ6718.**

**A.** qRT-PCR was performed to assess the expression of PRRX1 in GC cells transfected with PRRX1 siRNA. The biological functions of GC cells co-transfected with circ6718 overexpression and PRRX1 siRNA were evaluated through various assays, including the CCK-8 assay (**B**), colony formation assay (**C**), Transwell migration assay (**D**), and matrigel invasion assay (**E**) were co-transfected with circ6718 overexpression and PRRX1 siRNA in GC cells (scale bar = 200μm) (n = 3). **F.** Western blot analysis was conducted to examine the activation of TGFβ1/Smad2/3 signaling pathway in GC cells transfected with PRRX1 siRNA. The data were plotted as Mean ± SEM. Statistical significance is indicated as follows: ***p < 0.001 by one‐way ANOVA for A-E.


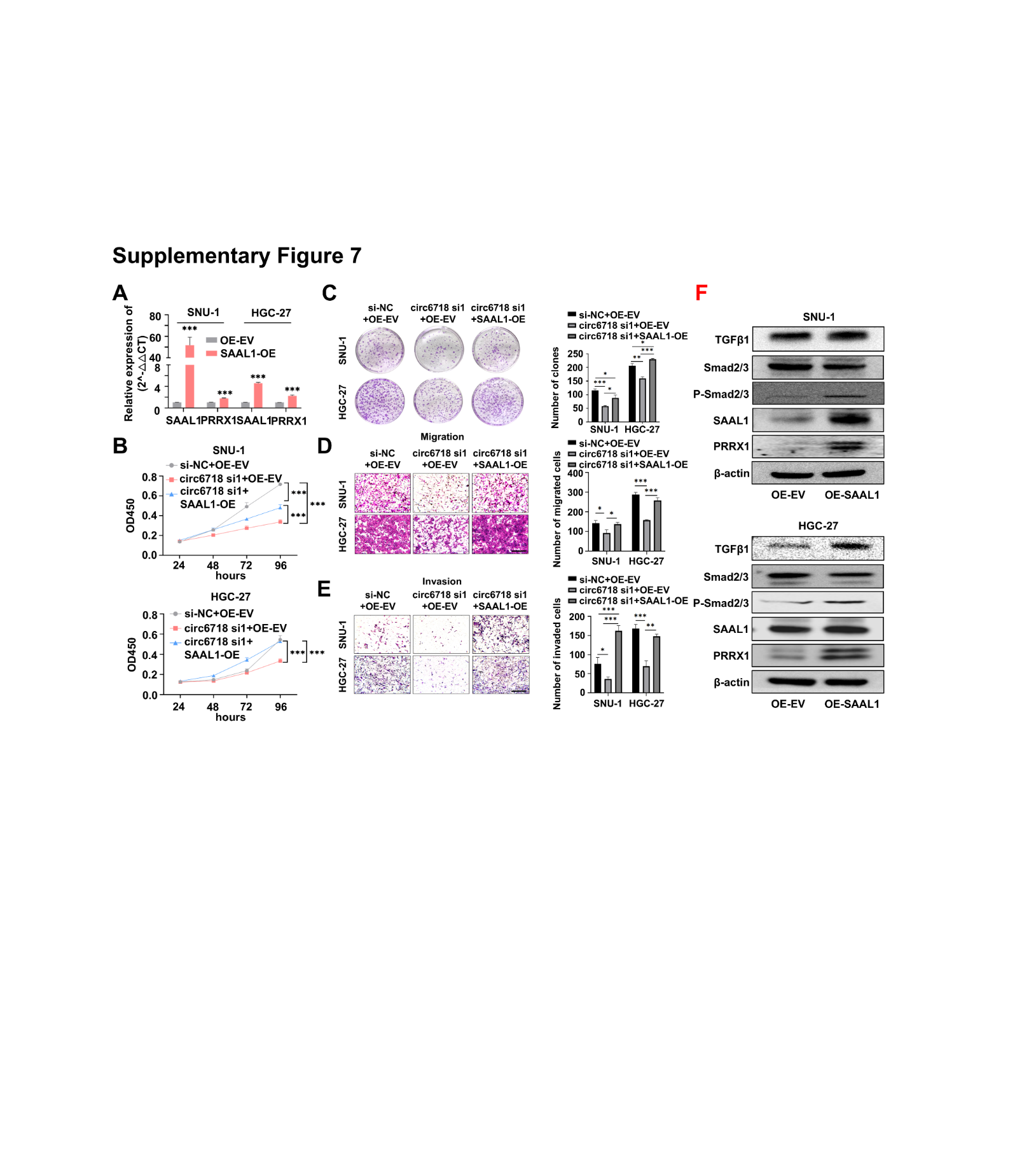


**Supplementary Figure 7. Overexpression of SAAL1 rescued the suppression of GC cells progression induced by circ6718 knockdown.**

**A.** qRT-PCR analysis was conducted to assess the expression levels of PRRX1 in GC cells transfected with SAAL1 overexpression. CCK-8 assay (**B**), colony formation assay (**C**), Transwell migration assay (**D**), and matrigel invasion assay (**E**) were performed following co-transfection with circ6718 siRNA and SAAL1 overexpression in GC cells (scale bar = 200μm) (n = 3). **F.** Western blot analysis was employed to investigate the activation of TGFβ1/Smad2/3 signaling pathway in GC cells transfected with SAAL1 overexpression. The data were plotted as Mean ± SEM. Statistical significance is indicated as follows: *p < 0.05, **p < 0.01, ***p < 0.001 by one‐way ANOVA for A-E.


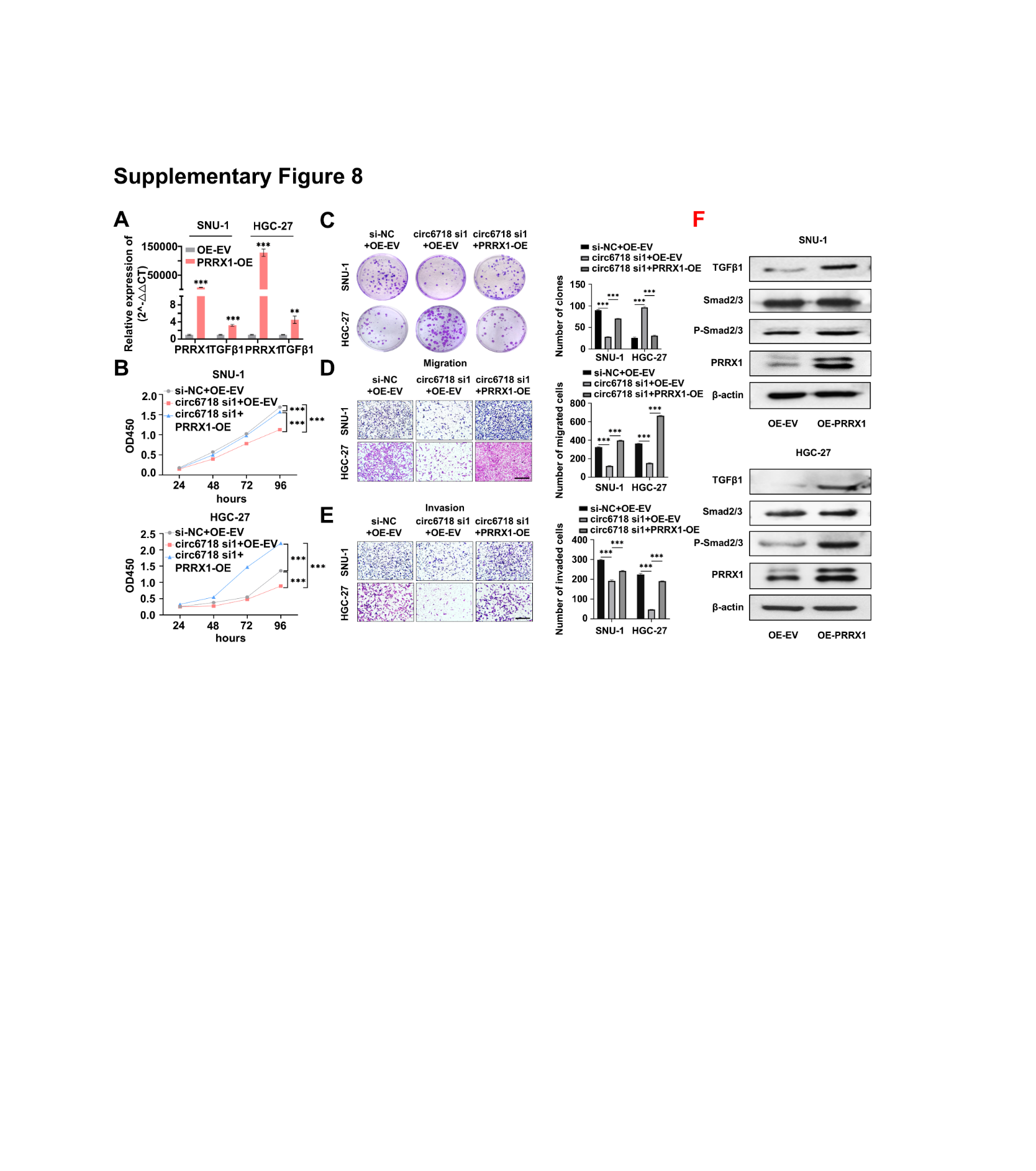


**Supplementary Figure 8. Overexpression of PRRX1 reversed the suppression of GC cells progression induced by circ6718 knockdown.**

**A.** qRT-PCR was conducted to evaluate the expression levels of PRRX1 in GC cells that were transfected with PRRX1 overexpression. CCK-8 assay (**B**), colony formation assay (**C**), Transwell migration assay (**D**), and matrigel invasion assay (**E**) were performed on GC cells co-transfected with circ6718 siRNA and PRRX1 overexpression in GC cells (scale bar = 200μm) (n = 3). **F.** Western blot analysis was employed to assess the activation of TGFβ1/Smad2/3 signaling pathway in GC cells transfected with PRRX1 overexpression. The data were plotted as Mean ± SEM. Statistical significance is indicated as follows: **p < 0.01, ***p < 0.001 by one‐way ANOVA for A-E.


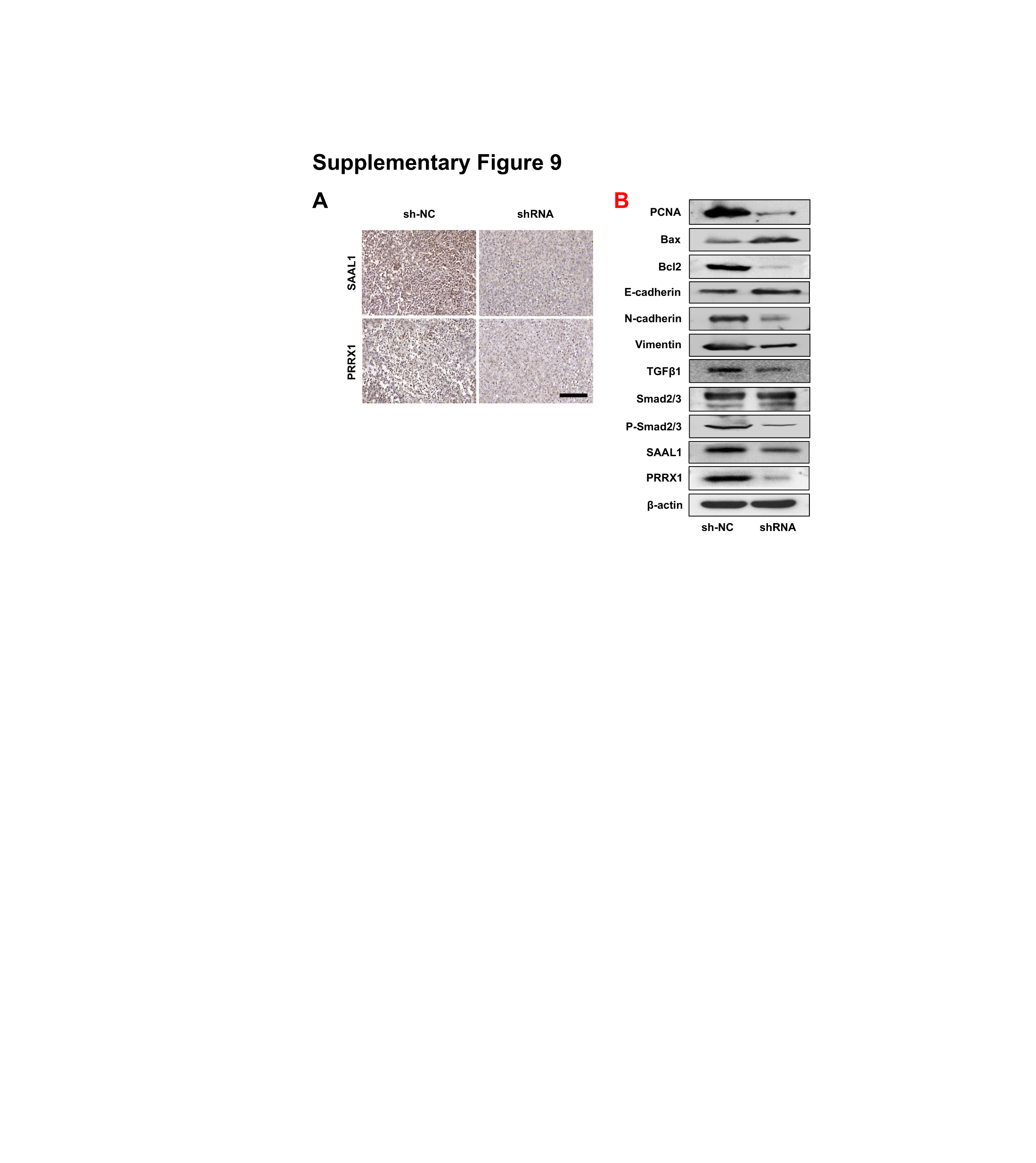


**Supplementary Figure 9. Knockdown of circ6718 inhibits SAAL1/PRRX1 expression *in vivo*.**

**A.** Immunohistochemical staining was conducted on xenograft tumors derived from control and circ6718 shRNA mice (scale bar = 50μm). **B.** Western blot analysis was performed to assess protein expression levels in xenograft tumors from both control and circ6718 shRNA mice.


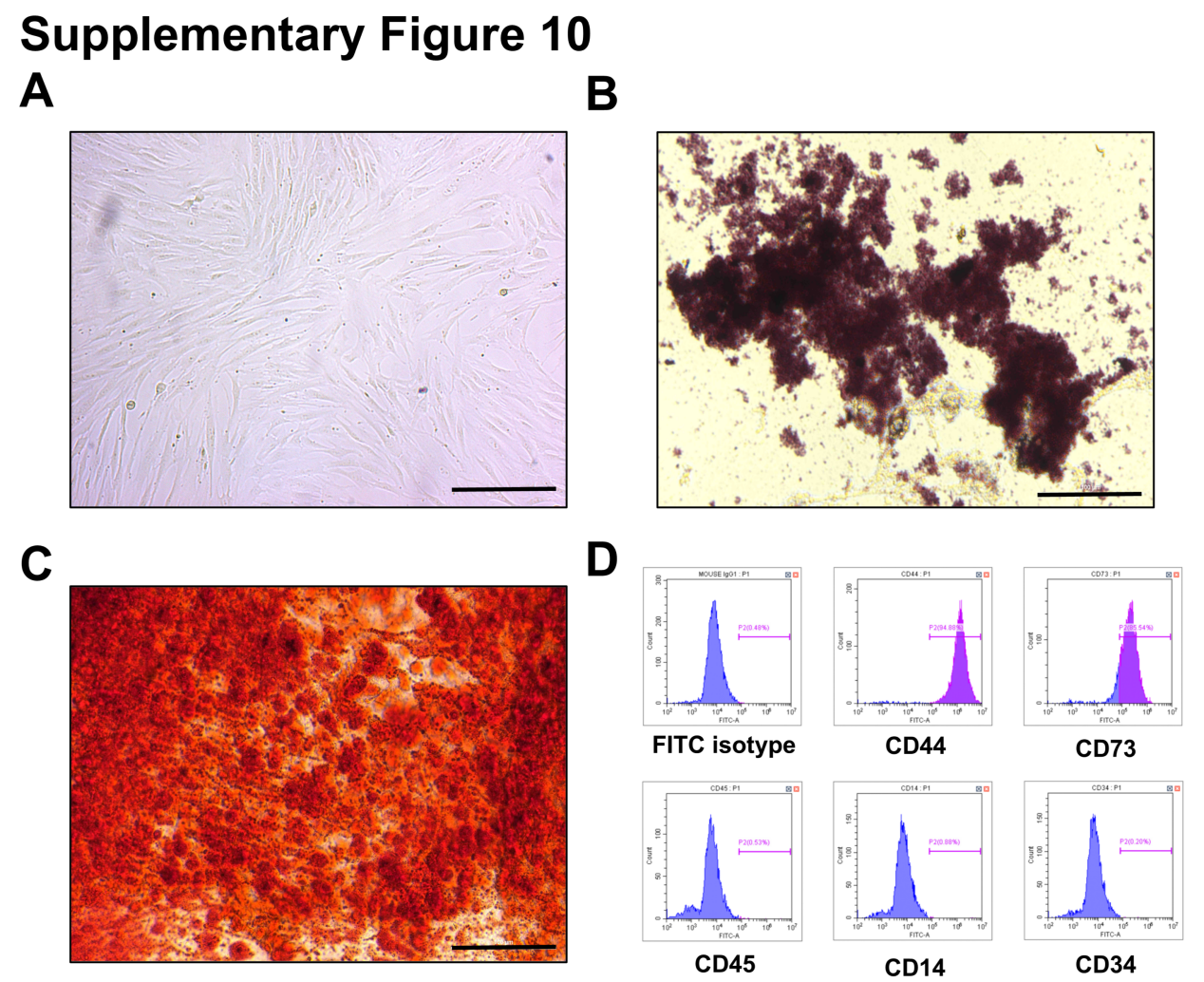


**Supplementary Figure 10. Identification of GC-MSCs.**

**A.** Bright-field image of GC-MSCs in culture (scale bar = 100μm). **B.** Representative images illustrating osteogenesis in GC-MSCs (scale bar = 100μm). **C.** Representative images demonstrating adipogenesis in GC-MSCs (scale bar = 100μm). **D.** Flow cytometry analysis of characteristic molecular markers in GC-MSCs.


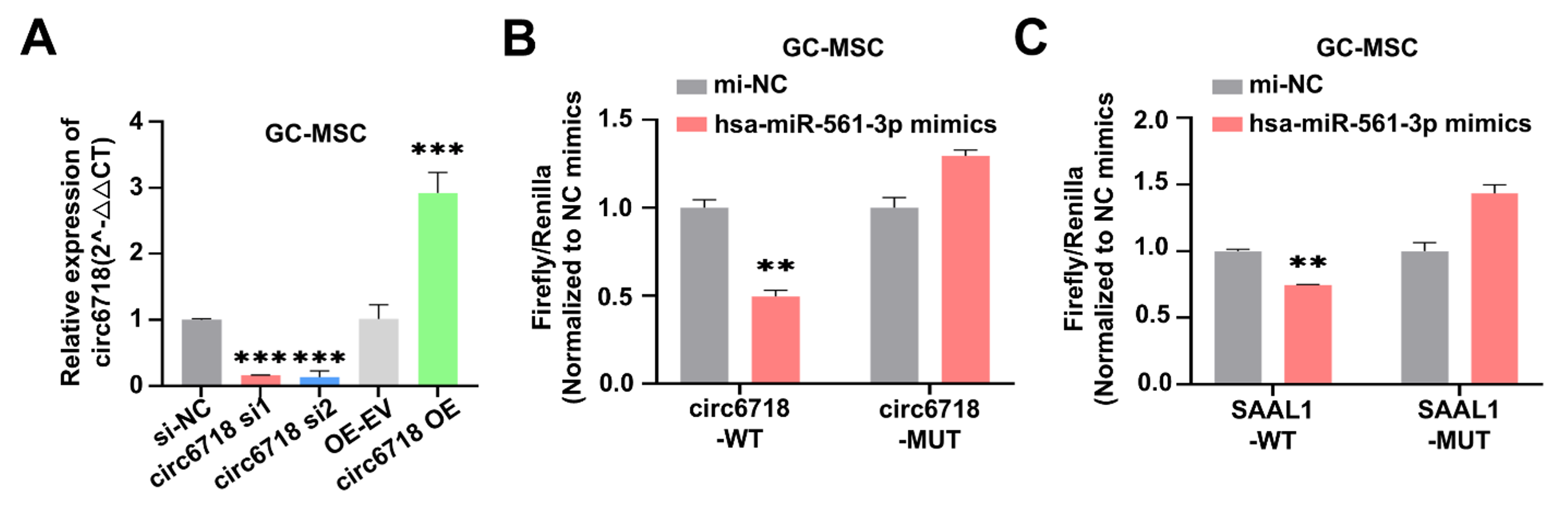


**Supplementary Figure 11. Hsa-miR-561-3p binds to Circ6718 and SAAL1 in GC-MSCs.**

**A.** The efficiency of circ6718 siRNA transfection and overexpression in GC-MSCs was assessed using qRT-PCR (n = 3). **B.** The relative luciferase activity of wild-type and mutant circ6718 constructs was evaluated after co-transfection with hsa-miR-561-3p mimics or a miRNA negative control in GC-MSCs (n = 3). **C.** The relative luciferase activity of wild-type and mutant SAAL1 constructs co-transfected with hsa-miR-561-3p mimics or a miRNA negative control in GC-MSCs (n = 3). The data were plotted as Mean ± SEM. Statistical significance is indicated as follows: **p < 0.01, ***p < 0.001 by one‐way ANOVA for A-C.


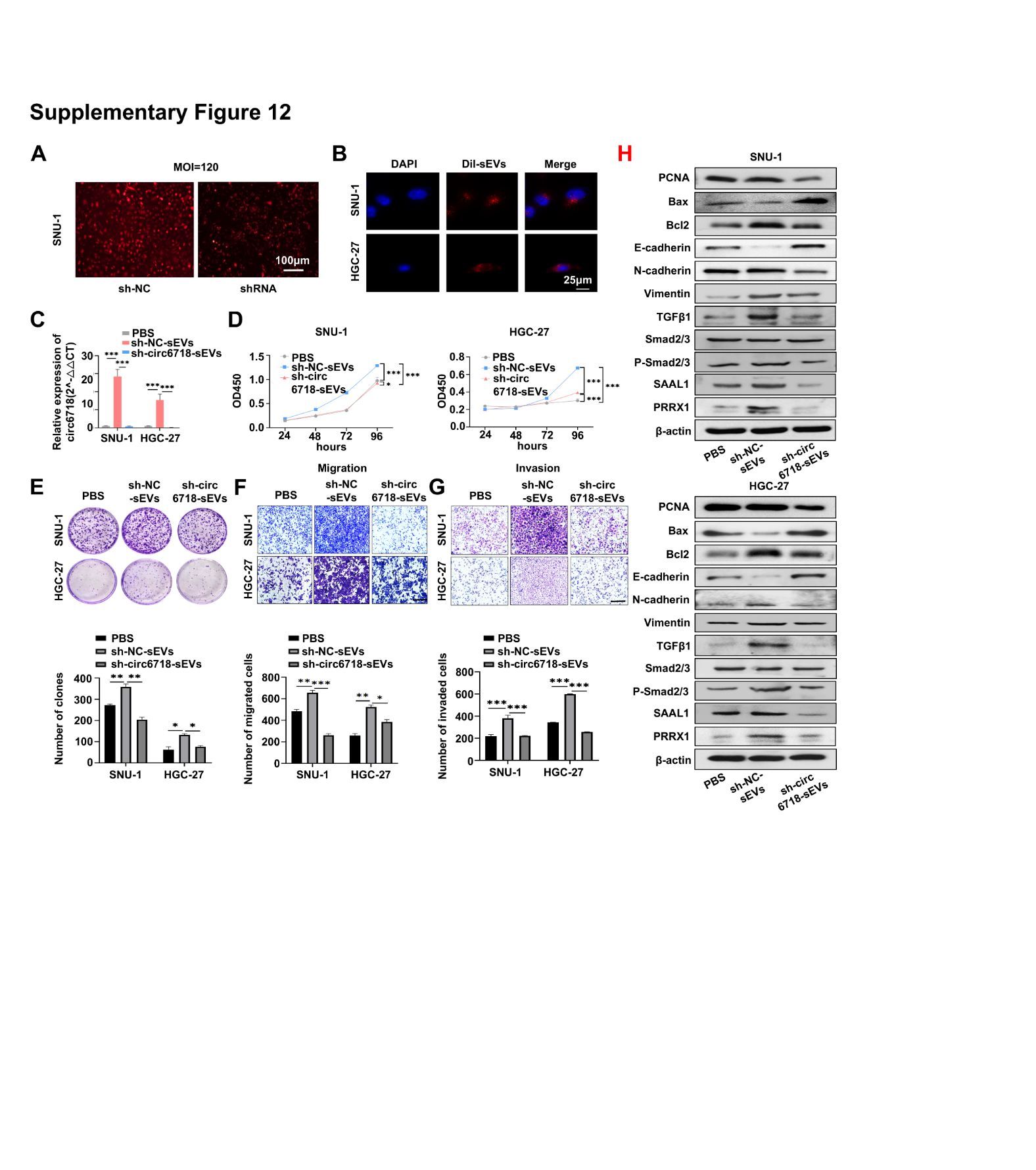


**Supplementary Figure 12. sh-circ6718-sEVs inhibits GC cells progression.**

**A.** Fluorescence images of GC cells with stable knockdown of circ6718 were obtained using a lentiviral vector incorporating the mCherry protein (scale bar = 100μm). **B.** An IF experiment was conducted to assess the uptake of sEVs from circ6718-knockdown GC cells by recipient GC cells (scale bar = 25μm). **C.** qRT-PCR analysis was performed to evaluate circ6718 expression in GC cells following treatment with sh-circ6718-sEVs treatment (n = 3). CCK-8 assay (**D**), colony formation assay (**E**), Transwell migration assay (**F**), and matrigel invasion assay (**G**) in PBS group, control sEVs group, and sh-circ6718-sEVs treated GC cells (scale bar = 200μm) (n = 3). **H.** Western blot analysis was performed to measure the expression of EMT markers and proliferation indices in sh-circ6718-sEVs treated GC cells. The data were plotted as Mean ± SEM. Statistical significance is indicated as follows: *p < 0.05, **p < 0.01, ***p < 0.001 by Student's t‐test for E and I; by one‐way ANOVA for C-G.


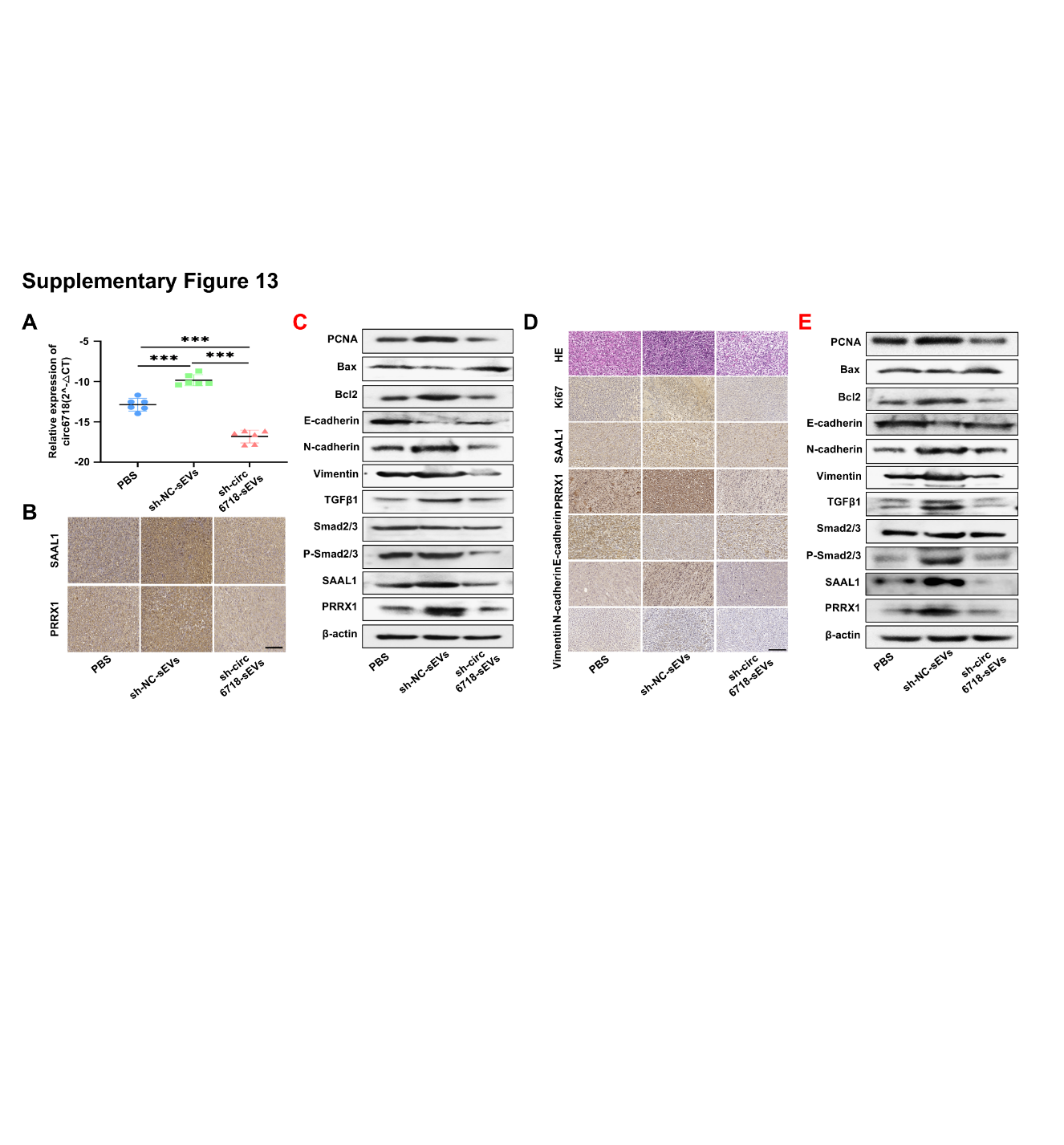


**Supplementary Figure 13. sh-circ6718-sEVs suppresses SAAL1/PRRX1 expression *in vivo*.**

1. qRT-PCR analysis was conducted to assess the expression levels of circ6718 in subcutaneous xenograft tumors derived from mice injected via the tail vein with PBS, control sEVs, or circ6718 knockdown sEVs (n = 6 mice/group). **B.** Immunohistochemical staining was performed on the tumor tissues of mouse xenograft following the tail vein injection of sh-circ6718-sEVs (scale bar = 50μm). **C.** Western blot analysis was utilized to evaluate protein expression in the tumor tissues of mice following the tail vein injection of sh-circ6718-sEVs. **D.** HE staining and immunohistochemical staining were conducted on abdominal metastatic tumors in mice injected via the tail vein with PBS group, control sEVs, or sh-circ6718-sEVs mice (scale bar = 50μm). **E.** Western blot analysis was performed on protein expression in abdominal metastatic tumor tissues of mice after tail vein injection of sh-circ6718-sEVs. The data were plotted as Mean ± SEM. Statistical significance is indicated as follows: ***p < 0.001 by one‐way ANOVA for A.


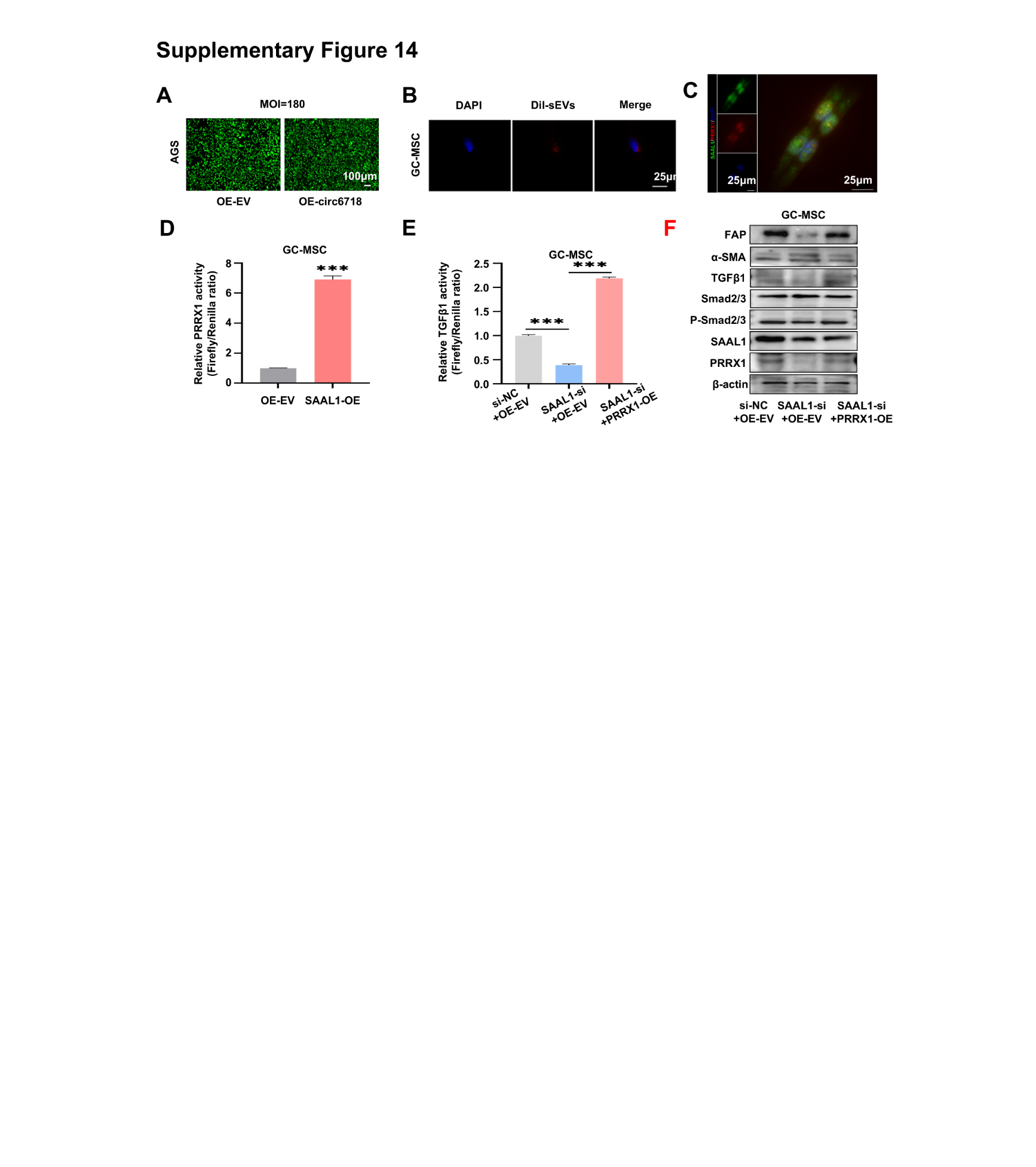


**Supplementary Figure 14. SAAL1 regulates the TGFβ1/Smad2/3 pathway in GC-MSCs via interaction with PRRX1.**

**A.** Fluorescence images depict GC cells exhibiting stable overexpression of circ6718, utilizing a lentiviral vector that incorporates the GFP protein (scale bar = 100μm). **B.** IF experiment illustrate the uptake of sEVs from circ6718 overexpressing GC cells by GC-MSCs (scale bar = 25μm). **C.** The interaction between SAAL1 and PRRX1 was confirmed through IF experiments conducted in GC-MSCs (scale bar = 25μm). **D.** The transcriptional activity of PRRX1 following SAAL1 overexpression was evaluated using a dual luciferase reporter gene assay in GC-MSCs (n = 3). **E.** A dual luciferase reporter assay was performed to assess TGFβ1 transcriptional activity following co-transfection with SAAL1 knockdown and PRRX1 overexpression plasmid (n = 3). **F.** Western blot analysis was conducted to evaluate the expression of CAF markers in GC-MSCs co-transfected with SAAL1 siRNA and the PRRX1 overexpression plasmid. The data were plotted as Mean ± SEM. Statistical significance is indicated as follows: ***p < 0.001 by Student's t‐test for D; by one‐way ANOVA for E.


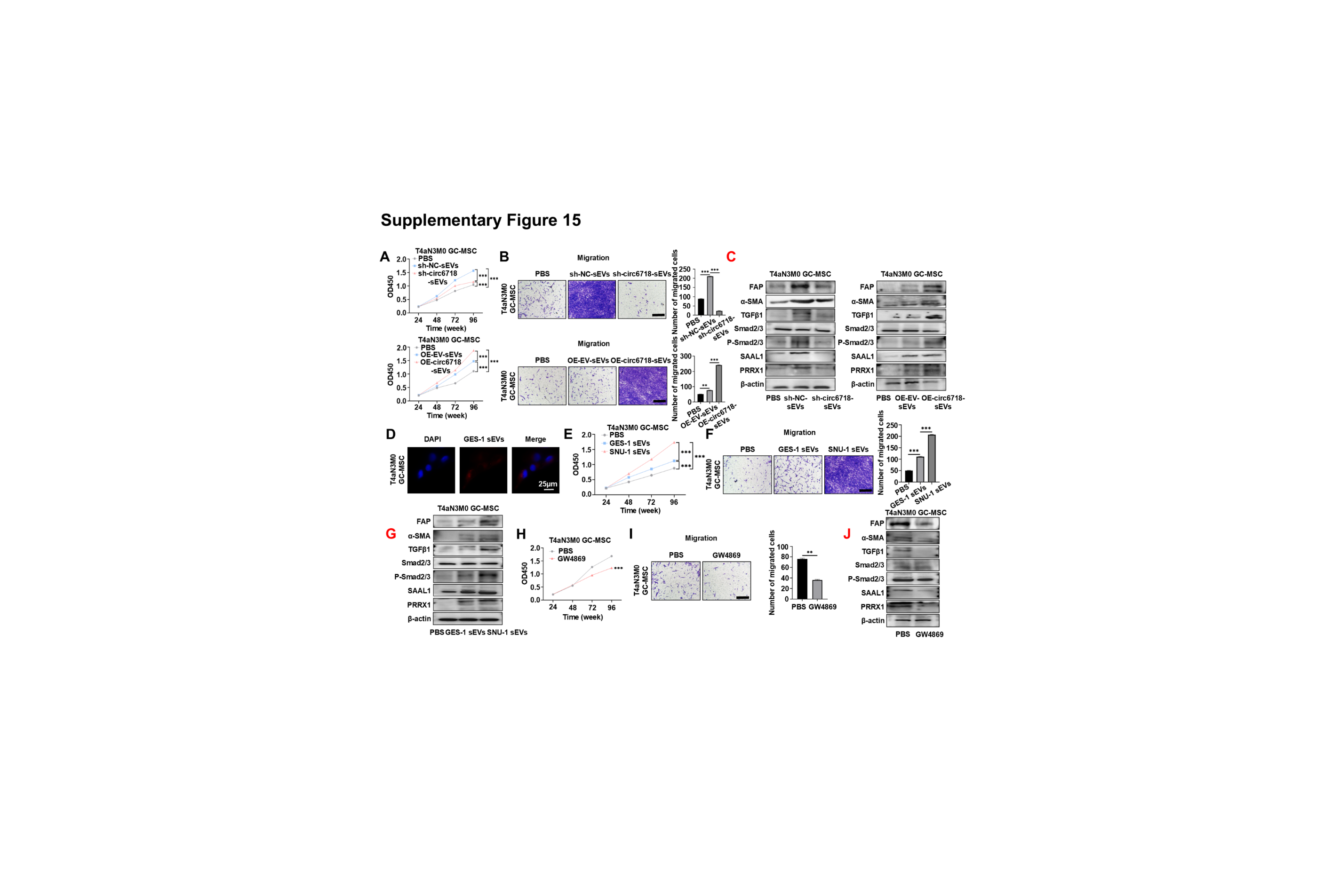


**Supplementary Figure 15. Gastric cancer cell sEVs promote transformation of GC-MSCs.**

**A.** CCK8 assay was conducted to evaluate the proliferation ability of GC-MSCs following treatment with sEVs from GC cells exhibiting circ6718 knockdown and overexpression (n = 3). **B.** Transwell migration assay assessed the migratory ability of GC-MSC after sEVs treatment from GC cells with circ6718 knockdown (scale bar = 200μm) (n = 3). **C.** Western blot analysis was employed to detect the expression of CAF markers in GC-MSCs after sEVs treatment from GC cells with circ6718 knockdown and overexpression. **D.** IF staining was utilized to visualize the uptake of sEVs from GES-1 cells with circ6718 knockdown by GC-MSCs (scale bar = 25μm). **E.** A CCK8 assay was performed to evaluate the proliferation ability of GC-MSCs following treatment with sEVs from both GES-1 and GC cells (n = 3). **F.** A Transwell migration assay was conducted to assess the migratory ability of GC-MSCs after sEVs treatment from GES-1 and GC cells (scale bar = 200μm) (n = 3). **G.** Western blot analysis was utilized to detect the expression CAF markers in GC-MSCs following sEVs treatment from both GES-1 and GC cells. **H.** A CCK8 assay was performed to evaluate the proliferation ability of GC-MSC following sEVs treatment from GC cells with circ6718 knockdown and overexpression (n = 3). **I.** A CCK8 assay was also conducted to determine the proliferation ability of GC-MSCs following treatment with GW4689 (n = 3). **J.** Western blot analysis was employed to detect CAF markers in GC-MSCs following GW4689 treatment. The data were plotted as Mean ± SEM. Statistical significance is indicated as follows: **p < 0.01, ***p < 0.001 by Student's t‐test for H and I; by one‐way ANOVA for A, B, E and F.


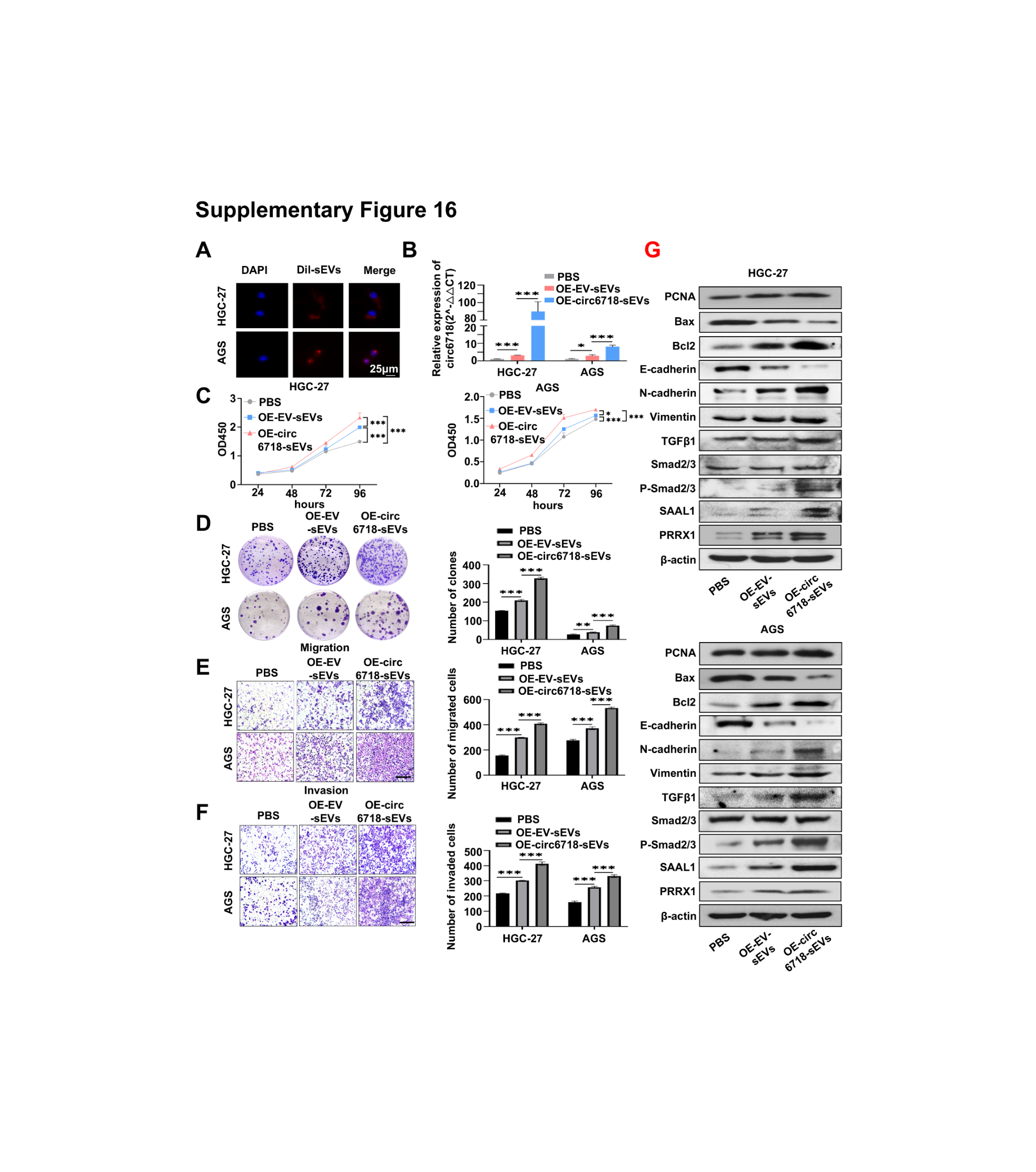


**Supplementary Figure 16. OE-circ6718-sEVs promote GC cells progression.**

**A.** IF assay demonstrates the uptake of sEVs from circ6718-overexpressing GC cells (scale bar = 25μm). **B.** qRT-PCR analysis of circ6718 expression in GC cells after treatment with OE-circ6718-sEVs (n = 3). CCK-8 assay (**C**), colony formation assay (**D**), Transwell migration assay (**E**), and matrigel invasion assay (**F**) in the PBS group, control sEVs group, and OE-circ6718-sEVs treated GC cells (scale bar = 200μm) (n = 3). **G.** Western blot analysis illustrating the expression of EMT markers and the proliferation index in OE-circ6718-sEVs treated GC cells. The data were plotted as Mean ± SEM. Statistical significance is indicated as follows: *p < 0.05, **p < 0.01, ***p < 0.001 by one‐way ANOVA for B-F.


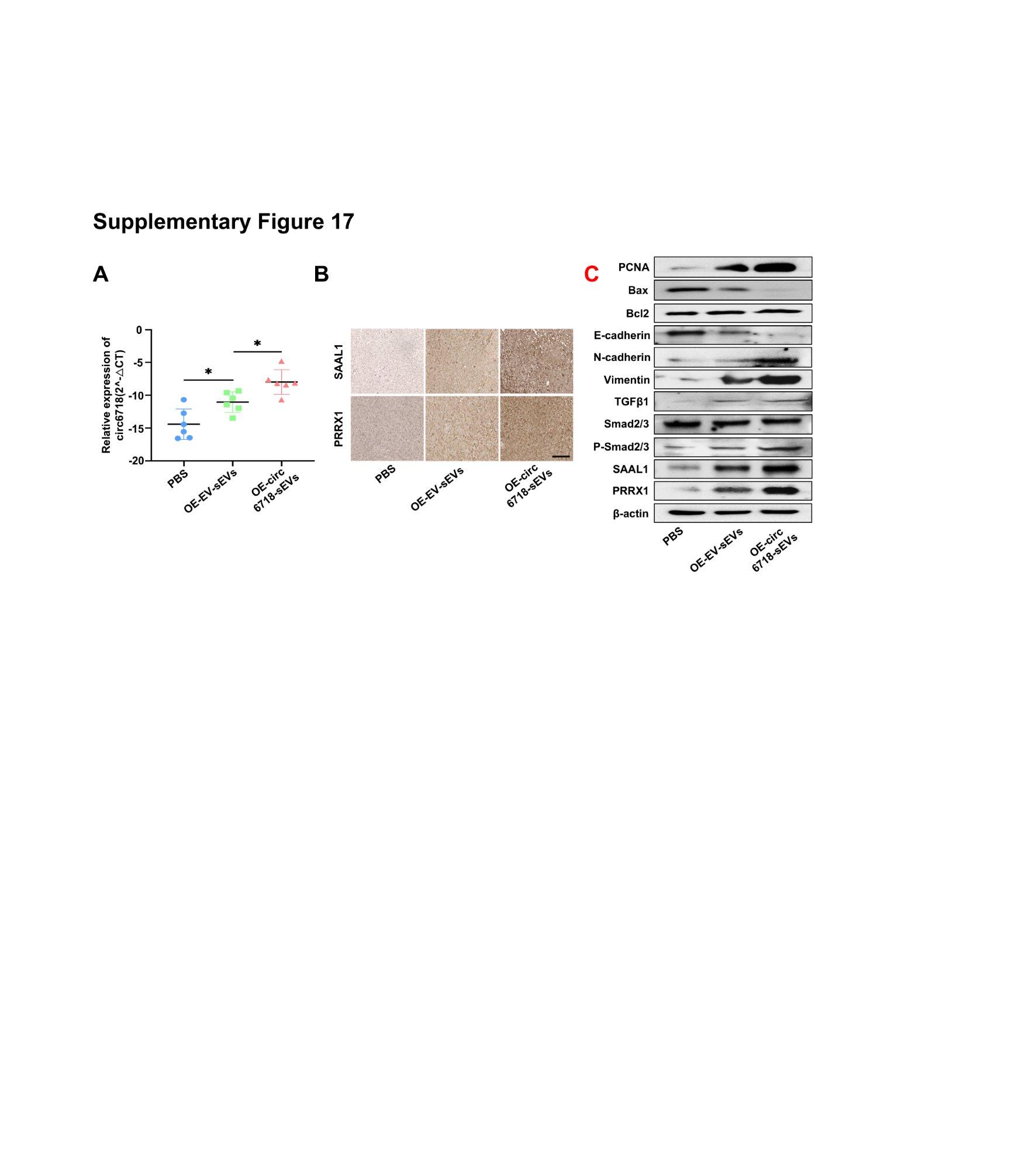


**Supplementary Figure 17. OE-circ6718-sEVs promote SAAL1/PRRX1 expression *in vivo*.**

**A.** qRT-PCR analysis of circ6718 expression in subcutaneous xenograft tumors derived from mice injected via the tail vein with PBS, control sEVs, and circ6718 overexpressing sEVs in (n = 6 mice/group). **B.** Immunohistochemical staining was performed on xenograft tumors from mice injected via the tail vein with PBS group, control sEVs, and OE-circ6718-sEVs (scale bar = 50μm). **C.** Western blot analysis was conducted to assess protein expression in mouse xenograft tumor tissues following the tail vein injection of OE-circ6718-sEVs. The data were plotted as Mean ± SEM. Statistical significance is indicated as follows: *p < 0.05 by one‐way ANOVA for A.

**
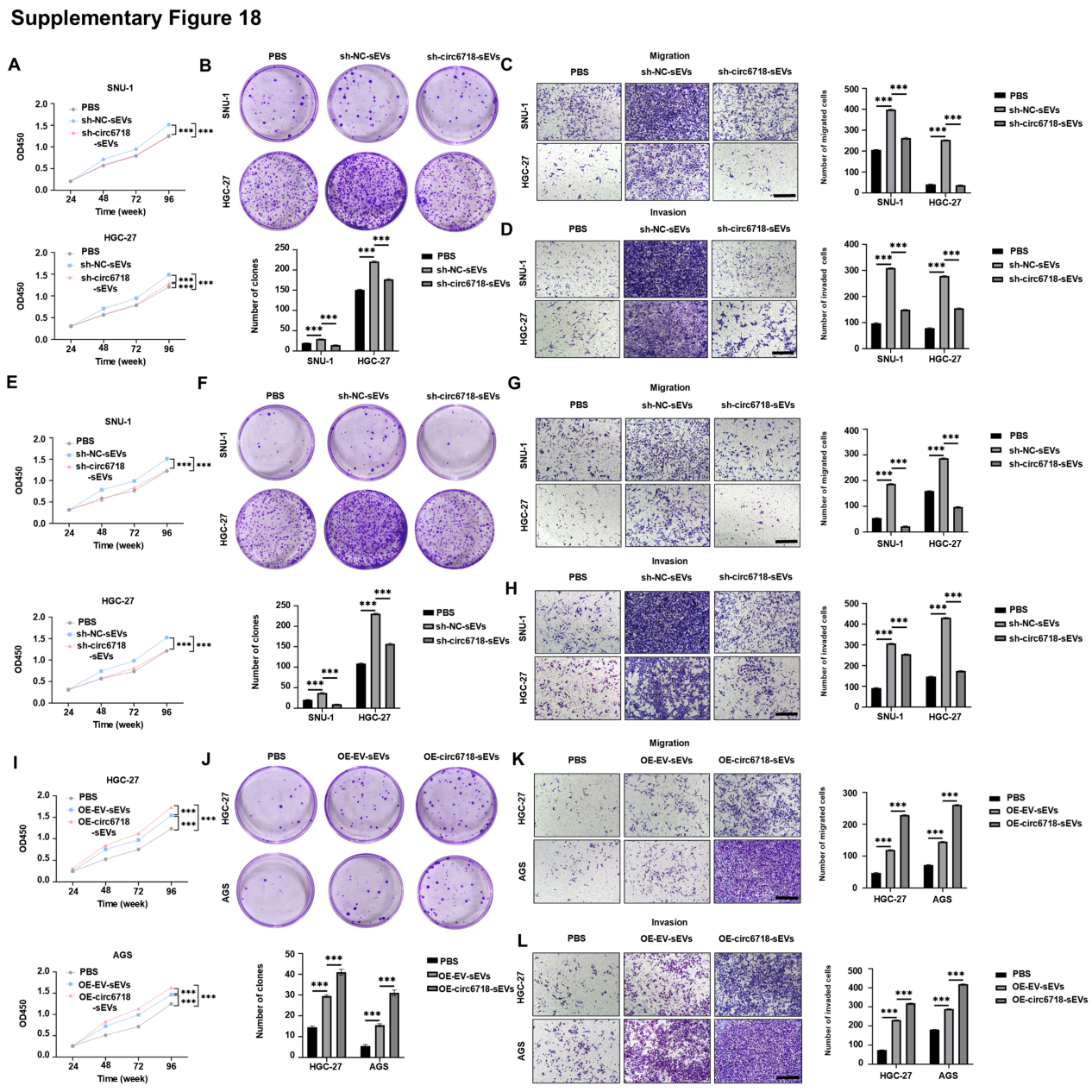
**

**Supplementary Figure 18. sEVs-circ6718 enhances the pro-tumor activity of GC-MSCs.**

The CCK-8 assay (**A**), colony formation (**B**), Transwell migration (**C**), and Matrigel invasion assay (**D**) were conducted after co-culturing sh-circ6718-sEVs-treated GC-MSCs with gastric cancer cells (scale bar = 200μm) (n = 3). CCK-8 assay (**E**), colony formation (**F**), Transwell migration (**G**), and Matrigel invasion assay (**H**) were performed on GC cells treated with sEVs derived from sh-circ6718-sEVs-processed GC-MSCs (scale bar = 200μm) (n = 3). CCK-8 assay (**I**), colony formation assay (**J**), Transwell migration (**K**), and Matrigel invasion assay (**L**) were executed on GC cells treated with sEVs derived from OE-circ6718-sEVs-processed GC-MSCs (scale bar = 200μm) (n = 3). The data were plotted as Mean ± SEM. Statistical significance is indicated as follows: ***p < 0.001 by one‐way ANOVA for A-L.

**
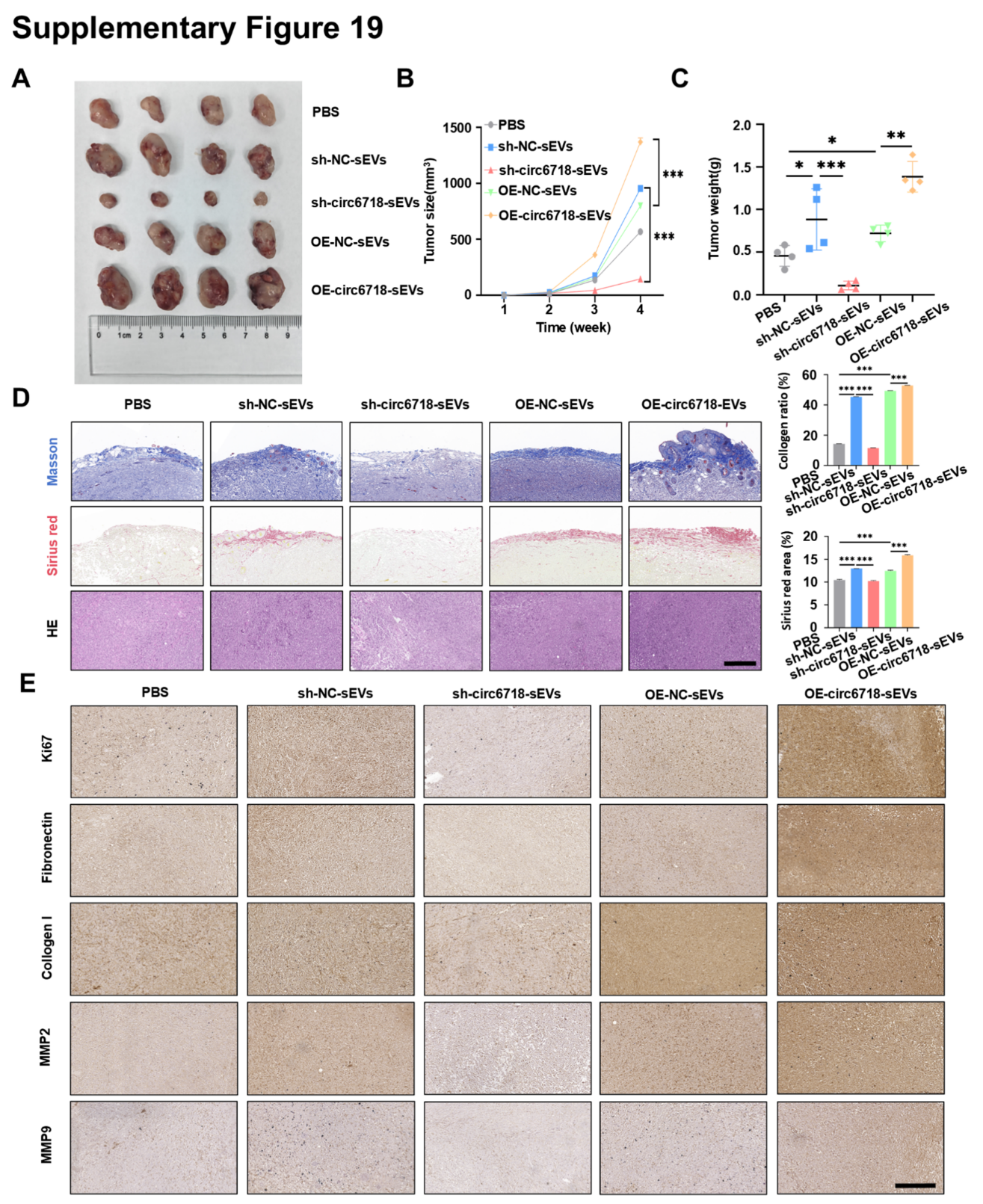
**

**Supplementary Figure 19. OE-circ6718-sEVs enhance the degree of fibrosis in subcutaneous xenograft tumors in mice.**

**A.** Measurement of subcutaneous xenograft tumor volume in mice was conducted following the co-injection of GC cells with GC-MSCs, followed by tail vein injections of PBS and sEVs respectively (n = 4 mice/group). **B.** The size of the subcutaneous xenograft tumor volume in mice was measured after the co-injection of GC cells with GC-MSCs, followed by tail vein injections of PBS and sEVs respectively (n = 4 mice/group). **C.** The methodology involved co-injecting GC cells with GC-MSCs, followed by tail vein injections of PBS and sEVs respectively) (n = 4 mice/group). **D.** Masson and Sirius red staining were utilized to assess the degree of fibrosis in subcutaneous xenograft tumors in mice (n = 4 mice/group) (scale bar = 50μm). **E.** Immunohistochemical analysis was performed to evaluate fibrosis in subcutaneously xenotransplanted tumors in mice (n = 4 mice/group) (scale bar = 50μm). The data were plotted as Mean ± SEM. Statistical significance is indicated as follows: *p < 0.05, **p < 0.01, ***p < 0.001 by one‐way ANOVA for B-D.


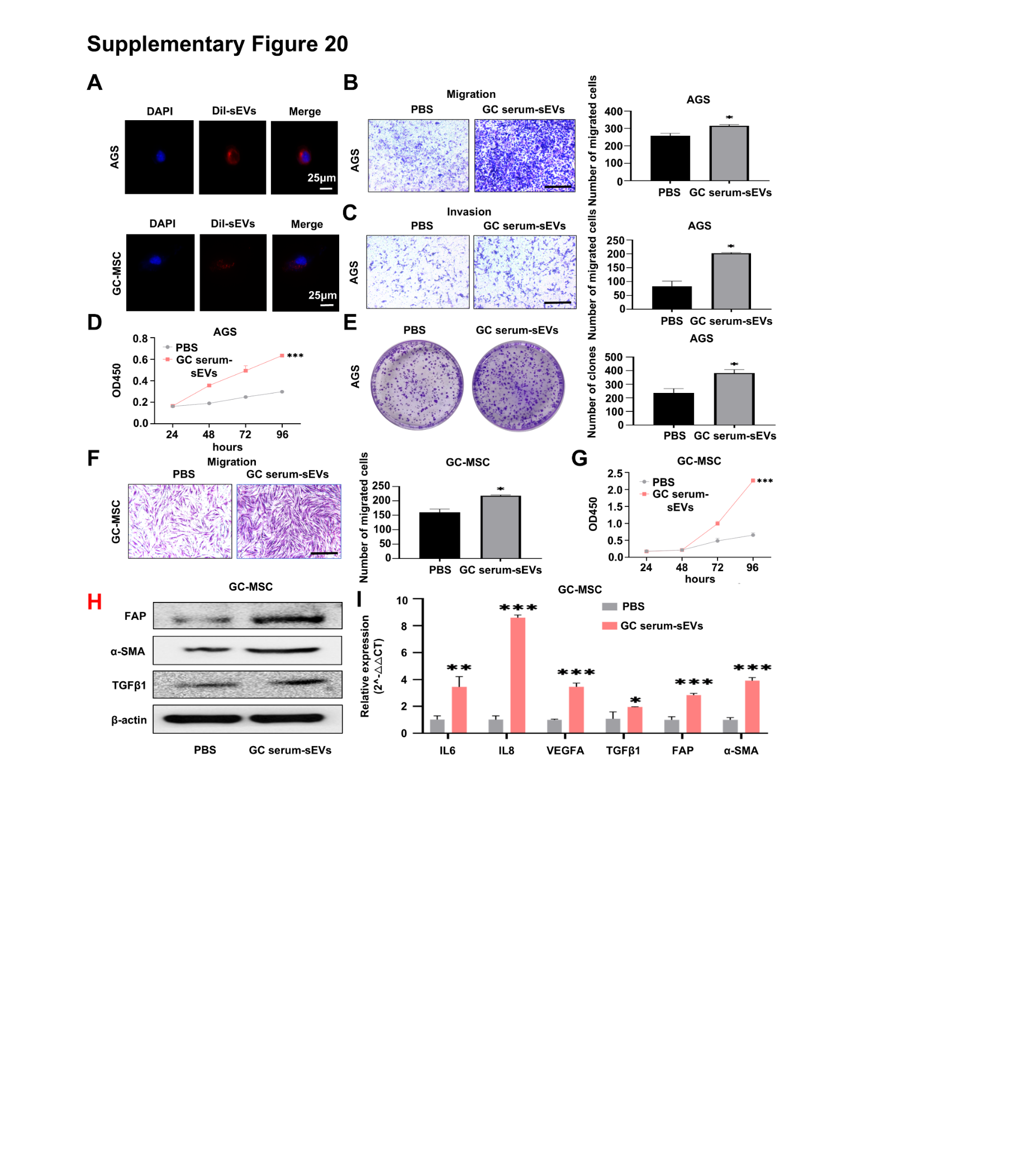


**Supplementary Figure 20. GC serum-sEVs promote GC cells progression and GC-MSCs transdifferentiation.**

**A.** IF analysis detected the uptake of serum sEVs from GC patients by GC cells and GC-MSCs (scale bar = 25μm). The Transwell migration assay (**B**), matrigel invasion assay (**C**), CCK-8 assay (**D**), and colony formation assay (**E**) were employed to assess the effects of serum sEVs from GC patients on GC cell proliferation and metastasis (scale bar = 200μm). The Transwell migration assay (**F**) and CCK-8 assay (**G**) were utilized to evaluate the impact of serum sEVs from GC patients on GC-MSC proliferation and metastasis (scale bar = 200μm) (n = 3). **H.** Western blot analysis of CAF marker expression in GC-MSCs treated with serum sEVs from GC patients. **I.** qRT-PCR analysis of CAF markers and cytokine expression in serum sEVs-treated GC-MSCs from GC patients (n = 3). The data were plotted as Mean ± SEM. Statistical significance is indicated as follows: *p < 0.05, **p < 0.01, ***p < 0.001 by Student's t‐test for B-G; by one‐way ANOVA for I.


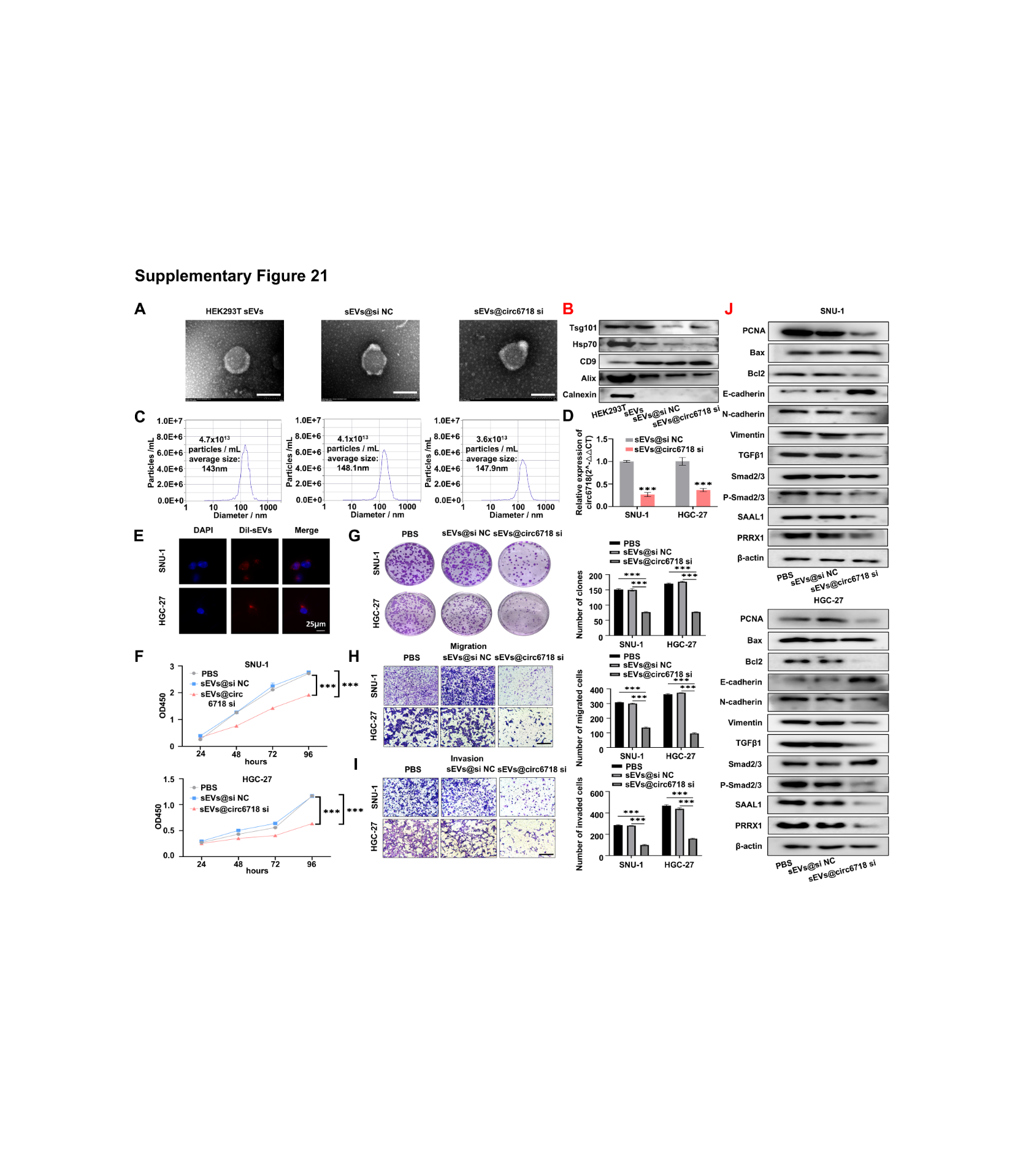


**Supplementary Figure 21. Circ6718 siRNA engineered sEVs suppress GC cells progression.**

1. The TEM analysis of engineered sEVs loaded with circ6718 siRNA is presented (scale bar = 100nm). **B.** Western blot analysis of the protein content in engineered sEVs loaded with circ6718 siRNA. **C.** Nanoparticle tracking analysis of engineered sEVs loaded with circ6718 siRNA. **D.** The qRT-PCR analysis of circ6718 expression levels in GC cells treated with engineered sEVs (n = 3). **E.** IF analysis demonstrating the uptake of engineered sEVs by GC cells (scale bar = 25μm). CCK-8 assay (**F**), colony formation assay (**G**), Transwell migration assay (**H**), and matrigel invasion assay (**I**) to evaluate the effect of engineered sEVs on GC proliferation and metastasis (scale bar = 200μm) (n = 3). **J.** Western blot analysis assessing the effect of engineered sEVs on EMT and proliferation markers in GC cells. The data were plotted as Mean ± SEM. Statistical significance is indicated as follows: ***p < 0.001 by one‐way ANOVA for D, F, G, H, and I.


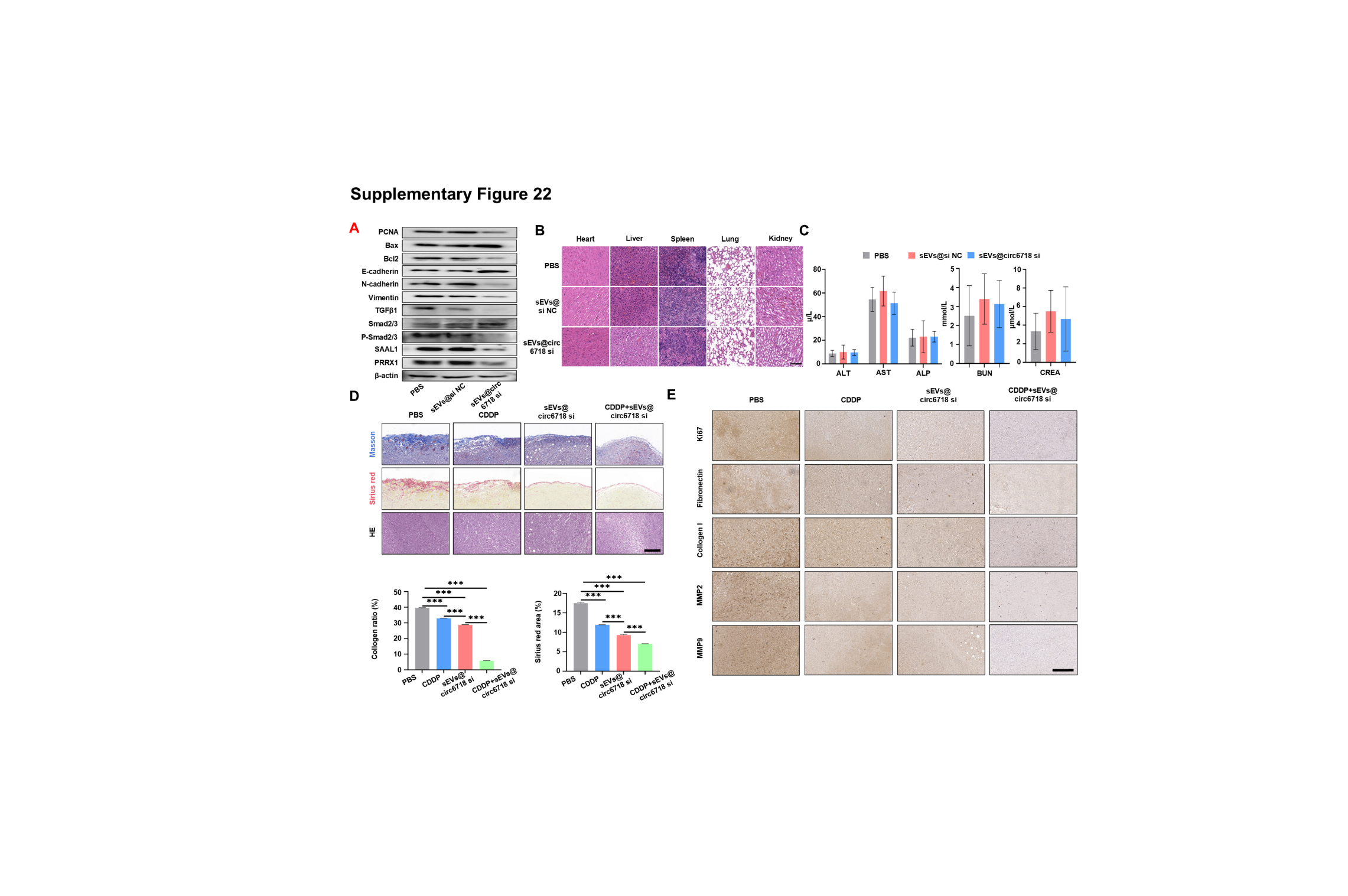


**Supplementary Figure 22. sEVs@circ6718 inhibits the degree of fibrosis in subcutaneous xenograft tumors in mice.**

1. Western blot analysis was conducted to evaluate EMT and proliferation markers in abdominal metastatic tumors derived from mice treated with PBS, control sEVs, or engineered sEVs. **B.** Histopathological examination of heart, liver, spleen, lung, and kidney sections stained with HE was performed (scale bar = 50μm). **C.** Blood biochemistry analysis was carried out to assess liver function markers (ALT, AST and ALP) and kidney function markers (BUN and CREA) (n = 6 mice/group). **D.** Masson and Sirius red staining were utilized to evaluate the extent of fibrosis in subcutaneous xenograft tumors in mice (n = 6 mice/group). **E.** Immunohistochemical analysis was performed to assess fibrosis in subcutaneously xenotransplanted tumors in mice (scale bar = 50μm). The data were plotted as Mean ± SEM. Statistical significance is indicated as follows: ***p < 0.001 by one‐way ANOVA for C and D.

**Table S1. Baseline table of clinical data of patients with GC.** Statistical significance is indicated as follows: *p<0.05 by Spearman Pearson correlation analysis.

| Features |  | Number |  | sEVs-Circ6718 | |  | *P* value（*p<0.05) |
| --- | --- | --- | --- | --- | --- | --- | --- |
|  |  |  |  | Low | High |  |  |
| Age |  |  |  |  |  |  |  |
| ＜60 |  | 43 |  | 23 | 20 |  | 0.379 |
| ≥60 |  | 142 |  | 70 | 72 |  |  |
| Gender |  |  |  |  |  |  |  |
| Male |  | 132 |  | 67 | 65 |  | 0.481 |
| Female |  | 53 |  | 26 | 27 |  |  |
| Tumor size |  |  |  |  |  |  |  |
| ＜5cm |  | 104 |  | 51 | 53 |  | 0.409 |
| ≥5cm |  | 81 |  | 42 | 39 |  |  |
| Tumor classification |  |  |  |  |  |  |  |
| Early stage |  | 73 |  | 34 | 39 |  | 0.254 |
| Advanced stage |  | 112 |  | 59 | 53 |  |  |
| Differentiation |  |  |  |  |  |  |  |
| Poor |  | 53 |  | 28 | 25 |  | 0.39 |
| Moderate |  | 132 |  | 65 | 67 |  |  |
| Lymphatic metastasis |  |  |  |  |  |  |  |
| N0 |  | 66 |  | 31 | 35 |  | 0.303 |
| N1-3 |  | 119 |  | 62 | 57 |  |  |
| Invasion depth |  |  |  |  |  |  |  |
| T1 and T2 |  | 53 |  | 22 | 31 |  | 0.089 |
| T3 and T4 |  | 132 |  | 71 | 61 |  |  |
| Distal metastasis |  |  |  |  |  |  |  |
| M0 |  | 140 |  | 64 | 76 |  | **0.022*** |
| M1 |  | 45 |  | 29 | 16 |  |  |
| TNM stage |  |  |  |  |  |  |  |
| Ⅰ and Ⅱ |  | 65 |  | 29 | 36 |  | 0.177 |
| Ⅲ and Ⅳ |  | 119 |  | 63 | 56 |  |  |
| Nerve invasion |  |  |  |  |  |  |  |
| No |  | 106 |  | 50 | 56 |  | 0.179 |
| Yes |  | 78 |  | 43 | 35 |  |  |

**Table S2. The sequences of primers for qRT-PCR.**

| Gene | Sequence | Temperature (℃) |
| --- | --- | --- |
| Circ6718 | F:5’-CTGAAAACATTACTTATCTGGC-3’ | 54 |
|  | R:5’-CTTCTGAATTGCTTGAACTCTA-3’ |  |
| β-actin | F:5’-CACGAAACTACCTTCAACTCC-3’ | 60 |
|  | R:5’-CATACTCCTGCTTGCTGATC-3’ |  |
| hsa-miR-561-3p | F:5’-ACCGAGGTCAAAGTTTAAGATCC-3’ | 60 |
|  | R:5’-CAGTGCAGGGTCCGAGGT-3’ |  |
|  | RT:5’-GTCGTATCCAGTGCAGGGTCCGAGGTATTCGCACTGGATACGACACTTCA-3’ |  |
| hsa-miR-29b-3p | F:5’-AACACGTGTAGCACCATTTGAA-3’ | 60 |
|  | R:5’-CAGTGCAGGGTCCGAGGT-3’ |  |
|  | RT:5’-GTCGTATCCAGTGCAGGGTCCGAGGTATTCGCACTGGATACGACAACACT-3’ |  |
| hsa-miR-3163 | F:5’-CCGCTCGTATAAAATGAGGGC-3’ | 60 |
|  | R:5’-CAGTGCAGGGTCCGAGGT-3’ |  |
|  | RT:5’-GTCGTATCCAGTGCAGGGTCCGAGGTATTCGCACTGGATACGACGTCTTA-3’ |  |
| hsa-miR-520g-3p | F:5’-AACACGCACAAAGTGCTTCC-3’ | 60 |
|  | R:5’-CAGTGCAGGGTCCGAGGT-3’ |  |
|  | RT:5’-GTCGTATCCAGTGCAGGGTCCGAGGTATTCGCACTGGATACGACACACTC-3’ |  |
| U6 | F:5’-CGCTTCGGCAGCACATATAC-3’ | 60 |
|  | R:5’-TTCACGAATTTGCGTGTCATC-3’ |  |
|  | RT:5’-GTCGTATCCAGTGCAGGGTCCGAGGTATTCGCACTGGATACGACAAAATA-3’ |  |
| SAAL1 | F:5’-CGGTGGCTCAGGGAGTAAAG-3’ | 60 |
|  | R:5’-AGTCATCAGCAAGCGCCTTA-3’ |  |
| RBM23 | F:5’-CCGGAACTGATCTGACAGGATG-3’ | 60 |
|  | R:5’-ACGACTGCGCTTTCTATCCC-3’ |  |
| GTF2E1 | F:5’-GAGTGTTCCAGGATTCGCCT-3’ | 60 |
|  | R:5’-GGCTAACCGCTTCAATGCTG-3’ |  |
| TXNDC12 | F:5’-AAGATTTCAGCCCTGACGGG-3’ | 60 |
|  | R:5’-TCTCCGCTGGATCCACAAAC-3’ |  |
| PRRX1 | F:5’-TGATGCTTTTGTGCGAGAAGA-3’ | 60 |
|  | R:5’-AGGGAAGCGTTTTTATTGGCT-3’ |  |
| TGFβ1 | F:5’-ATGGTGGAAACCCACAACGA-3’ | 60 |
|  | R:5’-GCTGAGGTATCGCCAGGAAT-3’ |  |
| TGFβ1  promoter  primers | F:5’-TGTTTTCCCTCACAGCAA-3’ | 60 |
|  | R:5’-AAGCAAATTATAGGGTAGATCA-3’ |  |
| IL-6 | F:5’-TGAAAGCAGCAAAGAGGCACT-3’ | 60 |
|  | R:5’-TACCTCAAACTCCAAAAGACCAG-3’ |  |
| IL-8 | F:5’-ACTCCAAACCTTTCCACCCC-3’ | 60 |
|  | R:5’-TTCTCAGCCCTCTTCAAAAACT-3’ |  |
| VEGFA | F:5’-TATGCGGATCAAACCTCACC-3’ | 60 |
|  | R:5’-CACCAACGTACACGCTCC-3’ |  |
| FAP | F:5’-CATCTGGAAAAATGAAGACTTGGGT-3’ | 60 |
|  | R:5’-GGGCGTAAGACAATGCACATC-3’ |  |
| α-SMA | F:5’-CTTCCCAGACTTCCGCTTCA-3’ | 60 |
|  | R:5’-CGCTGGAGGACTTGCTTTTC-3’ |  |

**Table S3. Reagents used in this study.**

| **REAGENT or RESOURCE** | **SOURCE** | **IDENTIFIER** |
| --- | --- | --- |
| **Antibodies** |  |  |
| Rabbit anti-CD9 antibody | Abcam | Cat #ab236630; RRID:AB_2922400 |
| Rabbit anti-CD81 antibody | Proteintech | Cat #27855-1-AP; RRID:AB_2880995 |
| Rabbit anti-Alix antibody | Abcam | Cat #ab275377; RRID:AB_3644262 |
| Mouse anti-Hsp70 antibody | Abcam | Cat #ab2787; RRID:AB_303300 |
| Rabbit anti-Calnexin antibody | Abcam | Cat #ab75801; RRID:AB_1310022 |
| Mouse anti-CD63 antibody | Abcam | Cat #ab59479; RRID:AB_940915 |
| Rabbit anti-Tsg101 antibody | Proteintech | Cat #28283-1-AP; RRID:AB_2881104 |
| Rabbit anti-β-actin antibody | Abclonal | Cat #AC026; RRID:AB_2768234 |
| Rabbit anti-Bax antibody | Cell Signaling Technology | Cat #2772; RRID:AB_10695870 |
| Mouse anti-Bcl2 antibody | Cell Signaling Technology | Cat #15071; RRID:AB_2744528 |
| Rabbit anti-PCNA antibody | Cell Signaling Technology | Cat #13110; RRID:AB_2636979 |
| Rabbit anti-E-Cadherin antibody | Bioworld | Cat #BS1098; RRID:AB_1664064 |
| Rabbit anti-N-Cadherin antibody | Cell Signaling Technology | Cat #13116; RRID:AB_2687616 |
| Rabbit anti-Vimentin antibody | Bioworld | Cat #BS1491; RRID:AB_1663663 |
| Rabbit anti-SAAL1 antibody | Abclonal | Cat #A13183; RRID:AB_2760034 |
| Rabbit anti-PRRX1 antibody | Abclonal | Cat #A10237; RRID:AB_2757762 |
| Rabbit Anti-TGFβ1 antibody | Abcam | Cat #ab215715; RRID:AB_2893156 |
| Rabbit anti-Smad2/3 antibody | Abclonal | Cat #A7536; RRID:AB_2768063 |
| Rabbit anti-p-Smad2/3 antibody | WanLeiBio | Cat #WL02305; RRID:AB_3697722 |
| Rabbit anti-FAP antibody | Abclonal | Cat #A23789; RRID:AB_3492061 |
| Mouse anti-α-SMA antibody | Boster | Cat #BM0002; RRID:AB_2811044 |
| Mouse anti-CD44 antibody | BioLegend | Cat #397517; RRID:AB_2888763 |
| Mouse anti-CD73 antibody | BioLegend | Cat #127219; RRID:AB_2716075 |
| Mouse anti-CD45 antibody | BioLegend | Cat #982316; RRID:AB_2876779 |
| Mouse anti-CD14 antibody | BioLegend | Cat #982502; RRID:AB_2616906 |
| Mouse anti-CD34 antibody | BioLegend | Cat #343503; RRID:AB_1731923 |
| Rabbit anti-Ki67 antibody | Cell Signaling Technology | Cat #12202; RRID:AB_2620142 |
| Goat anti-rabbit lgG secondary antibody | Invitrogen | Cat #31460; RRID:AB_228341 |
| Goat anti-mouse lgG secondary antibody | Invitrogen | Cat #31430; RRID:AB_228307 |
| Fluorescein (FITC)–conjugated Goat anti-rabbit lgG(H+L) | Proteintech | Cat #SA00003-2; RRID:AB_2890897 |
| Cy3-conjugated Goat anti-mouse lgG (H+L) (AS008) | Abclonal | Cat #AS008; RRID:AB_2769088 |
| Normal rabbit IgG | Millipore | Cat #I5381; RRID:AB_1163670 |
| Rat anti-Ago2 antibody | Millipore | Cat #SAB4200085;  RRID:AB_10600719 |
| **Bacterial and virus strains** |  |  |
| hsa_circ_0006718 shRNA LV10N(U6/mCherry&Puro) | GenePharm | N/A |
| pSLenti-EF1-EGFP-F2A-Puro-CMV-S-hsa_circ_0006718-WPRE | Obio Technology Corp | N/A |
| **Biological samples** |  |  |
| Male BALB/c nu/nu mice | Cavens | N/A |
| **Chemicals, peptides, and recombinant proteins** |  |  |
| F12 medium | Meilunbio | Cat #MA0214-2 |
| RPMI-1640 medium | Meilunbio | Cat #MA0215-2 |
| High glucose DMEM medium | Meilunbio | Cat #MA0212-2 |
| Fetal bovine serum | Excell | Cat #FSP050 |
| Trizol | ThermoFisher Scientific | Cat #15596026 |
| RNase R enzyme | Epicentre | Cat #RNR07250 |
| DAPI | Sigma | Cat #D9542 |
| Plasmids, siRNA, miRNA mimics and inhibitors | GenePharma | N/A |
| Lipofectamine 2000 | Invitrogen | Cat #11668019 |
| Dual-luciferase reporter assay kit | Vazyme | Cat #DL101-01 |
| Normal rabbit IgG | Cell Signaling Technology | Cat #2729 |
| Maxima SYBR Green qPCR premix | Vazyme | Cat #Q511-02 |
| Co-IP buffer | Beyotime | Cat #P0013 |
| Protein A agarose beads | Beyotime | Cat #P2012 |
| CCK-8 solution | Vazyme | Cat #A311-02 |
| Transwell chambers | Corning | Cat #3422 |
| Matrix gel | BD Biosciences | Cat #356234 |
| RIPA lysis buffer | ThermoFisher Scientific | Cat #89901 |
| Polyvinylidene fluoride (PVDF) membranes | Millipore | Cat #IPVH00010 |
| α-MEM medium | Gibco | Cat #C12571500BT |
| **Critical commercial assays** |  |  |
| miRNeasy serum/plasma kit | Qiagen | Cat #217184 |
| ExoQuick exosome precipitation solution | SBI | Cat #EXOQ20A-1 |
| Universal Genomic DNA Extraction Kit | Tiangen | Cat #DP705 |
| PrimeScript RT reagent kit | Vazyme | Cat #R312-01/02 |
| PARIS^TM^ Kit | Life Technologies | Cat #AM1921 |
| RNA FISH kit | GenePharma | Cat #F32202/50 |
| Pierce agarose ChIP kit | Thermo Scientific | Cat #26156 |
| EZ-Magna RIP™ RNA binding protein immunoprecipitation kit | Millipore | Cat #RIP-12RXN |
| Streptavidin-biotin complex (SABC) kit | Boster | Cat #SA1020 |
| GW4869 | MedChemexpress | Cat #HY-19363 |
| Cisplatin | MedChemexpress | Cat #HY-17394 |
| DIR | MedChemexpress | Cat #HY-D1048 |
| **Experimental models: Cell lines** |  |  |
| AGS | Shanghai EK-Bioscience Biotechnology | Cat #CC-Y1045; RRID:CVCL_0139 |
| SNU-1 | Shanghai EK-Bioscience Biotechnology | Cat #CC-Y1478; RRID:CVCL_0099 |
| HGC-27 | Shanghai EK-Bioscience Biotechnology | Cat #CC-Y1228; RRID:CVCL_1279 |
| GES-1 | Shanghai EK-Bioscience Biotechnology | Cat #CC-Y1572; RRID:CVCL_EQ22 |
| HEK293T | Shanghai EK-Bioscience Biotechnology | Cat #CC-Y1010; RRID:CVCL_0063 |
| **Experimental models: Organisms/strains** |  |  |
| Mouse: BALB/c-Nude | Changzhou Cavens Model Animal | Cat #C000103 |
| **Deposited data** |  |  |
| sEVs circRNA microarray raw data | GEO database | GSE302451 |
| **Oligonucleotides** |  |  |
| The sequences of primers for qRT-PCR | This paper | Table S2 |
